# Novel benzo chromene derivatives: design, synthesis, molecular docking, cell cycle arrest, and apoptosis induction in human acute myeloid leukemia HL-60 cells

**DOI:** 10.1080/14756366.2022.2151592

**Published:** 2022-12-02

**Authors:** Rania H. Abd El-Hameed, Mosaad S. Mohamed, Samir M. Awad, Bardes B. Hassan, Marwa Abd El-Fattah Khodair, Yara E. Mansour

**Affiliations:** a Pharmaceutical Organic Chemistry Department, Faculty of Pharmacy, Helwan University, Cairo, Egypt; b Department of Pathology, Faculty of Veterinary Medicine, Cairo University, Giza, Egypt

**Keywords:** Benzocoumarin, apoptosis, cell cycle arrest, AML, HL-60

## Abstract

A series of benzo[*h*]chromenes, benzo[*f*]chromenes, and benzo[*h*]chromeno[2,3-*d*]pyrimidines were prepared. All the newly synthesised compounds were selected by National Cancer Institute for single-dose testing against 60 cell lines. Benzo[*h*]chromenes **5a** and **6a** showed promising anti-cancer activity and selected for the five-dose testing. Compounds **5a** and **6a** suppressed cell growth in HL-60 by the induction of cell cycle arrest, which was confirmed using flow cytometry and Annexin V-FITC/PI assays showed at the G1/S phase by regulating the expression of CDK-2/CyclinD1, triggering cell apoptosis by activating both the extrinsic (Fas/Caspase 8) and intrinsic (Bcl-2/Caspase 3) apoptosis pathways, which were determined by the western blot. Benzo[*h*]chromenes **5a** and **6a** decreased the protein expression levels of Bcl-2, CDK-2, and CyclinD1 and increased the expression of caspase 3, caspase 8, and Fas. *In silico* molecular analysis of compounds **5a** and **6a** in CDK-2 and Bcl-2 was performed.

## Introduction

Acute myeloid leukaemia (AML), one of the most prevalent forms of haematologic cancer, is characterised by clonal, proliferative, and improperly or poorly differentiated myeloid cells that infiltrate the bone marrow, blood, or extra medullary organs^1–3^. AML incidence increased recently as 20,050 newly estimated cases of AML and 11,540 estimated deaths were recorded in 2022 in the United States^4^.

Chemotherapy is the most used treatment for AML, either alone or in combination with other medications such as doxorubicin, idarubicin, or anthracyclines. However, the prognosis for AML remains poor. Furthermore, traditional chemotherapy is extremely toxic and poorly tolerated. As a result, novel treatment strategies must be developed to improve the therapeutic efficacy for AML^5^.

**Figure 1.**
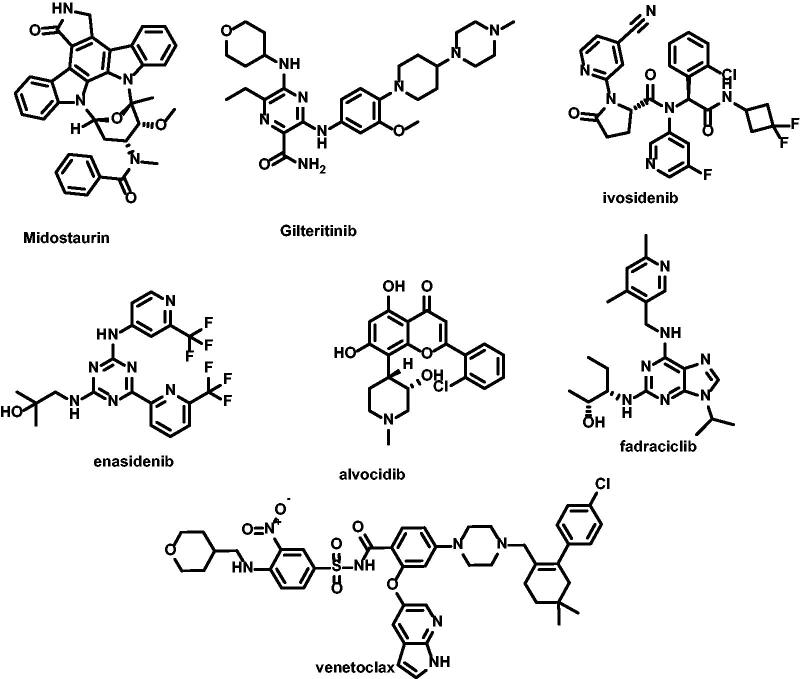
Multi-targeting synthetic small molecules for treatment of AML advanced to clinical trials.

Several novel agents acting through distinct molecular targets, identified in the pathophysiology of AML, are being investigated alone or combined with conventional chemotherapy, e.g. FLT3 inhibitors (midostaurin^6^, gilteritinib^7^), IDH1/2 inhibitors (ivosidenib^8^, enasidenib^9^^,^^10^), BCL-2 inhibitors (venetoclax^11^^,^^12^), and cyclin-dependent kinase (CDK) inhibitors (CDKi; alvocidib^13–15^ fadraciclib^16^), which cause cell cycle arrest and induce apoptosis (Figure 1).

Apoptosis is fundamental to the development and maintenance of cellular homeostasis within tissues. Apoptosis signalling cascades are divided into two major pathways: the death-receptor (Fas/FasL/Caspase-8) (extrinsic) and mitochondria-mediated (Bax/Bcl-2/Caspase-9) (intrinsic) pathways.^17^ The mitochondrial intrinsic apoptotic pathway is playing the most important role in cancer therapy. BCL-2 (B-cell lymphoma-2) family proteins regulate the intrinsic apoptotic pathway via mitochondria following cell death^18^^,^^19^, this family includes both anti- and pro-apoptotic proteins. Anti-apoptotic proteins, particularly BCL-2^20–23^, BCL-xL^24^, and MCL-1^25–29^ have been found to be overexpressed in various types of cancer, and played essential roles in carcinogenesis in various tumour models^18^^,^^30–34^. Inhibitors of BCL-2 family proteins act directly on the apoptotic machinery^35^ and apoptosis caused by modulating Bcl-2 family members has a significant role in the effect of various therapeutic approaches for AML^36^. According to a previous study, the orphan nuclear receptor Nur77 directly interacts with Bcl-2 in the mitochondria, causing a conformational change that exposes the BH3 domain of Bcl-2 and changes its anti-apoptotic function to proapoptotic^37^. Furthermore, in another study, N,N′-dimethylquinacridone (DMA) promoted apoptosis in THP-1 leukaemia cells by decreasing the level of the anti-apoptotic protein Bcl-2^38^. Both the intrinsic and extrinsic apoptotic pathways converge and activate caspases, which are cysteine proteases and key regulators of apoptosis. The intrinsic apoptotic pathway activates caspase-9^39^^,^^40^, which in turn activates caspase-3. The extrinsic apoptotic pathway (death receptor pathway) is induced by death receptors activated by their ligands, such as Fas, a cysteine-rich type I receptor of the tumour necrosis factor (TNF) receptor family, and its ligand FasL, a type II membrane protein involved in the TNF family. The Fas/FasL pathway activates caspase-8^41^, which is then involved in the cleavage and activation of caspase-3 and activates the intrinsic apoptotic pathway.

Chromene-based natural and synthetic molecules have made major contributions to the development of anti-neoplastic therapies against a variety of human cancers^42^^,^^43^. For 4H-chromenes, the discovery of HA14-1 was a major step forward in the development of anti-cancer medicines. Some evidence suggests that it works synergistically with flavopiridol in order to suppress Bcl-2 via disrupting down the connection between Bax and Bcl-2^44–46^.

As a continuation of our previous trials^47–50^ to develop novel cytotoxic agents, we intended to synthesise new benzo[*h*]chromene, benzo[*f*]chromene, and benzo[*h*]chromeno[2,3-*d*]pyrimidine derivatives in the current study and investigate the mechanism of benzocoumarin-induced apoptosis by detecting both intrinsic and extrinsic pathways. Then, we investigated the effect of benzocoumarin on cell cycle arrest of HL-60 cells. Our findings could pave the way for the use of benzocoumarin as a potential therapeutic candidate for AML.

## Materials and methods

### Synthesis of lead compounds

All commercial chemicals used as starting materials and reagents in this study were purchased from Merck (Darmstadt, Germany) and were of reagent grade. All melting points were uncorrected and measured using Electro-thermal IA 9100 apparatus (Shimadzu, Japan); IR spectra were recorded as potassium bromide pellets on a Perkin-Elmer 1650 spectrophotometer (USA), Faculty of Science, Cairo University, Cairo, Egypt. ^1^H-NMR spectra were determined on a Varian Mercury (300 MHz) spectrometer (Varian UK) and chemical shifts were expressed as ppm against TMS as internal reference (The Main Chemical Warfare Laboratories, Almaza, Cairo, Egypt). Mass spectra were recorded on 70 eV (EI Ms-QP 1000 EX, Shimadzu, Japan), Faculty of Science, Cairo University, Cairo, Egypt. Microanalyses were operated using Vario, Elmentar apparatus (Shimadzu, Japan), Organic Microanalysis Unit, Faculty of Science, Cairo University, Cairo, Egypt. Column Chromatography was performed on (Merck) Silica gel 60 (particle size 0.06–0.20 mm). All compounds prepared in this paper are new and confirmed with spectral data except **1a–c** were previously reported^51–53^.

#### Synthesis of N-(3-cyano-4-phenyl-4H-benzo[h]chromen-2-yl)formamide (2)

2-Amino-4-phenyl-4H-benzo[h]chromene-3-carbonitrile (**1a**, 1 g) was heated under reflux in formic acid (20 ml) for 15 hs, cooled, poured onto ice to give precipitate which was filtered, dried and recrystallized from ethanol to give compound **2.**

Yield: 70%; m.p.: 125–127 °C; IR (KBr) υ (cm^−1^): 3344 (NH), 2897 (CH = O), 2229 (C≡N), 1724(C = O), 1298 (C–O); MS (EI) *m/z*: 326 (M^+^, 34%), 297 (M–C = O, 100%); ^1^H-NMR (DMSO-d_6_, 300 MHz) δ (ppm): 4.67 (s, 1H, C4–H), 6.51–8.40 (m, 11 H, Ar–H), 8.90 (s, 1H, NH, D_2_O exchangeable), 10.00 (s, 1H, CHO); Anal. Calcd. for C_21_H_14_N_2_O_2_ (326.35): C, 77.29; H, 4.32; N, 8.58%. Found: C, 77.51; H, 3.99; N, 8.26%.

#### Synthesis of N'-anilino-N-(3-cyano-4-phenyl-4H-benzo[h]chromen-2-yl) formamidine (3)

Equimolar amounts of formamide derivative **2** (0.1 mol) and phenyl hydrazine were heated under reflux in ethanol (20 mL) for 24 h, cooled, poured onto ice to give precipitate which was filtered, dried and recrystallized from ethanol to give compound **3.**

Yield: 72%; m.p.: 160–162 °C; IR (KBr) υ (cm^−1^): 3448 (NH), 2144 (C≡N), 1607(C = N), 1306(C–O); MS (EI) *m/z*: 416 (M^+^, 70.9%); ^1^H-NMR (DMSO-d_6_, 300 MHz) δ (ppm): 4.21 (s, 1H, C4-H), 6.40 (s, 1H, CH = N), 6.81–8.50 (m, 16 H, Ar–H), 8.82 (s, 1H, NH, D_2_O exchangeable), 8.93 (s, 1H, NH, D_2_O exchangeable); Anal. Calcd. for C_27_H_20_N_4_O (416.47): C, 77.87; H, 4.84; N, 13.45%. Found: C, 77.53; H, 4.70; N, 13.20%.

#### Synthesis of 5-phenyl-5H-benzo[h]chromeno[2,3-d]pyrimidin-4-one (4)

The appropriate 2-amio-benzo[h]chromene-3-carbonitrile **1a** (0.01 mol) was heated under reflux in 20 mL formic acid (85%) in the presence of 2 mL (HCl/H_2_O) for 30 h, cooled, poured onto ice to give precipitate, which was filtered, dried and recrystallized from ethanol to give compound **4.**

Yield: 65%; m.p.: 117–119 °C; IR (KBr) υ (cm^−1^): 3400 (NH), 1724 (C = O), 1608 (C = N); MS (EI) *m/z*: 326 (M^+^, 13.3%); ^1^H-NMR (DMSO-d_6_, 300 MHz) δ (ppm): 4.50 (s, 1H, C5–H), 6.51 (s, 1H, NH, D_2_O exchangeable), 6.60–8.22 (m, 12H, Ar–H); Anal. Calcd for C_21_H_14_N_2_O_2_ (326.9): C, 77.29; H, 4.32; N, 8.58%. Found: C, 77.40; H, 4.22; N, 8.76%.

#### General procedure for the synthesis of compounds 5a–c

A mixture of equimolar amounts of the appropriate benzo[h]chromene-3-carbonitrile **1a–c** (0.01 mol) and NH_2_OH.HCl (0.01 mol, 0.33 g) with Na_2_CO_3_ (0.05 mol, 5.3 g) was heated under reflux in absolute ethanol (20 mL) for 20 h, cooled, poured onto ice to give precipitate, which was filtered, dried, and recrystallized from methanol to give compounds **5a–c.**

##### 2-Amino-4-phenyl-N-hydroxy-4H-benzo[h]chromene-3-carboxamidine (5a)

Yield: 60%; m.p.: 210–212 °C; IR (KBr) υ (cm^−1^):3423 (broad OH), 1614 (C = N), 1312 (C–O); MS (EI) *m/z*: 331 (M^+^, 86.3%); ^1^H-NMR (DMSO-d_6_, 300 MHz) δ (ppm): 4.36 (s, 1H, OH, D_2_O exchangeable), 5.20 (s, 1H, C4–H), 5.41 (s, 2H, NH_2_, D_2_O exchangeable), 7.0–8.30 (m, 11H, Ar–H), 9.41 (s, 1H, NH, D_2_O exchangeable), 9.82 (s, 1H, NH, D_2_O exchangeable); ^13^C NMR δ: 160.66 (HN = C), 146.14 (=C–NH_2_), 143.20 (C3), 129.19, 128.87, 128.12, 127.42, 127.26, 127.16, 126.67, 124.39, 123.22, 121.18, 120.98, 118.40 (aromatic Cs), 56.77 (C-4); Anal. Calcd for C_20_H_17_N_3_O_2_ (331.37): C, 72.49; H, 5.17; N, 12.68%. Found: C, 72.40; H, 4.95; N, 12.30%.

##### 2-Amino-4–(4-methoxyphenyl)-N-hydroxy-4H-benzo[h]chromene-3-carboxamidine (5b)

Yield: 63%; m.p.: 220–222 °C; IR (KBr) υ (cm^−1^): 3540 (broad OH), 1604 (C = N), 1330 (C–O); MS (EI) *m/z*: 361 (M^+^, 38.7%); ^1^H-NMR (DMSO-d_6_, 300 MHz) δ (ppm): 3.51 (s, 3H, OCH_3_), 4.63 (s, 1H, OH, D_2_O exchangeable), 4.92 (s, 1H, C4–H), 5.54 (s, 2H, NH_2_, D_2_O exchangeable), 6.92–8.44 (m, 10H, Ar–H), 9.50 (s, 1H, NH, D_2_O exchangeable), 9.92 (s, 1H, NH, D_2_O exchangeable); Anal. Calcd for C_21_H_19_N_3_O_3_ (361.39): C, 69.79; H, 5.30; N, 11.63%. Found: C, 70.04; H, 5.65; N, 11.39%.

##### 2-Amino-4–(2-chlorophenyl)-N-hydroxy-4H-benzo[h]chromene-3-carboxamidine (5c)

Yield: 61%; m.p.: > 300 °C; IR (KBr) υ (cm^−1^): 3512 (broad OH), 1610 (C = N), 1283 (C–O); MS (EI) *m/z*: 367 (M + 2, 19.6%), 365 (M^+^, 62%); ^1^H-NMR (DMSO-d_6_, 300 MHz) δ (ppm): 4.32 (s, 1H, OH, D_2_O exchangeable), 4.90 (s, 1H, C4–H), 5.23 (s, 2H, NH_2_, D_2_O exchangeable), 6.90–8.21 (m, 10H, Ar–H), 9.22 (s, 1H, NH, D_2_O exchangeable), 9.43 (s, 1H, NH, D_2_O exchangeable); Anal. Calcd for C_20_H_16_ClN_3_O_2_ (365.81): C, 65.67; H, 4.41; N, 11.49%. Found: C, 65.83; H, 4.07; N, 11.31%.

#### General procedure for the synthesis of compounds 6a–c

The appropriate 2-amio-benzo[h]chromene **1a–c** (0.01 mol) was heated under reflux in acetic anhydride (20 mL) for 20 h, cooled, neutralised with ammonia solution, poured onto ice to give precipitate which was filtered, dried, and recrystallized from ethanol to give compounds **6a–c.**

##### N-(3-Cyano-4-phenyl-4H-benzo[h]chromen-2-yl)acetamide (6a)

Yield: 65%; m.p.: > 300 °C; IR (KBr) υ (cm^−1^): 3368 (NH), 2190 (C≡N), 1655 (C = O), 1312 (C–O); MS (EI) *m/z*: 340 (M^+^, 66%); ^1^H-NMR (DMSO-d_6_, 300 MHz) δ (ppm): 2.33 (s, 3H, CH_3_), 5.21 (s, 1H, C4–H), 7.0–8.42 (m, 11H, Ar–H), 11.40 (s, 1H, NH, D_2_O exchangeable); ^13^C NMR δ: 162.88 (C = O), 161.77 (=C–NH), 159.15, 145.92, 144.15, 133.21, 128.32, 127.29, 126.98, 123.64, 119.54 (aromatic Cs), 100.39 (CN), 52.77 (C-4), 21.44 (CH_3_); Anal. Calcd for C_22_H_16_N_2_O_2_ (340.37): C, 77.63; H, 4.74; N, 8.23%. Found: C, 77.49; H, 4.53; N, 8.60%.

##### N-(3-Cyano-4–(4-methoxyphenyl)-4H-benzo[h]chromen-2-yl)acetamide (6b)

Yield: 70%; m.p.: 288–290 °C; IR (KBr) υ (cm^−1^):3391 (NH), 2230 (C≡N), 1667 (C = O), 1271 (C–O); MS (EI) *m/z*: 370 (M^+^, 42%); ^1^H-NMR (DMSO-d_6_, 300 MHz) δ (ppm): 2.47 (s, 3H, CH_3_), 3.86 (s, 3H, OCH_3_), 5.16 (s, 1H, C4–H), 6.70–7.87 (m, 10H, Ar–H), 12.47 (s, 1H, NH, D_2_O exchangeable); ^13^C NMR δ: 162.94 (C = O), 161.66 (=C–NH), 159.02 (C3), 144.17, 138.26, 133.24 (C-10b), 131.12, 129.39, 128.70, 128.25, 127.31, 127.17, 124.77, 121.21, 119.92, 114.29, 100.72 (aromatic Cs), 56.02 (C-4), 21.49 (CH_3_); Anal. Calcd for C_23_H_18_N_2_O_3_ (370.40): C, 74.58; H, 4.90; N, 7.56%. Found: C, 74.50; H, 4.58; N, 7.26%.

##### N-(4–(2-Chlorophenyl)-3-cyano-4H-benzo[h]chromen-2-yl)acetamide (6c)

Yield: 74%; m.p.: > 300 °C; IR (KBr) υ (cm^−1^): 3471 (NH), 2194 (C≡N), 1713 (C = O), 1272 (C–O); MS (EI) *m/z*: 376 (M + 2, 11.7%), 374 (M^+^, 32%); ^1^H-NMR (DMSO-d_6_, 300 MHz) δ (ppm): 2.51 (s, 3H, CH_3_), 4.89 (s, 1H, C4–H), 6.42–8.55 (m, 10H, Ar–H), 11.78 (s, 1H, NH, D_2_O exchangeable); Anal. Calcd for C_22_H_15_ClN_2_O_2_ (374.82): C, 70.50; H, 4.03; N, 7.47%. Found: C, 70.82; H, 3.87; N, 7.81%.

#### General procedure for the synthesis of compounds 7a–c

The appropriate 2-amino-benzo[h]chromene-3-carbonitrile **1a–c** (0.01 mol) was heated under reflux in acetic anhydride (20 mL) with catalytic amount of HCl (few drops) for 30 h, cooled, neutralised with ammonia solution, poured onto ice to give precipitate that was filtered, dried, and recrystallized from ethanol to give compounds **7a–c.**

##### 2-Methyl-5-phenyl-5H-benzo[h]chromeno[2,3-d]pyrimidin-4-one (7a)

Yield: 66%; m.p.: 279–281 °C; IR (KBr) υ (cm^−1^): 3432(NH), 1656 (C = O), 1588 (C = N), 1304 (C–O); MS (EI) *m/z*: 340 (M^+^, 30.4%); ^1^H-NMR (DMSO-d_6_, 300 MHz) δ (ppm): 2.32 (s, 3H, CH_3_), 5.23 (s, 1H, C5–H), 7.11–8.20 (m, 11H, Ar–H), 10.22 (s, 1H, NH, D_2_O exchangeable); Anal. Calcd for C_22_H_16_N_2_O_2_ (340.82): C, 77.63; H, 4.74; N, 8.23%. Found: C, 77.45; H, 4.98; N, 8.47%.

##### 5-(4-Methoxyphenyl)-2-methyl-5H-benzo[h]chromeno[2,3-d]pyrimidin-4-one (7b)

Yield: 70%; m.p.: > 300 °C; IR (KBr) υ (cm^−1^): 3463 (NH), 1670 (C = O), 1598 (C = N), 1298 (C–O); MS (EI) *m/z*: 370 (M^+^, 14.4%); ^1^H-NMR (DMSO-d_6_, 300 MHz) δ (ppm): 2.43 (s, 3H, CH_3_), 3.80 (s, 3H, OCH_3_), 4.78 (s, 1H, C5–H), 7.02–8.40 (m, 10H, Ar–H), 10.19 (s, 1H, NH, D_2_O exchangeable); Anal. Calcd for C_23_H_18_N_2_O_3_ (370.2): C, 74.58; H, 4.90; N, 7.56%. Found: C, 74.87; H, 4.64; N, 7.69%.

##### 5-(2-Chlorophenyl)-2-methyl-5H-benzo[h]chromeno[2,3-d]pyrimidin-4-one (7c)

Yield: 67%; m.p.: 276–278 °C; IR (KBr) υ (cm^−1^):3477 (NH), 1680 (C = O), 1593 (C = N), 1290 (C–O); MS (EI) *m/z*: 376 (M + 2, 19.2%), 374 (M^+^, 57.7%); ^1^H-NMR (DMSO-d_6_, 300 MHz) δ (ppm): 2.46 (s, 3H, CH_3_), 4.99 (s, 1H, C5–H), 6.96–8.46 (m, 10H, Ar–H), 10.83 (s, 1H, NH, D_2_O exchangeable); ^13^C NMR δ: 167.33 (C = O), 162.39 (=C–NH), 157.28, 152.15, 147.21, 131.36, 130.67, 128.84, 128.78, 128.71, 126.02, 122.94, 122.62, 114.24, 113.91 (aromatic Cs), 40.46 (C-4), 39.96 (CH_3_); Anal. Calcd for C_22_H_15_ClN_2_O_2_ (374.82): C, 70.50; H, 4.03; N, 7.47%. Found: C, 70.81; H, 3.87; N, 7.66%.

#### General procedure for the synthesis of compounds 8a-c

A mixture of equimolar amounts of the appropriate 2-amino-benzo[h]chromene-3-carbonitrile **1a–c** (0.01 mol) and carbon disulphide (0.01 mol, 0.76 g) was heated under reflux in pyridine (4 mL) for 16 h, cooled and drops of ethanol added. The separated solid was filtered, dried and recrystallized from ethanol to give compounds **8a–c.**

##### 5-Phenyl-5H-benzo[h]chromeno[2,3-d]pyrimidin-2-thiol-4-one (8a)

Yield: 73%; m.p.: 235–237 °C; IR (KBr) υ (cm^−1^): 3347 (NH), 3201 (SH),1700 (C = O), 1295 (C–O); MS (EI) *m/z*: 358 (M^+^, 39.4%); ^1^H-NMR (DMSO-d_6_, 300 MHz) δ (ppm): 1.89 (s, 1H, SH, D_2_O exchangeable), 4.71 (s, 1H, C5–H), 7.01–8.20 (m, 11 H, Ar–H), 8.61 (s, 1H, NH, D_2_O exchangeable); Anal. Calcd for C_21_H_14_N_2_O_2_S (358.48): C, 70.39; H, 3.91; N, 7.82%. Found: C, 70.61; H, 3.96; N, 7.50%.

##### 5-(4-Methoxyphenyl)-5H-benzo[h]chromeno[2,3-d]pyrimidin-2-thiol-4-one (8b)

Yield: 70%; m.p.: 223–225 °C; IR (KBr) υ (cm^−1^): 3533 (NH), 3379 (SH), 1710 (C = O), 1298 (C–O); MS (EI) *m/z*: 388 (M^+^, 55.9%); ^1^H-NMR (DMSO-d_6_, 300 MHz) δ (ppm): 1.71 (s, 1H, SH, D_2_O exchangeable), 3.80 (s, 3H, OCH_3_), 5.72 (s, 1H, C5–H), 6.90–8.22 (m, 10 H, Ar–H), 8.59 (s, 1H, NH, D_2_O exchangeable); Anal. Calcd for C_22_H_16_N_2_O_3_S (388.50): C, 68.04; H, 4.12; N, 7.22%. Found: C, 68.22; H, 3.87; N, 7.19%.

##### 5-(2-Chlorophenyl)-5H-benzo[h]chromeno[2,3-d]pyrimidin-2-thiol-4-one (8c)

Yield: 66%; m.p.: 249–251 °C; IR (KBr) υ (cm^−1^):3444 (NH), 3330 (SH), 1710 (C = O), 1308 (C–O); MS (EI) *m/z*: 395 (M + 2, 15%), 393 (M^+^, 46.2%); ^1^H-NMR (DMSO-d_6_, 300 MHz) δ (ppm): 1.89 (s, 1H, SH, D_2_O exchangeable), 5.03 (s, 1H, C5–H) 7.19–8.05 (m, 10H, Ar–H), 8.46 (s, 1H, NH, D_2_O exchangeable); Anal. Calcd for C_21_H_13_ClN_2_O_2_S (393.5): C, 64.12; H, 3.31; N, 7.12%. Found: C, 64.30; H, 3.07; N, *7.45*%.

#### General procedure for the synthesis of compounds 9a–d

A mixture of β-naphthol (0.01 mol,1.44 g), malononitrile (0.01 mol,0.66 g), and the appropriate aldehyde (0.01 mol) was heated under reflux for 24 h in ethanolic K_2_CO_3_(20%, 20 mL), cooled, poured onto ice to give precipitate that was filtered, dried, and recrystallized from ethanol to give compounds **9a–d.**

##### 3-Amino-1-phenyl-1H-benzo[f]chromene-2-carbonitrile (9a)

Yield: 62%; m.p.: 153–155 °C; IR (KBr) υ (cm^−1^): 3433, 3407 (NH_2_), 2183 (C≡N), 1342 (C–O); MS (EI) *m/z*: 298 (M^+^, 57.1%); ^1^H-NMR (DMSO-d_6_, 300 MHz) δ (ppm): 4.22 (s, 1H, C1–H), 6.94 (s, 2H, NH_2_, D_2_O exchangeable), 7.15–7.95 (m, 11 H, Ar–H); Anal. Calcd for C_20_H_14_N_2_O (298.48): C, 80.52; H, 4.73; N, 3.39%. Found: C, 80.79; H, 4.55; N, 3.01%.

##### 3-Amino-1-(2-chlorophenyl)-1H-benzo[f]chromene-2-carbonitrile (9b)

Yield: 65%; m.p.: 238–240 °C; IR (KBr) υ (cm^−1^): 3455, 3343 (NH_2_), 2177 (C≡N), 1435 (C–O); MS (EI) *m/z*: 334 (M + 2, 14.2%), 332 (M^+^, 42.7%); ^1^H-NMR (DMSO-d_6_, 300 MHz) δ (ppm): 3.30 (s, 2H, NH_2_, D_2_O exchangeable), 5.68 (s, 1H, C1–H), 6.84–7.95 (m, 10H, Ar–H); ^13^C NMR δ: 160.43(=C–NH), 147.72, 143.13, 130.61, 130.43, 130.09, 129.24, 129.04, 128.72, 127.98 (aromatic Cs), 117.33 (CN), 56.83 (C-4); Anal. Calcd for C_20_H_13_ClN_2_O (332.50): C, 72.18; H, 3.94; N, 8.42%. Found: C, 72.22; H, 3.87; N, 8.54%.

##### 3-Amino-1–(4-dimethylaminophenyl)-1H-benzo[f]chromene-2-carbonitrile (9c)

Yield: 65%; m.p.: 225–227 °C; IR (KBr) υ (cm^−1^): 3430, 3333 (NH_2_), 2212 (C≡N), 1370 (C–O); MS (EI) *m/z*: 341 (M^+^, 9.5%); ^1^H-NMR (DMSO-d_6_, 300 MHz) δ (ppm): 2.99 (s, 6H, N(CH_3_)_2_), 3.49 (s, 2H, NH_2_, D_2_O exchangeable), 4.11 (s, 1H, C1–H), 6.72–8.48 (m, 10H, Ar–H); Anal. Calcd for C_22_H_19_N_3_O (341.50): C, 77.40; H, 5.61; N, 12.31%. Found: C, 77.49; H, 5.54; N, 12.12%.

##### 3-Amino-1-(4-nitrophenyl)-1H-benzo[f]chromene-2-carbonitrile (9d)

Yield: 60%; m.p.: 196–198 °C; IR (KBr) υ (cm^−1^): 3548, 3345 (NH_2_), 2194 (C≡N), 1514 (N = O), 1344 (N–O), 1273 (C–O); MS (EI) *m/z*: 343 (M^+^, 51.4%); ^1^H-NMR (DMSO-d_6_, 300 MHz) δ (ppm): 4.42 (s, 1H, C1–H), 6.80 (s, 2H, NH_2_, D_2_O exchangeable), 7.10–8.47 (m, 10H, Ar–H); Anal. Calcd for C_20_H_13_N_3_O_3_ (343.49): C, 69.96; H, 3.82; N, 12.24%. Found: C, 69.96; H, 3.96; N, 12.37%.

#### General procedure for the synthesis of compounds 10a–d

A solution of benzo[f]chromenes **9a–d** (0.01 mol) and 4-methoxy benzaldehyde (1.36 g, 0.01 mol) was refluxed individually in DMF (15 mL) with few drops of AcOH for 20 h. The reaction mixture was allowed to cool and poured onto ice. The produced precipitate was filtered, dried and recrystallized from ethanol to give compounds **10a–d.**

##### 3-([4-Methoxyphenyl-methylene]amino)-1-phenyl-1H-benzo[f]chromene-2-carbonitrile (10a)

Yield: 62%; m.p.: 257–259 °C; IR (KBr) υ (cm^−1^): 2190 (C≡N), 1340 (C–O); MS (EI) *m/z*: 416 (M^+^, 5.8%); ^1^H-NMR (DMSO-d_6_, 300 MHz) δ (ppm): 3.78 (s, 3H, OCH_3_), 5.21 (s, 1H, C1–H), 7.10–7.93 (m, 15H, Ar–H), 9.89 (s, 1H, N = CH); Anal. Calcd for C_28_H_20_N_2_O_2_ (416.32): C, 80.75; H, 4.84; N, 6.73%. Found: C, 80.53; H, 4.80; N, 7.03%.

##### 1-(2-Chlorophenyl)-3-([4-methoxyphenyl-methylene]amino)-1H-benzo[f] chromene-2-carbonitrile (10 b)

Yield: 63%; m.p.: 260–262 °C; IR (KBr) υ (cm^−1^): 2190 (C≡N), 1290 (C–O); MS (EI) *m/z*: 352 (M + 2, 16.3%), 350 (M^+^, 49.0%); ^1^H-NMR (DMSO-d_6_, 300 MHz) δ (ppm): 3.95 (s, 3H, OCH_3_), 5.70 (s, 1H, C1–H), 6.95–7.97 (m, 14H, Ar–H), 9.91 (s, 1H, N = CH); ^13^C NMR δ: 160.36 (N = C), 147.63, 143.06, 131.24, 130.52, 130.36, 130.02, 129.16, 128.99, 128.65, 127.90, 125.52, 123.12, 120.33, 117.27 (aromatic Cs), 115.21 (CN), 56.72 (C–O), 40.60 (C-4); Anal. Calcd for C_28_H_19_ClN_2_O_2_ (450.50): C, 74.58; H, 4.25; N, 6.21%. Found: C, 74.45; H, 4.35; N, 6.51%.

##### 3-([4-Methoxyphenyl-methylene]amino)-1–(4-dimethylaminophenyl)-1H-benzo[f] chromene-2-carbonitrile (10c)

Yield: 60%; m.p.: 261–263 °C; IR (KBr) υ (cm^−1^): 2213 (C≡N),1374 (C–O); MS (EI) *m/z*: 459 (M^+^, 50.4%); ^1^H-NMR (DMSO-d_6_, 300 MHz) δ (ppm): 2.99 (s, 6H, N(CH_3_)_2_), 3.81 (s, 3H, OCH_3_), 4.40 (s, 1H, C1–H), 6.72–8.48 (m, 14H, Ar–H), 9.60 (s, 1H, N = CH); Anal. Calcd for C_30_H_25_N_3_O_2_ (459.50): C, 78.41; H, 5.48; N, 9.14%. Found: C, 78.15; H, 5.22; N, 9.35%.

##### 3-([4-Methoxyphenyl-methylene]amino)-1–(4-nitrophenyl)-1H-benzo[f]chromene -2-carbonitrile (10d)

Yield: 53%; m.p.: 253–255 °C; IR (KBr) υ (cm^−1^): 2218 (C≡N), 1513 (N = O), 1345 (N–O), 1287 (C–O); MS (EI) *m/z*: 461 (M^+^, 55.7%); ^1^H-NMR (DMSO-d_6_, 300 MHz) δ (ppm): 3.90 (s, 3H, OCH_3_), 4.32 (s, 1H, C1–H), 6.90–9.00 (m, 14H, Ar–H), 9.80 (s, 1H, N = CH); Anal. Calcd for C_28_H_19_N_3_O_4_ (461.34): C, 72.89; H, 4.12; N, 9.11%. Found: C, 72.77; H, 4.15; N, 9.11%.

#### General procedure for the synthesis of compounds 11a,b

The appropriate benzo[f]chromenes **9a,b** (0.01 mol) were refluxed individually in acetic anhydride (20 ml) for 10 h. The reaction mixture was allowed to cool, neutralised with ammonia, and poured onto ice; the produced precipitate was filtered, dried, and recrystallized from ethanol to afford compounds **11a,b**

##### N-(2-Cyano-1-phenyl-1H-benzo[f]chromen-3-yl)acetamide (11a)

Yield: 79%; m.p.: 113–115 °C; IR (KBr) υ (cm^−1^): 3455 (NH), 2177 (C≡N), 1653 (C = O), 1261 (C–O); MS (EI) *m/z*: 340 (M^+^, 50.3%); ^1^H-NMR (DMSO-d_6_, 300 MHz) δ (ppm): 2.31 (s, 3H, COCH_3_), 5.70 (s, 1H, C1–H), 7.11–7.90 (m, 11H, Ar–H), 10.60 (s, 1H, NH, D_2_O exchangeable); Anal. Calcd for C_22_H_16_N_2_O_2_ (340.34): C, 77.63; H, 4.74; N, 8.23%. Found: C, 77.66; H, 4.89; N, 8.27%.

##### N-(1–(2-Chlorophenyl)-2-cyano-1H-benzo[f]chromen-3-yl)acetamide (11b)

Yield: 81%; m.p.: 203–205 °C; IR (KBr) υ (cm^−1^): 3528 (NH), 2189 (C≡N), 1671 (C = O), 1207 (C–O); MS (EI) *m/z*: 376 (M + 2, 15.8), 374 (M^+^, 47.5%); ^1^H-NMR (DMSO-d_6_, 300 MHz) δ (ppm): 2.12 (s, 3H, COCH_3_), 5.60 (s, 1H, C1–H), 6.91–8.44 (m, 10H, Ar–H), 10.60 (s, 1H, NH, D_2_O exchangeable); ^13^C NMR δ: 170.96 (C = O), 160.12 (HN = C), 152.02, 147.94, 140.39, 130.22, 130.36, 128.92, 127.67, 123.13, 117.92, 117.37, 115.23, 92.17 (aromatic Cs), 113.50 (CN), 56.80 (C4), 25.44 (CH_3_); Anal. Calcd for C_22_H_15_ClN_2_O_2_ (374.50): C, 70.50; H, 4.03; N, 7.47%. Found: C, 70.67; H, 3.98; N, 7.32%.

### Assessment of anti-cancer activity

#### The NCI-60 human tumor cell lines screen

The operation of this screen utilises 60 different human tumour cell lines, representing leukaemia, melanoma and cancers of the lung, colon, brain, ovary, breast, prostate, and kidney cancers.

##### Selection guidelines in NCI

Structures are generally selected for screening based on their ability to add diversity to the NCI small molecule compound collection (according to the protocol of the drug evaluation branch of the NCI, Bethesda, USA^54^ (National Cancer Institute, https://dtp.cancer.gov/).

All the newly synthesised 28 compounds were selected for single-dose testing. NCI60 single-dose testing is performed in all 60 cell lines according to the reported assay (National Cancer Institute, https://dtp.cancer.gov/) (National Cancer Institute https://dtp.cancer.gov/discovery_development/nci-60/), compounds were dissolved in DMSO: glycerol (91) and are stored in a −70 °C freezer. The prepared compounds were added at single concentration of 1 0^−5^ M and the culture was incubated for 48 h. End point determinations were made with a protein binding dye, sulforhodamine B (SRB). The human tumour cell lines were derived from nine different cancer types: leukaemia, melanoma, lung, colon, central nervous system (CNS), ovarian, renal, prostate, and breast cancers.

##### Interpretation of One-dose data

The One-dose data are reported as a mean of graph of the percent growth of treated cells. The number reported for the One-dose assay is growth relative to the no-drug control, and relative to the time zero number of cells. This allows detection of both growth inhibition (values between 0 and 100) and lethality (values less than 0). For example, a value of 100 means no growth inhibition. A value of 40 would mean 60% growth inhibition. A value of 0 means no net growth over the course of the experiment. A value of −40 would mean 40% lethality. A value of −100 means all cells are dead (National Cancer Institute, https://dtp.cancer.gov/discovery_development/nci-60/).

##### NCI 60 cell Five-Dose Screen

Compounds which exhibit significant growth inhibition in the One-Dose Screen (eight or more cell lines with a growth % of 10 or less), compounds **5a** and **6a** are evaluated against the 60-cell panel at five concentration levels.

Dose–response curves for these compounds were created by plotting the percentage growth (PGs or % growth) against log10 of the corresponding concentration for every cell line. Three response parameters, GI_50_ (Median Growth Inhibitory Concentration), TGI (Total Growth Inhibitory Concentration), and LC_50_ (Median Lethal Concentration) were calculated^54^ (National Cancer Institute, https://dtp.cancer.gov/discovery_development/nci-60/) (National Cancer Institute, https://dtp.cancer.gov/)^55^ for compounds **5a** and **6a**.

The molar concentration causing 50% reduction in cell growth is termed as GI_50_ value, while the concentration leading to complete cell growth inhibition is termed as TGI value; on the other hand, LC50 value represents the concentration causing 50% loss of initial cells. Furthermore, the average sensitivity of all cell lines towards the compound is termed as the full panel mean graph midpoints (MG-MID). The ratio obtained by dividing the full panel mean graph midpoints (MG-MID) concentration by the individual subpanel MG-MID concentrations (obtained as the average sensitivity of all cell lines of a particular subpanel towards the test compound) is considered a criterion for the compound selectivity towards the corresponding cell line. Ratios greater than 6 indicate high selectivity while ratios between 3 and 6 refer to moderate selectivity whereas compounds not meeting either of these criteria were rated non-selective^56^.

#### CDK-2/cyclin A2 assay

The In vitro assay of CDK-2*/*cyclin A2 protein kinase was carried out on both the **5a** and **6a** synthesised compounds. The assay was performed using Kinase-Glo MAX luminescence kinase Assay kit (Promega #V6071) (catalogue no. 79599) (BPS Bioscience, San Diego, CA) and followed the manufacturer’s instructions. Briefly, the master mixture (6 µL of 5× Kinase assay buffer 1, 1 µL ATP, 5 µL 10× CDK substrate peptide 1and 13 µL of distilled water) was prepared and added to each well. A 5 µL of inhibitor solution was then added to each well. Then 20 µL of diluted CDK-2/cyclin A2 enzyme was added to the wells. After 45 min of incubation at 30 °C, a 50 µL of Kinase-Glo MAX reagent was added, the plate was covered with aluminium foil and it was then incubated for 15 min at room temperature. Luminescence (integration time is 1 s) was measured using the microplate reader.

#### Cell apoptosis analyzed by annexin V-FITC/PI double-staining assay

An annexin V-FITC apoptosis detection kit (BioVisionResearch Products, Mountain View, CA) (catalogue no. K101-25) was used to analyse the apoptosis using flow cytometry according to the manufacturer’s instruction. Briefly, cells were seeded (7 × 10^5^ cells/mL) and incubated with either benzocoumarin or vehicle for 48 h. Cells were collected and washed with PBS. Then, cells were resuspended in 500 µL of 1× binding buffer and 5 µL of annexin V-FITC and 5 µL of PI were added into the binding buffer. After incubation for 5 min in the dark, the log fluorescence values of annexin V-FITC and PI were shown on the *X* and *Y* axis, respectively.

#### Cell cycle phase distribution determined by flow cytometry

Cells (7 × 10^5^ cells/mL) were treated with either vehicle or benzocoumarin for 48 h. Cells were harvested and washed twice with pre-cold PBS and fixed in 66% cold ethanol overnight at 4 °C. Then, cells were rehydrated in PBS and resuspended in 500 µL PI/RNase staining buffer (abcam, ab139418) for 30 min. Propidium iodide fluorescence intensity were collected on FL2 of a flow cytometer and 488 nm laser excitation.

#### Western blotting analysis

For protein analysis of Bcl-2, caspase 3, caspase 8, FAS, CDK-2, and CyclinD1, western blot detection kit (Elabscience Biotechnology Inc., USA) (catalogue no. E-IR-R304A) was used according to the manufacturer’s instruction. Briefly, cells (1 × 10^6^ cells/mL) were treated with vehicle or benzocoumarin for 48 h. Harvested cells were washed with ice-cold PBS and lysed using ice-cold RIPA lysis buffer (catalogue no. E-BC-R327) for 30 min. Then, they were centrifuged at 12,000 rpm for 10 min at 4 °C, supernatant was collected, and the protein concentration was measured. The total protein (50 µg) was loaded and separated on 10–15% sodium dodecyl sulphate–polyacrylamide gel electrophoresis and transferred to polyvinylidene fluoride membrane (catalogue no. E-BC-R266). The membranes were soaked in Trisbuffered saline containing Tween-20 (TBST) buffer (catalogue no. E-BC-R335) containing 5% skim milk powder as a blocking buffer for 1.5 h at room temperature, and then incubated with the primary antibodies overnight at 4 °C. After incubation, the membranes were washed with TBST three times, 15 min/time. The membranes were then incubated with 1:5000 goat anti-rabbit or goat anti-mouse secondary antibodies at room temperature for 1 h on a shaker. After washing with TBST three times for 15 min/time, the immunoreactive complexes were finally visualised by excellent chemiluminescence substrate detection kit (catalogue no. E-BC-R347). The chemiluminescent signals were captured using a CCD camera-based imager (Chemi Doc imager, Biorad, USA), and the bands intensities were then measured by ImageLab (Biorad). The protein levels were first normalised to β-actin and then normalised to control.

#### Statistical analysis

Data were expressed as the mean ± SD. The statistical significance of the differences was assessed using two-way ANOVA using GraphPad Prism 8.4.2 (679) Software (San Diego, CA). A value of *p* < 0.05 was considered to be statistically significant.

### Docking studies

#### Tested compound optimisation

Docking studies using the Molecular Operating Environment (MOE 2014. 0901) software package1 were done to evaluate the activity of **5a** and **6a** compared to docked crystallised ligands (Navitoclax, PF-06873600) complexed with the (Bcl-2, CDK-2) proteins respectively, which acts as a binding site inhibitor. The thalidomide and its analogue were drawn on MOE sketcher. Structures of the co-crystallised ligands (pdb ID: 1XJ and WG1) were downloaded from the drug bank website (https://www.drugbank.ca/). After checking **5a** and **6a** and co-crystallised ligands chemical structures and the formal charges on atoms by 2D depiction, the tested compounds were subjected to energy minimisation using Force Field MMFF94x. The partial charges were automatically calculated. Each co-crystallised ligand (Navitoclax, PF-06873600) was imported with **5a** and **6a** in separate database and saved in the form of an MDB file to be used in the docking calculations as four separate processes for each protein pocket, respectively.

#### Protein active site optimisation

X-ray crystal coordinates of (pdb ID: 4LVT, 7KJS) were obtained from the Protein Data Bank (http://www.rcsb.org/). All steps for the preparation of the target protein for docking calculations were done. Hydrogen atoms were added to the system with their standard 3D geometry. Automatic correction was applied to check for any error in the atomic connections and types. Fixation of the potential of the receptor and its atoms was also performed. Site finder was used for selection of the same active site of the co-crystallised inhibitor in the enzyme structure using all default items where dummy atoms were created from the site finder of the pocket.

#### Docking of molecules into the binding site of protein

Docking compounds **5a, 6a** and the co-crystallised ligand was performed. The following methodology was applied: The prepared protein active site file was loaded, and the dock tool was initiated as general and template docking processes. The programme specifications were set up where dummy atoms act as the docking site, triangle matcher is the placement methodology, London dG is the scoring methodology, rigid receptor represents the refinement methodology, and GBVI/WSA dG is the scoring methodology for the selection of the best poses. The scoring methods were adjusted to default values. The MDB file of the three ligands in each of the four databases was loaded and general docking calculations were run automatically. The output database comprised the scores of ligand–enzyme complexes in kcal/mol. After that, the docking poses that were generated were visually evaluated, and interactions with binding pocket residues were examined. The superimposition of the binding orientations of docked molecules into the binding pocket with the top scores and showing good ligand enzyme contacts were depicted.

## Results and discussion

### Compound design

Our study aim is to design and synthesise novel benznocoumarin candidate compounds having therapeutic potential activity in AML. These compounds reduce the cell viability, induces cell cycle arrest at the G1/S phase and cell apoptosis via regulation of relative protein expression.

During normal development, cell proliferation and differentiation are inversely connected processes. All leukaemia cells have both unchecked proliferation and a lack of terminal differentiation because cell cycle exit is a prerequisite for terminal differentiation.

CDK-2, a member of the CDKs, is activated by the formation of a complex with a cyclin and is required for G1 phase progression and entry into S phase^57–59^. The role of CDK-2 is investigated in the cellular differentiation of AML cells^60^, CDK-2 was specifically degraded upon the therapeutic differentiation of AML cells, and this depletion overcame the myeloid differentiation blockade of AML cells.

Several inhibitors targeting CDK-2 have been identified over the years, and many of them are currently in different phases of drug discovery, some of the early reported CDK-2 inhibitors such as Flavopiridol^61^^,^^62^, dinaciclib, roscovitine, and staurosporine are in clinical trials, in an effort to identify potent and selective CDK-2 inhibitors have a potential activity in AML. The critical role played by CDK-2 in cell cycle regulation makes it a promising target in cancer therapy.

We inspired in our study with Flavopiridol (Alvocidib), based on the chromone alkaloid, which exhibits various mechanisms of action such as CDK inhibition, apoptosis, DNA interaction, cyclin D1 decrease, and others^63^^,^^64^.

The chromone nucleus (flavapiridol) works by mimicking the purines in the ATP structure of the coenzyme as it exhibits good kinase inhibitory activity; the effectiveness of treatment for haematological tumours has been proven^65^^,^^66^.

**Figure 2.**
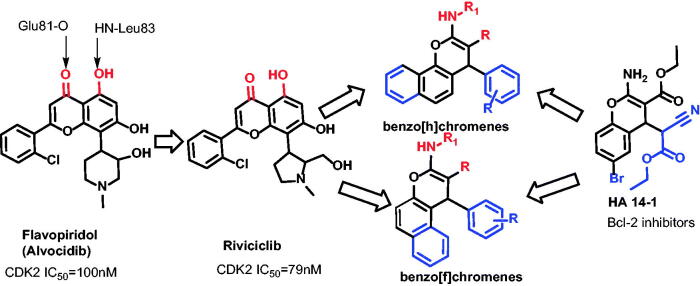
Rationalised design of the proposed new cell cycle arrest and apoptotic agents relying on the CDK-2 inhibitor and Bcl-2 inhibitor.

Riviciclib is being tested, currently, in clinical studies for human cancers such as advanced refractory neoplasms (NCT00407498), multiple myeloma (MM, NCT00882063), and relapsed or refractory mantle cell lymphoma (MCL, NCT00843050)^67^^,^^68^ (Figure 2).

Ha 14–1, which targets the Bcl-2 protein, has a crucial finding in the development of 4*H*-chromenes as anti-cancer drugs. It is a non-peptide Bcl-2 ligand, a cell permeable, low molecular weight ligand that antagonises the function of Bcl-2 and promotes apoptosis. Caspases are activated as a result of HA 14–1 attaching to the Bcl-2 surface pocket and disrupting Bax/Bcl-2 interactions. Human AML (HL-60) cells were treated with HA 14–1, which caused 100% apoptosis at a concentration of 50 μM. Mitochondria’s membrane potential is lost and caspases 9 and 3 are activated as a result of this apoptosis^44^^,^^46^. Driven by the above findings, the present study aims for developing novel cell cycle arrest and apoptosis-induction agents, based on HA14–1 and flavapiridol principal pharmacophore, with an extra goal of derivatization of chromene ring of flavapiridol as CDK-2 inhibitors and improve Bcl-2 hydrophobic cleft accommodation (Figure 2).

We focussed on two main scaffolds: 2-amino-4-aryl-4H-benzo[*h*]chromene-3-carbonitriles **1a–c** and 3-amino-1-phenyl-1H-benzo[*f*]chromene-2-carbonitriles **9a–c**, which are easily functionalised and mimic to benzochromone to get the target compounds.

Herein, two structural modification strategies have been adopted to design the newly synthesised compounds. First approach is inspiring with X-ray structure of flavapiridol cocrystallization (PDB ID: 6GUB)^69^ and its analogues X-ray structure of dechloroflavopiridol co-crystallised with CDK-2^70^, as an inhibitor with CDK-2 protein kinase produced its inhibition effect via the occupation of the chromone ring the region which fits the ATP purine ring, in addition to two hydrogen bond interactions between the flavone moiety and the key amino acids Glu81 and Leu83^71^.

We decided to pursue the development of prototypical libraries based on the chromene scaffold. The major challenge was the development of analogues with higher binding affinity. So, we spend more effort for derivatization amino cyano substitutes in order to get small library of chromene analogues maintain the interaction with the protein hot spots (key amino acids) generalised the scope between rigid structure such as substituted pyrimidone (**4**, **7a–c, 8a–c**) and flexible chain like: cyano Formamide **2**, cyano phenylformohydrazonamide **3,** amino carboximidamide **5a–c,** cyano acetamide **6a–c** (Figure 2).

The second aim of such tactic was to permit enhanced manoeuvre for these newly synthesised compounds (HA14-1 analogues) to better accommodate the Bcl-2 major hydrophobic pockets. The terminal ethyl 2-cyanoacetate chain of HA14-1 replaced with substituted phenyl groups and expand the heterocyclic scaffold from two fused ring to three fused ring to permit an enriched *π–π* stacking and other non-polar interactions with the hydrophobic residues at the Bcl-2 pocket (Figure 2).

Based on the adopted two structural modification strategies, twenty-five compounds were synthesised as well as evaluated for respective predicted anti-cancer activities.

### Chemistry

Our research had two main aims: the first was to prepare certain benzo[*h*]chromenes then carry out some modifications in their structures to study the anti-cancer activities of these derivatives and the effect of their structure modification. In order to do this, we prepared 2-amino-4-aryl-4*H*-benzo[*h*]chromene-3-carbonitriles **(1a–c)** as reported before by one-pot three-component reaction of 1-naphthol, malononitrile, different aryl aldehyde and catalytic amount of K_2_CO_3_ in ethanolic solution. Compounds **1a–c** were used for the desired structure modifications as starting materials in the next steps.

First, as revealed in Scheme A, compound **1a** was heated under reflux with formic acid for 15 h to afford its formamide derivative **2,** cyclisation to form fused pyrimidinone derivative was ruled out by examination of compound **2** spectral data. Formamide derivative **2** was then treated with phenyl hydrazine, using the reported method^72^, producing its formamidine derivative **3.** On the other hand, upon heating under reflux of **1a** in formic acid/HCl, benzo[*h*] chromeno[2,3-*d*]pyrimidin-4-one **4** was produced.

**Scheme A.**
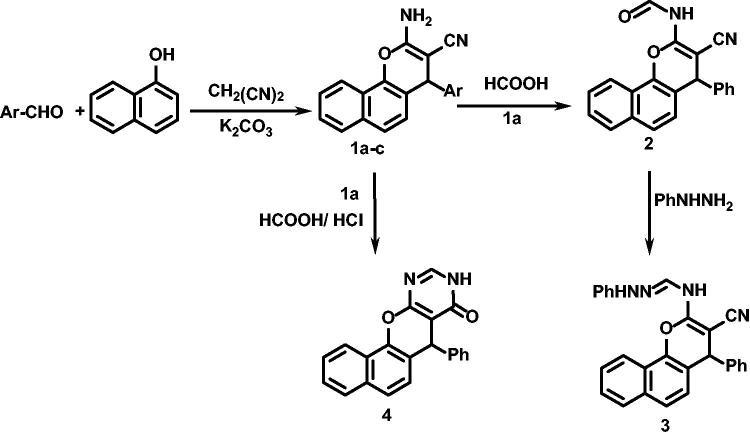
Synthesis of compounds **1a–c** to **4**.

Scheme B also revealed the use of compounds **1a–c** as starting materials for the preparation of many other derivatives. As compounds **1a–c** were heated under reflux, separately, with hydroxylamine HCl with the reported method^73^ to afford benzo[*h*]chromene derivatives **5a–c.** 2-Amino-4-aryl-4H-benzo[h]chromene-3-carbonitriles **(1a–c)** were also heated under reflux in acetic anhydride to afford acetamide derivative **6a–c,** while their reaction with acetic anhydride/HCl afforded fused benzo[*h*]chromeno[2,3-*d*]pyrimidines **7a–c**^73^^,^^74^.

**Scheme B.**
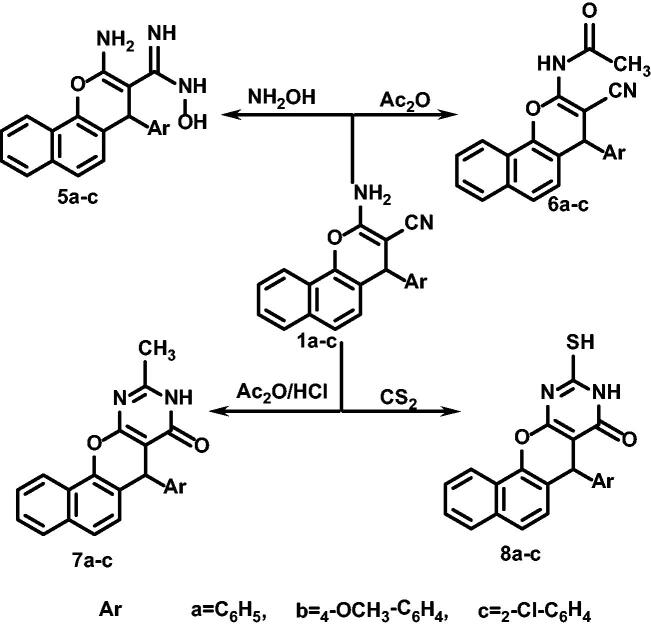
Synthesis of compounds **5a–c** to **8a–c**.

Fused benzo[h]chromeno[2,3-*d*]pyrimidines **8a–c** were prepared via the reaction of benzo[*h*]chromenes **1a–c** with CS_2_^73^^,^^74^.

Our second aim was to prepare new benzo[*f*]chromenes and then testing their anti-cancer activities, so we used the same reported method by one-pot three-component reaction of 2-naphthol, malononitrile, different aryl aldehyde and catalytic amounts of K_2_CO_3_ in ethanolic solution to produce 3-amino-1-phenyl-1H-benzo[*f*]chromene-2-carbonitriles **9a–d**, as revealed in Scheme C.

**Scheme C.**
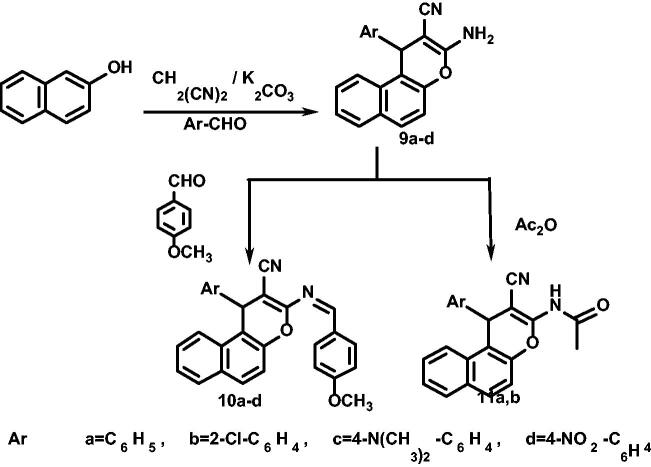
Synthesis of compounds **9a–d** to **11a,b**

Also, benzo[*f*]chromenes **9a–d** were separately reacted with 4-methoxy benzaldehyde in DMF containing few drops of AcOH to afford arylidene derivatives of benzo[f]chromene **10a–d**.

Finally, acetylation of amino group of benzo[*f*]chromenes **9a,b** was carried out by heating them, separately, under reflux with acetic anhydride to obtain acetylated benzo[*f*]chromene **11a,b**. Structures were confirmed by spectral data^73^.

### Anti-cancer testing

#### The NCI-60 human tumor cell lines screen (interpretation of One-dose data)

**Figure 3.**
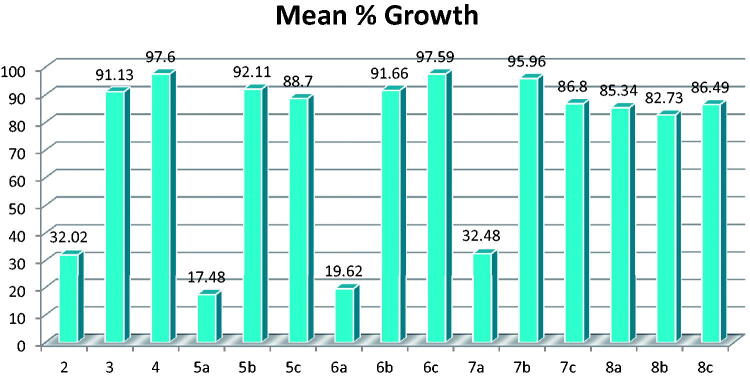
Mean % growth of compounds **2–8c**.

**Figure 4.**
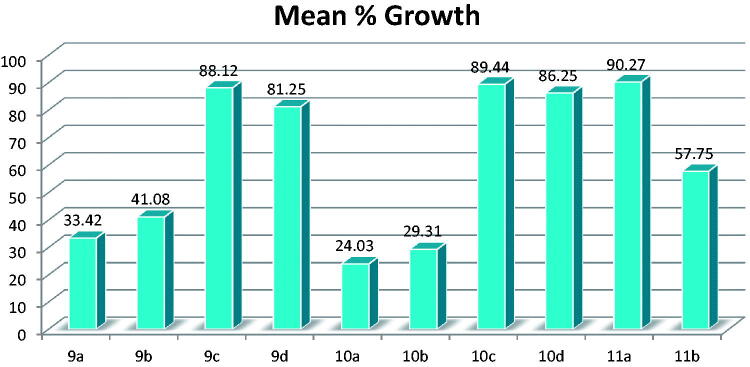
Mean % growth of compounds **9a–11b**.

All the newly synthesised compounds were selected to be tested for their anti-cancer activity via NCI60 single-dose testing; the testing results are shown in Supplementary Tables S1–S4 and Figures 3 and 4.

The results revealed that compounds **5a** and **6a** have high activity with mean values of 17.48 and 19.62, respectively.

In details, compound **5a** showed high activity against 47 of the tested cell lines with % growth ranging from −56.20% to 29.83%, moderate activity against 9 cell lines with % growth values ranging from 33.05% to 49.82%, and low activity against only 3 cell lines with % growth values ranging from 54.52% to 74.38% (Supplementary Table S1).

Compound **6a** showed high activity against 44 of the tested cell lines with % growth ranging from −42.15% to 28.76%, moderate activity against 11 cell lines with % growth values ranging from 30.76% to 49.75%, and low activity against 4 cell lines with % growth values of 50.44–62.71% (Supplementary Table S2).

The results also revealed that compounds **2**, **7a**, **9a**, **9b**, **10a**, and **10b** have moderate activity with mean values of 32.02, 32.48, 33.42, 41.08, 24.03, and 29.31, respectively.

In details, compound **2** showed high activity against 31 of the tested cell lines with % growth ranging from −33.12% to 29.35%, moderate activity against 17 cell lines with % growth values ranging from 30.32% to 43.40%, and low activity against only 8 cell lines with % growth values ranging from 54.55% to 75.43% (Supplementary Table S1).

Compound **7a** showed high activity against 30 of the tested cell lines with % growth ranging from −31.40% to 27.63%, moderate activity against 17 cell lines with % growth values ranging from 30.00% to 46.65%, and low activity against 11 cell lines with % growth values of 50.41–74.04% (Supplementary Table S2).

Compound **9a** showed high activity against 29 of the tested cell lines with % growth ranging from −21.60% to 29.53%, moderate activity against 19 cell lines with % growth values ranging from 32.48% to 49.83%, and low activity against 11 cell lines with % growth values of 50.01–73.57% (Supplementary Table S3).

Compound **9b** showed high activity against 16 of the tested cell lines with % growth ranging from −8.55% to 26.68%, moderate activity against 27 cell lines with % growth values ranging from 30.56% to 49.99%, and low activity against 16 cell lines with % growth values of 51.28–78.91% (Supplementary Table S3).

Compound **10a** showed high activity against 35 of the tested cell lines with % growth ranging from −59.17% to 29.40%, moderate activity against 19 cell lines with % growth values ranging from 32.71% to 49.72%, and low activity against 6 cell lines with % growth values of 50.66–64.68 (Supplementary Table S4).

Compound **10b** showed high activity against 32 of the tested cell lines with % growth ranging from −36.01% to 29.33%), moderate activity against 17 cell lines with % growth values ranging from 31.01% to 49.52%, and low activity against 10 cell lines with % growth values of 51.18–70.11 (Supplementary Table S4).

The results also revealed that compound **11b** has lower activity with mean value of 57.75, it showed high activity against only 5 of the tested cell lines with % growth ranging from −11.17% to 29.68%, moderate activity against 16 cell lines with % growth values ranging from 30.11% to 49.75%, and low activity against 28 cell lines with % growth values of 50.02–79.54% (Supplementary Table S4).

It was noticed that two compounds showed overall lower activity but very high anti-cancer activity with complete cell death of single cell line; namely, compound **7b** caused complete cell death of renal cell line UO-31 with growth value of −97.52% (Supplementary Table S2). And compound **9d** caused complete cell death of melanoma cell line M14 with growth value −51.31% (Supplementary Table S3). The rest of the compounds showed low to no anti-cancer activity.

The most two active compounds (**5a** and **6a**) satisfied the predetermined threshold growth inhibition criteria and further selected for the five-dose assay at 10-fold dilutions of five different concentrations and their results are revealed in Supplementary Tables S5 and S6.

#### Selectivity of compounds 5a and 6a on nine human cancer cell types

Results of the five-dose assay indicated that compounds **5a** and **6a** exhibited high activities against most of the tested cell lines as revealed from their GI_50_, TGA, and LC_50_ values; compound **5a** showed remarkable anti-cancer activity with GI_50_ values ranging between 0.03 and 28.8 μM against all the tested cell lines. With regards to the selectivity it showed high selectivity towards leukaemia and colon cancer cell lines (selectivity ratios of 7.73 and 6.4 respectively), and it is proven to be non-cytotoxic besides its activity against all the tested cell lines of leukaemia and colon cancer (LC_50_ > 100 μM). It also showed moderate selectivity towards CNS cancer, ovarian cancer, and prostate cancer cell lines (selectivity ratios of 4.43, 4.84, and 5.93 respectively), and it is also proven to be non-cytotoxic besides its activity against CNS cancer cell lines (SF-268, SF-295, SNB-19, SNB-75, and U251), ovarian cell lines (IGROV1, OVCAR-4, OVCAR-5, OVCAR-8, NCI/ADR-RES, and SK-OV-3), and prostate cancer cell lines (PC-3 and DU-145) (LC_50_ > 100 μM) and low selectivity towards non-small cell lung cancer, melanoma, renal cancer and breast cancer cell lines (selectivity ratios of 1.54, 0.25, and 2.38 respectively), as revealed in Supplementary Table S5.

Compound **6a** also showed anti-cancer activity with GI_50_ values ranging between 0.20 and 49.9 μM against all the tested cell lines. With regards to the selectivity it can be considered as broad-spectrum anti-cancer agent as it showed moderate selectivity only towards leukaemia cell lines (selectivity ratio of 3.93) and showed low selectivity towards the rest of the cell lines (< 3). It is proven to be non-cytotoxic besides its anti-leukemic activity towards all 60 tested cell lines (LC_50_ > 100 μM) except one colon cell line (COLO 205) (Supplementary Table S6).

#### CDK-2/cyclin A2 inhibitory activity

**Figure 5.**
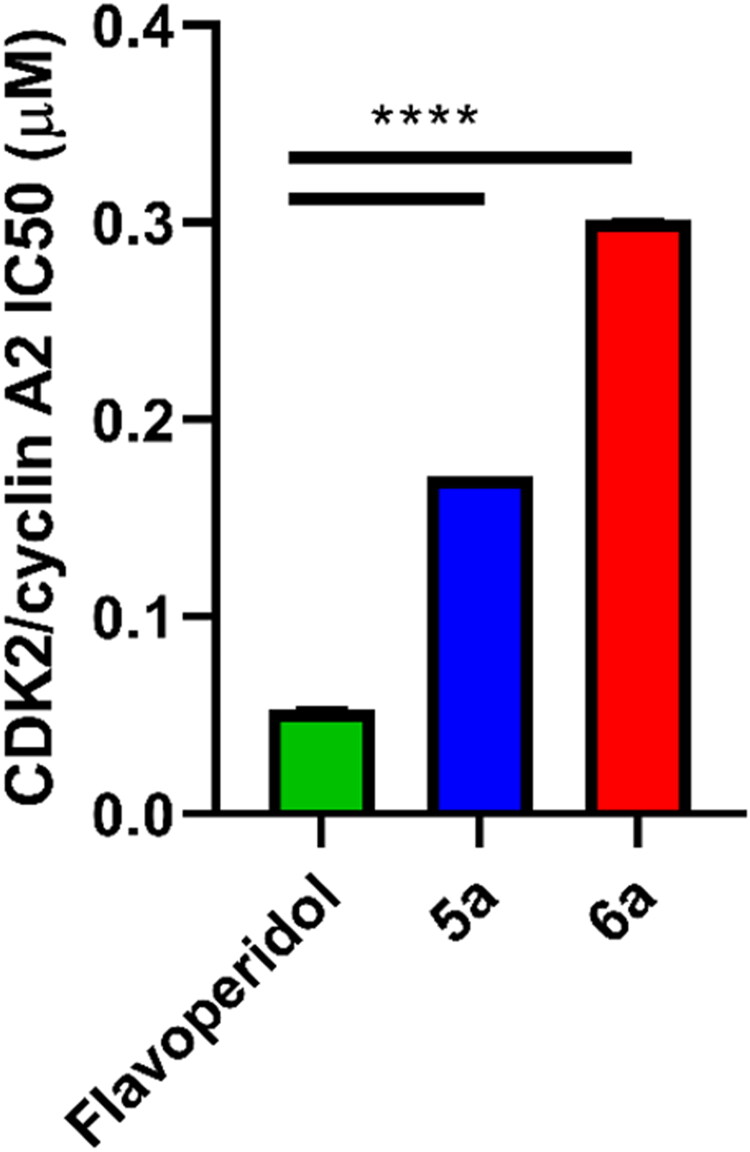
Inhibitory activity of **5a** and **6a** compounds on CDK-2/cyclin A2. *Significant from Flavoperidol at *p* < 0.0001.

Using the Promega Kinase-Glo MAX luminescence kinase assay, the in vitro CDK-2/cyclin A2 assays of both synthesised compounds **5a** and **6a** are conducted. Table 1 displays the findings of the compounds’ enzyme-inhibitory activity against CDK-2/cyclin A2. When compared to Flavoperidol, which has an IC_50_ of 0.052 ± 0.002, both tested compounds had good inhibitory effects, with values ranging from 0.171 ± 0.008 to 0.301 ± 0.013 µM. When compared to the control drug Flavoperidol, the results showed that compound **5a** had the strongest inhibitory activity, with IC_50_ values of 0.171 ± 0.008 µM (Figure 5).

**Table 1.** CDK-2/cyclin A2 inhibitory activity results of **5a** and **6a** synthesised compounds.

Compound ID	CDK-2/ cyclin A2 IC_50_ (µM) ± *SD*
Flavoperidol	0.052 ± 0.002
**5a**	0.171 ± 0.008*
**6a**	0.301 ± 0.013*

*Significant from Flavoperidol at *p* ˂ 0.0001.

#### Compounds 5a, 6a induced apoptosis in HL-60 cells

**Figure 6.**
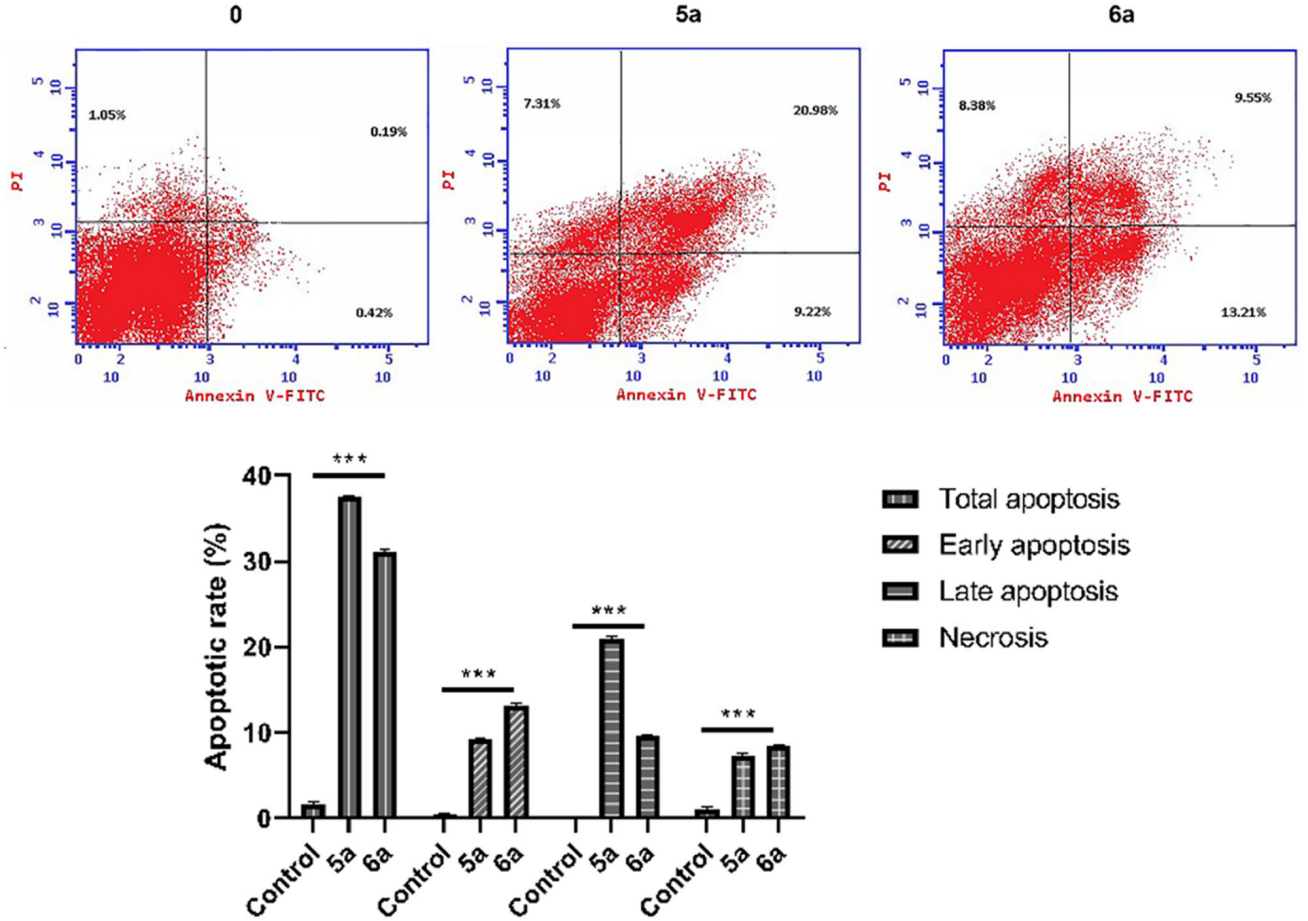
Compounds **5a**- and **6a**-induced apoptosis in HL-60 cells; effect of benzocoumarin on apoptosis. Cells were treated with compounds **5a** and **6a** (IC^50^=0.24, 0.29 respectively) for 48 h. Apoptotic cells were quantified by flow cytometry after staining with annexin V-fluorescein isothiocyanate (V-FITC)/propidium iodide (PI) (mean± *SD*, *n* = 3).

To determine the effect of **5a** and **6a** on cell death in HL-60 cells, we used flow cytometry analysis to assess the apoptotic rate of HL-60 treated with **5a** and **6a** (IC_50_=0.24, 0.29 respectively) using annexin V-FITC and PI double staining. As shown in Figure 6, the early and late apoptotic rates of HL-60 cells treated with compounds **5a** and **6a** for 48 h were significantly higher than that of the control group.

#### Compounds 5a and 6a induced both intrinsic and extrinsic apoptosis in HL-60 cells

**Figure 7.**
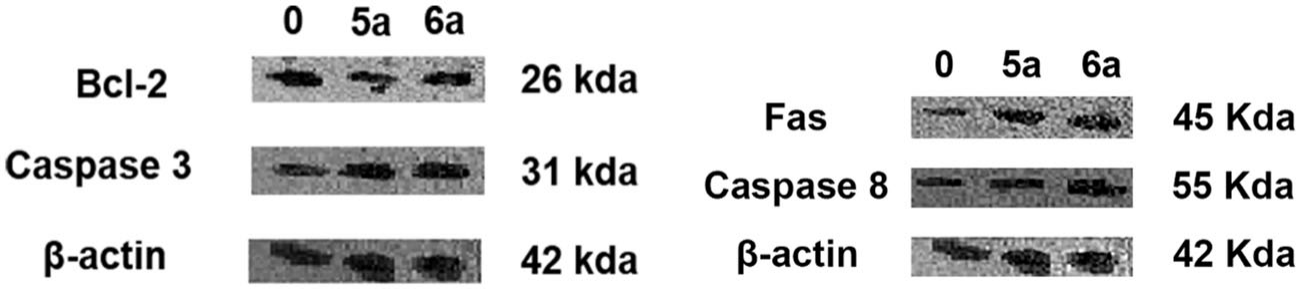
Compounds **5a**- and **6a**-induced apoptosis is mediated by both the extrinsic pathway and activation of the mitochondrial intrinsic apoptotic pathway. (A) Western blot detection of intrinsic pathway of apoptosis-associated protein expression. Cells were treated with compounds **5a** and **6a** (IC^50^=0.24, 0.29 respectively) for 48 h and then harvested. β-actin was used as an internal reference. (B) Western blot detection of extrinsic apoptotic pathway related proteins. Cells were treated with compounds **5a** and **6a** (IC^50^=0.24, 0.29 respectively) for 48 h and then harvested. β-actin was used as an internal reference. Data are presented as mean ± *SD* of three independent experiments. *p < 0.05 as compared to the control.

For further confirmation of apoptosis, we next analysed apoptosis-associated markers following for compounds **5a** and **6a** treatment (IC_50_=0.24, 0.29 respectively). Western blot analysis was utilised to detect the actual mechanism of **5a**- and **6a**-induced apoptosis where we analysed both intrinsic and extrinsic apoptosis pathways. The intrinsic apoptosis pathway is also referred to as the mitochondrial apoptosis pathway. Extrinsic apoptosis is defined as apoptosis triggered by Fas Ligands followed by caspase 8 activation, which then cleaves and activates caspase 3. We first examined the expression levels of proteins associated with mitochondrial intrinsic apoptotic pathway including Bcl-2 and caspase 3. As shown in Figure 7(A), compounds **5a** and **6a** increased the expression of caspase 3 and decreased the expression of Bcl-2 in HL-60 cells after 48 h. These results suggest that compounds **5a** and **6a** induces apoptosis through the intrinsic mitochondria pathway in HL-60 cells. The examination of the extrinsic apoptosis pathway by western blot revealed that the expression levels of Fas were increased and caspase-8 was decreased. (Figure 7(B)). These findings further support our hypothesis that both intrinsic and extrinsic apoptotic pathways are involved in compounds **5a-** and **6a**-induced apoptosis in HL-60.

#### Compounds 5a- and 6a-induced G1/S phase arrest associated with cell cycle proteins in HL-60

**Figure 8.**
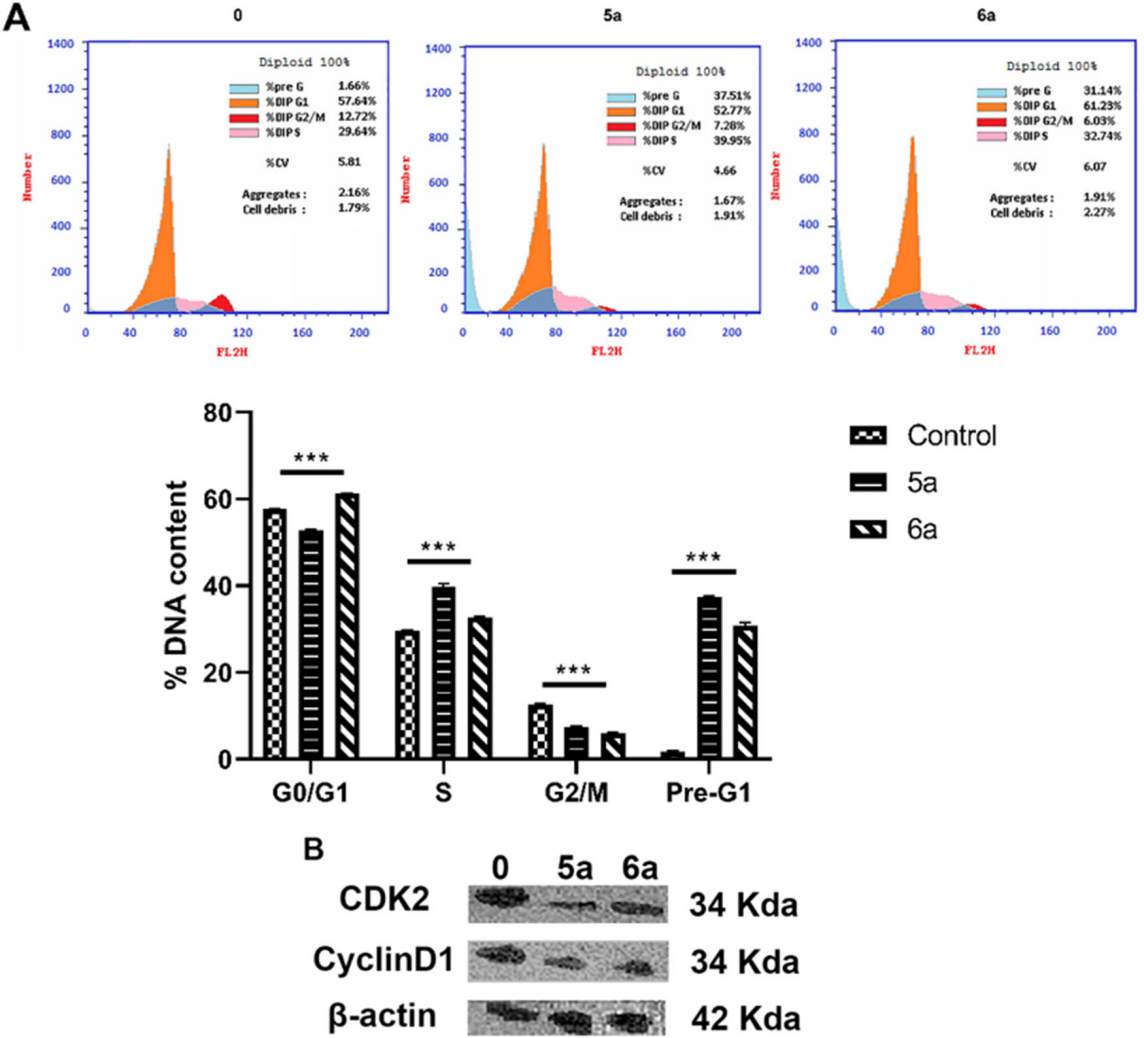
Benzocoumarin-induced cell cycle arrest in HL-60 cells. (A) Effect of benzocoumarin on cell cycle (mean± *SD*, *n* = 3). Cells were treated benzocoumarin (IC^50^=0.24, 0.29 respectively) for 48 h. The images display a representative experiment from three independent experiments. Cells were quantitatively measured by flow cytometry. ***p < 0.001 as compared to the control. (B) Western blot detection of cell-cycle-associated protein expression. Cells were treated with benzocoumarin (IC^50^=0.24, 0.29 respectively) for 48 h. β-actin was used as an internal reference.

For further determination whether the inhibitory effect of benzocoumarin on HL-60 cells is due to its induction of cell cycle arrest or else, high-resolution flow cytometry analysis of PI-stained nuclei was performed. As shown in Figure 8(A), the exposure of HL-60 cells to benzo[*h*]chromenes remarkably resulted in the accumulation of cells in G1 phase when compared with the control group, from 57.64 in the control group to 61.23 in the treated group, and the accumulation of cells in S phase increased from 29.64 in the control group to 32.74 in the treated group. Western blot analysis of the protein expression levels of the molecules involved in the G0/G1 phase arrest was also performed. CDK-2 and CyclinD1 expression levels were decreased, as shown in Figure 8(B). These findings demonstrate that benzocoumarin inhibits the growth of HL-60 cells most likely via triggering G0/G1 phase arrest.

### Discussion

Many drugs have been developed to target apoptosis in cancer cells. The disruption of mitochondrial membrane potential is linked to the early stages of apoptosis^36^. The intrinsic cell death pathway is primarily regulated by BCL-2 family proteins that reside in or are recruited to the mitochondria in response to cell death insults. The BCL-2 protein family includes both anti- and pro-apoptotic proteins. Anti-apoptotic proteins, particularly BCL-2, have been found to be overexpressed in various types of cancer, and they play important roles in tumorigenesis in various tumour models^35^. In our study, flow cytometry analysis confirmed that **5a** and **6a** induced apoptosis. Thus, we explored related protein markers of both intrinsic and extrinsic pathways. First, we found decreased expression of anti-apoptotic protein Bcl-2 and increased expression of caspase 3 indicated that **5a** and **6a** treatment activated mitochondrial pathway of apoptosis. Then, we demonstrated that Fas and caspase 8 expression levels were upregulated after **5a** and **6a** treatment. Therefore, our findings confirmed that both intrinsic and extrinsic apoptotic pathways were activated.

Cell cycle arrest is an important target for cancer therapy^36^. Our results revealed that **5a** and **6a** triggered G0/G1 and S phase arrest in HL-60. CDKs are protein kinases that must first bind to a cyclin subunit before catalysing. Throughout the cell cycle, to enter S phase all cells must activate CDKs. Different CDK family members bind to different cyclins to form Cyclin–CDK complexes, which then regulate the cell cycle^75^. The expression levels of CDK-2 and CyclinD1 were decreased following **5a** and **6a** treatment.

### Docking discussion

Molecular docking studies were carried out to predict the binding orientation of the most active benzo[*h*]chromenes analogues **5a**, **6a** with CDK-2 and Bcl-2 proteins and to explore the detailed intermolecular interactions for determining the probable binding mode studies, this was conducted using Molecular Operating Environment software (MOE, 2014.10) (DTP selection guidelines).

Proapoptotic and anti-apoptotic Bcl-2 proteins are members of the Bcl-2 family. In pro-apoptotic Bcl-2 members, the conserved Bcl-2 homology domain-3 (BH3) is responsible for mitochondrial apoptosis^76^.

BCL-2 protein interacts with BH3-only proteins via hydrophobic groove on its surface, which contains four pockets: P1, P2, P3, and P4 pockets^77^. To prevent complex formation between Bax and BCL-2 (PDB: 2XA0), which is an anti-apoptotic protein, these pockets need to be filled by either small molecules or BH3-mimetics. It is important for these molecules to interact with key residues that mediate interaction between BCL-2 and BH3-only proteins^78^^,^^79^. This binding results in releasing pro-apoptotic BH3-only proteins that can activate Bax and Bak and lead to apoptosis^80^. Several small molecules have been identified and synthesised, such as venetoclax^81^, HA14-1^82^^,^^83^.

Due to the hydrophobic nature of the binding site residues, it is difficult for inhibitors to overcome the natural substrate–Bcl-2 interactions in a competitive manner. However, important polar residues serve as anchoring sites for the stabilisation of associated ligands.

Based on the literature data, some of these crucial residues are as follows (numbering based on PDB ID: 4LVT)^84^: Asp100, Phe101, Arg104, Tyr105, Asp108, Phe109, Tyr199, Asn140, Gly142, Arg143, and Ala146.

We performed *in silico* molecular docking using the three-dimensional structures of Bcl-2, using X-ray crystal structure of Bcl-2–navitoclax (PDB ID: 4LVT). Navitoclax engages the two hydrophobic “hot spots” regions termed P2 and P4 that are known to show a high-affinity binding by pro-apoptotic peptides^85^^,^^86^.

The benzo[f]chromene **5a** and **6a** has been consistently predicted to occupy the Bcl-2 hydrophobic binding groove and the specific binding region was overlapped with those of the synthetic navitoclax.

Molecular docking protocol using the MOE was validated by re-docking the co-crystallised ligand (navitoclax) into Bcl-2 binding sites. The original poses generated from PDB were retrieved with root mean square deviation (RMSD) values in the range of, 1.15 Å and binding energy scores (S score) of −10.65 kcal/mol.

The docking study of **5a** and **6a** showed that both compounds occupied the novitoclox binding pocket of co-crystallised ligand with RMSD values 1.95 and 1.239 Å and binding energy scores of −5.006 and −5.16 kcal/mol respectively but occupies a smaller volume within the p4 hot spot.

**Figure 9.**
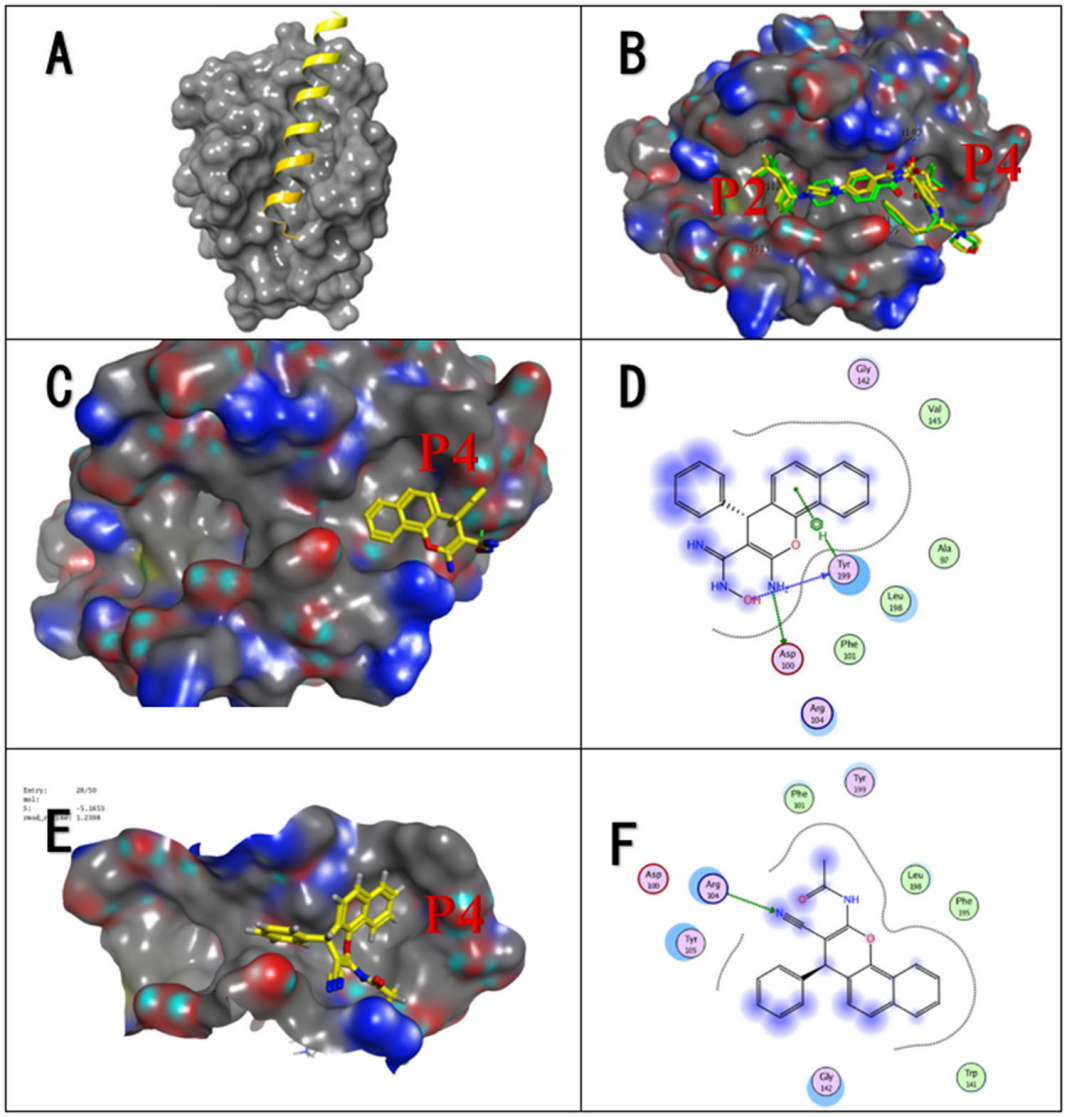
Molecular docking analysis of the compounds **5a** and **6a** in the BH3-binding groove of Bcl-2. Bcl-2 proteins are presented as gray cartoons of solid surfaces. (A) Crystal structure of Bcl-2 in complex with a Bax BH3 peptide represented by a yellow ribbon (PDB: 2XA0). (B) Validation re-docking of crystallised ligand (green) (PDB ID: 1XJ) overlay of re-docked (yellow) conformations of navitoclax in Bcl-2 (PDB: 4LVT). 3D Docking model of compounds **5a** and **6a** occupy the binding site of Bcl-2, respectively (C) and (E). Key residues at the binding pocket (D) **5a** (F) **6a**.

The Benzo[f]chromene scaffold was buried in the hydrophobic pocket of P4 Ala 97, Phe101, Gly142, Val145, Trp141, Phe195, Leu198, and Tyr199 for compounds **5a** and **6a**, each of them made a unique contact with the intra-molecular hydrogen bond formed by Asp100 and Arg104 respectively justified significant anchoring within pocket P4 (Figure 9).

In CDK-2 kinase docking studies, the standard inhibitor PF-06873600 of experimental protein crystal structure (PDB ID: 7KJS) was downloaded (PDB ID: WG1) and redocked to validate the reliability of the docking protocol. The original pose generated from PDB were retrieved with RMSD value is 0.868 Å and binding energy scores (S score) of −8.48 kcal/mol. The experimental and docked complex are typically superimposed, and its ability to reproduce all the key interactions accomplished by the co-crystallised ligand with the key amino acids (hot spots) in the active site (Glu81, Leu83, and Asp145).

According to the literature data of CDK-2, the key amino acids (hot spots) in the ATP binding pocket involved in the ATP binding are Lys33, Glu81, Leu83, and Asp145^87^.

To predict the possible binding modes of Benzo[*f*]chromene **5a** and **6a** in the ATP-binding site of CDK-2 kinase, we performed molecular docking studies and both compounds occupied the binding pocket of co-crystallised ligand with RMSD values of 1.12 and 1.44 Å and binding energy scores of −6.06 and −5.82 kcal/mol, respectively.

Comparing the CDK-2–ligands complex having PF-06873600 declared that it interacts via three hydrogen bonds with the key amino acids Glu81 and Leu83. On the other side of the carbonyl of the pyridine moiety forms a water-mediated hydrogen bond with the Asp145 backbone NH and Lys33.

According to the performed docking study, the most potent compounds **5a** and **6a** showed a common predicted binding pattern in the ATP binding site with their Benzo[*f*]chromene ring accommodated in the same region as that of the cocrystalized CDK-2 inhibitor PF-06873600 in the purine binding region.

**Figure 10.**
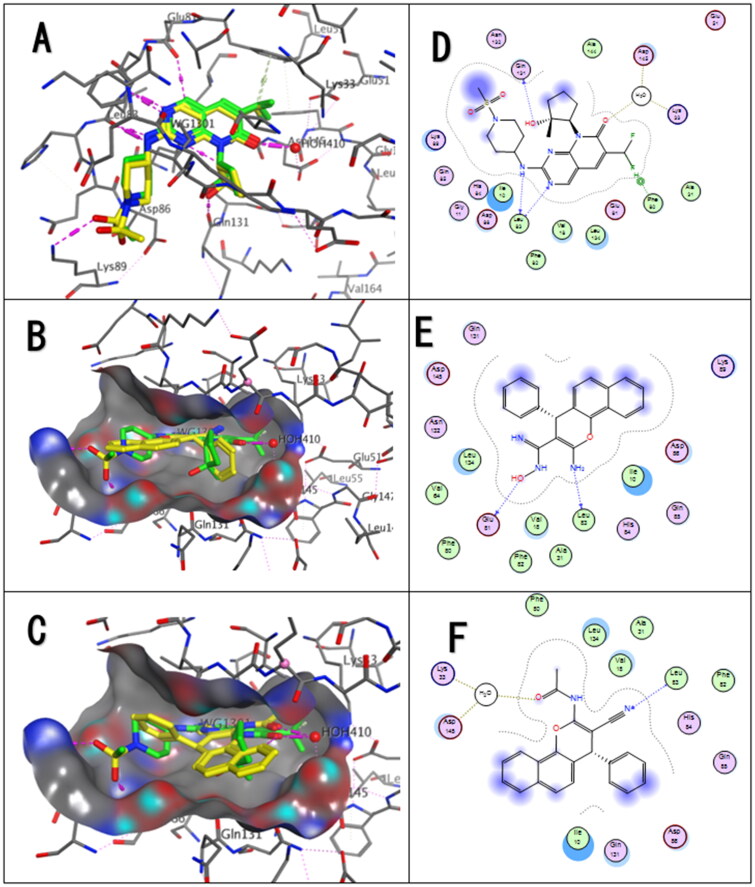
(A) Validation process for redocking WG1 into (PDB ID: 7KJS). 3D Docking model of compounds **5a** and **6a** fit into the ATP-binding site of CDK-2 kinase, respectively. (B) 3D-structure of compound **5a**; (C) 3D structure of compound **6a**. Co-crystallised ligand with green colour WG1 and the docked molecule with yellow colour. (D), (E), and (F) interaction poses of CDK2-PF-06873600, **5a,** and **6a**, respectively.

Compound **5a** binds and interacts directly with Glu81, Leu83 through hydrogen bonds through the nitrogen of amine and oxygen of carboximidamide one of the most potential modification approaches, but compound **6a** binds and interacts directly with Leu83 through hydrogen bonds with nitrogen of carbonitrile. In addition, the carbonyl of the acyl moiety forms a water-mediated hydrogen bond with the Asp145 and Lys33. Moreover, the second modification by increasing the hydrophobic moiety using extra fused ring make extra hydrophobic interaction with ILE10 (Figure 10).

## Conclusion

**Figure 11.**
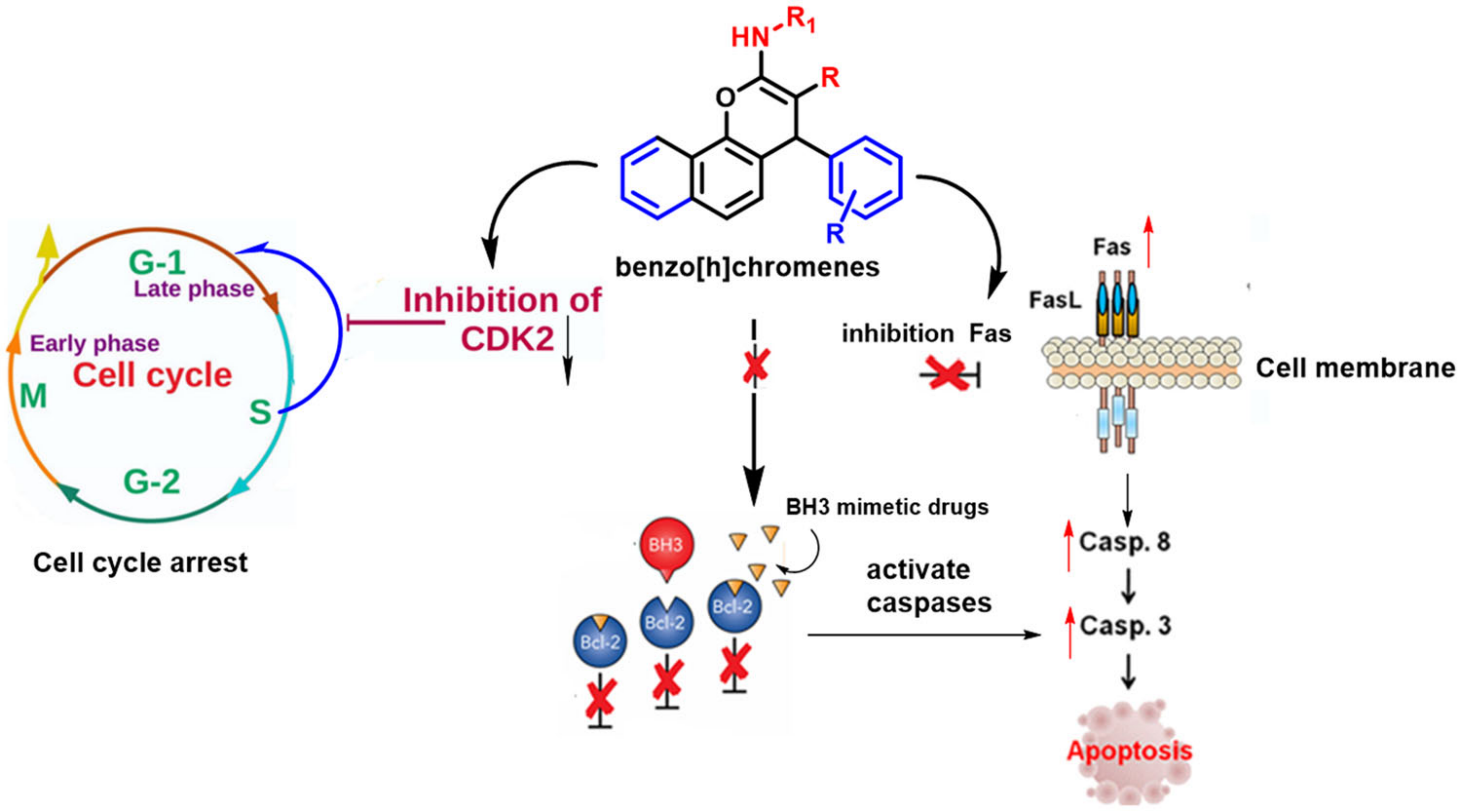
predicted mechanism of benzo[*h*]chromene.

In conclusion, we demonstrated that benzochromene derivatives **5a** and **6a** effectively promoted apoptosis and induced G0/G1 and S phase arrest in HL-60 cells. They have an inhibitory effect on HL-60 cells, which could provide a basis for future studies into **5a** and **6a**’s potential applications as promising drugs for AML therapy (Figure 11). Moreover, the docking study confirmed that the design and modification approaches for synthesis of benzochromene derivatives as CDK-2 and Bcl-2 inhibitors work perfectly, which qualifies its development as new selective inhibitors.

## Supplementary Material

Supplemental Material
