# Supplementary material for: Novel benzo chromene derivatives: design, synthesis, molecular docking, cell cycle arrest, and apoptosis induction in human acute myeloid leukemia HL-60 cells
Source: J Enzyme Inhib Med Chem. 2022 Dec 2;38(1):405–22. doi: 10.1080/14756366.2022.2151592 (PMC9721423; doi:10.1080/14756366.2022.2151592)
Supplement: Supplemental Material [file IENZ_A_2151592_SM3564.pdf]

Table 1S. Anticancer testing results for compounds **2 – 5a-c** (growth percentage against 60 cell lines).

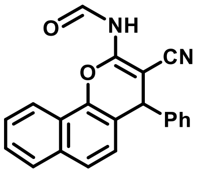

2

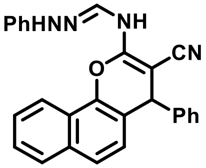

3

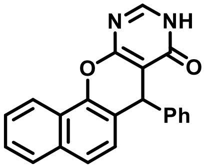

4

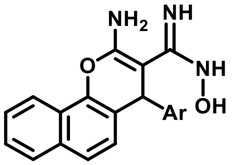

5a-c

Ar a=C_6H_5, b=$_4$-OCH_3-C_6H_4, c=$_2$-Cl-C_6H_4

**Panel/Cell Line**	**Growth Percentage**					
	**2**	**3**	**4**	**5a**	**5b**	**5c**
**<u>Leukemia:</u>**						
CCRF-CEM	NT	96.03	97.54	14.35	103.29	96.39
HL-60(TB)	18.15	120.11	104.43	**<u>-18.97</u>**	97.35	97.20
K-562	15.10	98.89	95.38	13.22	91.32	52.98
MOLT-4	39.47	108.95	86.57	27.15	94.17	95.46
RPMI-8226	19.07	96.71	90.26	19.08	95.19	94.03
SR	22.94	106.32	95.91	34.86	82.06	NT
**<u>Non-Small Cell Lung Cancer:</u>**						
A549/ATCC	36.35	98.11	99.98	21.21	102.85	100.15
EKVX	67.79	73.85	79.33	29.08	73.67	101.08
HOP-62	21.11	70.80	90.93	26.19	81.21	71.69
HOP-92	42.62	76.07	84.88	29.83	71.69	75.98
NCI-H226	62.56	72.43	90.35	1.84	59.37	80.98

Information Classification: General

NCI-H23	38.47	72.10	102.99	28.72	85.31	87.79
NCI-H322M	75.43	94.87	94.06	39.97	93.40	88.50
NCI-H460	19.94	99.02	99.90	1.37	99.28	94.91
NCI-H522	**-2.80**	77.16	92.76	5.81	84.59	45.78
**Colon Cancer:**						
COLO 205	96.45	110.55	115.28	**-5.32**	110.48	93.52
HCC-2998	63.03	94.62	106.73	11.81	106.90	98.48
HCT-116	21.13	92.61	92.98	6.57	94.48	96.42
HCT-15	30.32	91.91	102.79	5.04	95.08	98.10
HT29	94.01	104.12	116.16	6.20	106.44	129.83
KM12	30.69	91.14	100.87	11.14	100.08	90.21
SW-620	21.29	92.45	101.35	22.99	100.18	78.83
**CNS Cancer:**						
SF-268	41.26	90.24	93.71	33.05	93.76	85.65
SF-295	29.35	89.98	109.26	**-0.15**	104.46	104.64
SF-539	8.89	95.17	90.79	**-32.35**	92.65	92.04
SNB-19	30.83	97.18	98.71	24.78	96.58	95.34
SNB-75	2.51	NT	90.53	NT	55.91	75.88
U251	21.63	107.05	93.62	19.56	92.87	98.19
**Melanoma:**						
LOX IMVI	34.06	84.02	NT	22.66	88.41	92.66
MALME-3M	66.83	101.03	96.34	46.44	105.26	81.52
M14	10.23	99.02	97.36	11.69	105.89	75.70
MDA-MB-435	**-33.00**	87.58	103.47	**-56.20**	99.50	44.87

Information Classification: General

SK-MEL-2	29.01	93.25	103.36	44.68	93.98	98.08
SK-MEL-28	60.18	108.17	100.58	49.82	108.28	91.87
SK-MEL-5	**-33.12**	86.31	100.58	**-44.47**	NT	86.69
UACC-257	41.48	99.23	102.65	46.74	98.39	91.16
UACC-62	30.93	74.56	87.43	19.80	87.89	88.42
**Ovarian Cancer:**						
IGROV1	38.56	79.20	78.26	29.05	61.54	87.17
OVCAR-3	3.87	94.16	105.61	**-3.21**	102.78	94.68
OVCAR-4	64.47	90.36	98.82	47.51	96.56	102.66
OVCAR-5	88.94	103.62	94.10	29.60	98.82	102.05
OVCAR-8	23.62	91.00	96.69	17.21	93.77	89.83
NCI/ADR-RES	20.37	90.71	102.85	**-1.33**	96.56	78.34
SK-OV-3	16.31	85.02	86.08	55.25	85.23	78.42
**Renal Cancer:**						
786-0	22.03	94.48	97.09	28.97	97.62	101.92
A498	22.94	99.57	95.16	**-8.95**	NT	67.09
ACHN	43.40	89.46	95.12	28.20	87.41	92.79
CAKI-1	26.47	71.13	79.04	25.98	66.35	54.47
RXF 393	25.46	89.14	109.57	**-14.06**	96.07	99.24
SN12C	39.73	91.41	102.66	24.21	93.67	97.19
TK-10	54.55	117.70	169.97	74.38	147.58	105.65
UO-31	41.04	61.66	63.43	38.63	58.28	64.79
**Prostate Cancer:**						
PC-3	18.34	78.18	88.59	20.42	84.98	76.29

Information Classification: General

DU-145	41.35	107.89	111.92	9.00	104.85	103.66
**Breast Cancer:**						
MCF7	17.52	75.93	96.93	9.02	76.03	96.54
MDA-MB-231/ATCC	31.89	73.58	87.43	8.69	73.36	94.41
HS 578T	11.29	90.71	98.57	13.45	89.63	84.90
BT-549	25.94	88.31	92.02	28.02	91.55	96.88
T-47D	25.21	79.74	82.79	54.52	78.90	90.87
MDA-MB-468	12.00	81.84	115.82	**-1.49**	108.91	102.39
**Mean**	**32.02**	**91.13**	**97.60**	**17.48**	**92.11**	**88.70**
**Delta**	**65.14**	**29.47**	**34.17**	**73.68**	**36.20**	**43.83**
**Range**	**129.57**	**58.45**	**106.54**	**130.58**	**91.67**	**84.96**

NT: non tested

Information Classification: General

Table 2S. Anticancer testing results for compounds **6a-c-8a-c** (growth percentage against 60 cell lines).

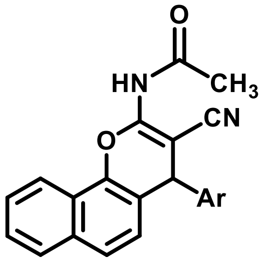

6a-c

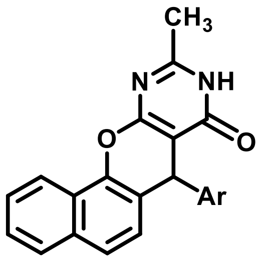

7a-c

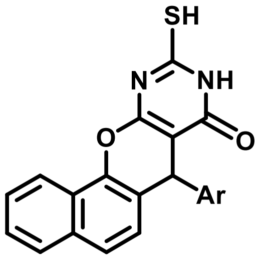

8a-c

Ar a=C_6H_5, b=$_4$-OCH_3-C_6H_4, c=$_2$-Cl-C_6H_4

Panel/Cell Line	Growth Percentage								
	**6a**	**6b**	**6c**	**7a**	**7b**	**7c**	**8a**	**8b**	**8c**
**Leukemia:**									
CCRF-CEM	NT	98.82	99.31	12.96	106.24	93.01	92.57	88.67	80.90
HL-60(TB)	0.27	113.39	99.30	**-2.80**	99.30	53.31	98.97	100.29	98.88
K-562	12.21	65.42	101.49	11.92	90.56	49.94	78.64	84.06	88.83
MOLT-4	19.81	88.85	101.25	41.04	115.24	80.34	90.04	65.95	61.38
RPMI-8226	10.99	92.61	99.83	27.11	102.49	75.51	77.79	78.18	91.70
SR	19.17	77.84	NT	19.31	NT	NT	NT	82.70	86.72
**Non-Small Cell Lung Cancer:**									
A549/ATCC	21.04	103.86	103.29	40.21	108.79	101.83	93.24	65.34	78.95
EKVX	34.56	79.17	101.19	60.52	103.23	96.15	76.36	80.40	69.53
HOP-62	21.11	65.75	86.91	20.16	89.01	76.70	85.65	85.38	83.28
HOP-92	50.44	85.10	98.13	24.68	90.96	77.74	92.84	85.56	102.54

Information Classification: General

NCI-H226	52.88	75.00	98.73	62.45	92.44	83.22	74.26	66.48	69.16
NCI-H23	28.59	92.96	100.83	50.41	92.00	87.75	80.88	84.77	91.63
NCI-H322M	45.57	96.38	88.16	59.14	100.55	86.91	87.44	67.36	84.30
NCI-H460	11.58	98.98	102.45	20.54	99.42	93.62	85.38	92.03	90.52
NCI-H522	**-4.70**	83.25	90.00	**-31.40**	77.04	81.52	72.99	83.84	77.25
**Colon Cancer:**									
COLO 205	**-23.22**	102.65	120.79	72.85	109.79	96.50	95.42	89.72	100.06
HCC-2998	**-12.22**	102.70	102.41	56.59	102.70	104.48	99.13	94.54	93.53
HCT-116	8.77	92.36	101.07	21.31	106.92	99.07	89.99	88.49	88.87
HCT-15	16.56	97.41	100.49	23.41	99.61	69.59	96.52	88.90	89.65
HT29	4.48	100.79	100.62	84.47	141.67	128.09	118.60	91.15	88.42
KM12	19.20	92.15	97.68	26.17	98.62	88.23	86.69	86.54	95.15
SW-620	23.85	89.39	96.19	20.91	97.39	92.07	95.65	98.65	86.05
**CNS Cancer:**									
SF-268	30.76	91.04	86.33	38.17	94.32	87.27	78.88	55.78	65.09
SF-295	3.77	92.64	103.90	22.77	105.35	104.20	93.59	96.67	93.62
SF-539	1.25	89.95	94.03	14.52	96.76	93.26	87.79	91.11	98.10
SNB-19	24.36	97.67	96.59	30.83	94.71	95.00	86.79	85.79	89.60
SNB-75	10.08	NT	71.35	13.14	76.83	52.53	63.19	70.71	63.34
U251	16.95	98.59	101.36	26.09	102.74	91.18	91.60	94.46	91.82
**Melanoma:**									
LOX IMVI	23.86	89.51	94.91	35.25	88.81	92.35	92.23	89.09	71.25
MALME-3M	49.20	101.15	101.73	60.91	97.01	90.87	79.18	95.57	94.31
M14	4.85	95.27	105.42	22.18	95.68	85.26	98.17	81.06	85.60

Information Classification: General

MDA-MB-435	**-42.15**	55.38	94.64	**-16.21**	42.39	83.18	94.33	90.28	96.94
SK-MEL-2	45.69	96.93	116.60	16.49	111.75	104.01	98.46	100.07	95.70
SK-MEL-28	41.46	105.08	108.53	57.73	98.72	105.16	94.84	101.83	102.93
SK-MEL-5	**-3.14**	100.94	105.00	36.83	100.78	85.98	93.19	NT	93.63
UACC-257	53.63	103.25	104.49	34.48	111.37	101.91	97.71	79.08	91.06
UACC-62	15.67	83.58	94.28	30.43	94.50	82.75	70.61	78.06	73.84
**Ovarian Cancer:**									
IGROV1	25.12	85.41	95.10	38.85	95.85	93.40	77.62	66.53	69.99
OVCAR-3	**-2.72**	98.43	97.41	0.03	107.59	85.70	92.72	71.62	97.15
OVCAR-4	62.71	93.08	98.31	64.16	112.60	85.98	66.27	62.89	79.95
OVCAR-5	28.76	98.56	95.45	87.23	113.60	97.61	92.44	94.37	87.85
OVCAR-8	12.49	99.92	102.54	34.48	101.00	85.96	90.73	91.96	90.92
NCI/ADR-RES	1.37	91.22	98.35	14.56	93.88	90.44	87.50	88.97	84.95
SK-OV-3	23.77	74.24	100.79	11.13	100.37	86.82	88.52	98.20	99.94
**Renal Cancer:**									
786-0	23.18	97.95	99.77	25.02	106.89	101.66	98.06	89.71	92.94
A498	16.28	89.85	71.89	20.56	92.53	49.95	45.45	NT	NT
ACHN	34.33	88.34	101.43	39.85	92.34	85.59	85.87	87.01	91.11
CAKI-1	24.04	67.24	64.20	30.00	73.88	67.86	56.58	60.75	66.75
RXF 393	17.95	107.91	101.34	27.63	108.39	95.23	84.31	90.94	97.76
SN12C	37.84	95.43	91.45	44.64	99.44	93.00	83.84	79.30	87.96
TK-10	45.19	118.80	107.01	74.04	116.23	107.62	99.49	93.30	97.53
UO-31	31.97	58.58	78.51	38.59	**-97.52**	63.91	53.88	60.24	58.63
**Prostate Cancer:**									

Information Classification: General

PC-3	8.32	85.34	94.95	19.69	95.99	76.33	87.07	80.97	79.43
DU-145	17.60	107.51	104.71	56.16	114.34	100.53	92.68	58.38	88.68
**Breast Cancer:**									
MCF7	14.61	90.74	91.53	11.44	96.07	60.10	68.52	63.20	73.76
MDA-MB-231/ATCC	19.01	72.77	102.03	46.65	93.58	84.16	79.01	90.55	88.97
HS 578T	18.48	91.54	93.19	22.64	96.33	88.16	86.10	85.07	88.42
BT-549	4.18	95.78	108.50	34.07	109.23	93.65	89.41	96.81	109.93
T-47D	49.75	88.99	91.89	39.13	92.19	86.13	55.30	63.97	77.06
MDA-MB-468	6.43	114.30	98.17	22.91	111.45	71.09	84.32	65.21	89.22
**Mean**	**19.62**	**91.66**	**97.59**	**32.48**	**95.96**	**86.80**	**85.34**	**82.73**	**86.49**
**Delta**	**61.77**	**36.28**	**33.39**	**63.88**	**193.48**	**36.86**	**39.89**	**26.95**	**27.86**
**Range**	**104.86**	**63.42**	**56.59**	**118.63**	**239.19**	**78.15**	**73.15**	**46.05**	**51.30**

NT: non tested

Information Classification: General

Table 3S. Anticancer testing results for compounds **9a-d** (growth percentage against 60 cell lines).

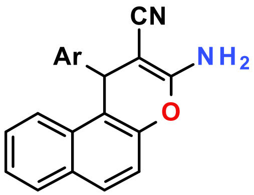

9a-d

Ar: a= C_6H_5 b=2-Cl-C_6H_4 c=4-N(CH_3)2-C_6H_4 d=4-NO_4-C_6H_4

Panel/Cell Line	Growth Percentage			
	9a	9b	9c	9d
**Leukemia:**				
CCRF-CEM	20.67	26.12	83.94	63.74
HL-60(TB)	2.02	0.77	NT	39.76
K-562	14.86	13.15	NT	77.69
MOLT-4	32.56	26.68	91.24	68.55
RPMI-8226	21.81	32.95	79.47	83.03
SR	25.06	26.06	84.29	77.26
**Non-Small Cell Lung Cancer:**				
A549/ATCC	51.01	52.98	100.87	93.73
EKVX	39.04	42.50	69.37	65.80
HOP-62	39.68	38.71	66.40	81.79
HOP-92	62.57	69.12	NT	121.32
NCI-H226	51.55	70.77	85.01	87.44
NCI-H23	43.87	61.62	86.93	78.23

Information Classification: General

NCI-H322M	62.08	74.92	87.84	89.27
NCI-H460	21.67	20.37	98.80	93.31
NCI-H522	34.72	34.40	83.76	73.54
**Colon Cancer:**				
COLO 205	32.55	39.35	99.93	102.23
HCC-2998	69.28	76.04	98.54	106.81
HCT-116	19.38	18.28	91.89	85.52
HCT-15	19.53	24.04	85.73	87.59
HT29	17.67	22.20	107.95	105.05
KM12	24.53	35.97	98.96	95.47
SW-620	27.96	30.56	95.82	100.04
**CNS Cancer:**				
SF-268	52.25	57.70	89.54	80.52
SF-295	4.96	11.71	86.85	84.64
SF-539	11.44	35.00	84.48	85.67
SNB-19	29.53	33.92	90.06	86.82
SNB-75	**-16.30**	**-2.04**	51.28	61.68
U251	32.48	37.20	92.79	94.15
**Melanoma:**				
LOX IMVI	42.35	46.19	99.29	51.35
MALME-3M	13.62	13.40	98.88	89.70
M14	**-21.60**	**-8.55**	97.87	**-51.31**
MDA-MB-435	49.70	45.91	103.21	93.92
SK-MEL-2	54.62	61.13	105.90	99.06

SK-MEL-28	25.71	42.74	95.07	86.19
SK-MEL-5	61.67	66.72	100.97	89.27
UACC-257	18.64	24.18	81.28	77.34
UACC-62	42.35	46.19	99.29	51.35
**Ovarian Cancer:**				
IGROV1	25.45	34.05	57.06	50.59
OVCAR-3	18.38	31.23	86.28	79.36
OVCAR-4	73.57	78.91	79.38	69.27
OVCAR-5	55.80	77.18	100.82	107.53
OVCAR-8	49.83	61.81	91.32	85.65
NCI/ADR-RES	8.41	9.51	95.29	94.57
SK-OV-3	47.19	55.88	82.08	83.38
**Renal Cancer:**				
786-0	35.49	42.39	96.89	88.12
A498	21.52	40.08	83.16	84.82
ACHN	37.31	49.94	80.38	76.84
CAKI-1	23.34	34.55	56.76	52.83
RXF 393	23.29	58.72	96.32	94.68
SN12C	42.23	49.99	89.76	90.43
TK-10	121.42	117.90	145.74	156.27
UO-31	44.89	48.90	55.01	51.14
**Prostate Cancer:**				
PC-3	40.59	46.14	99.26	69.83
DU-145	51.18	73.31	100.60	103.89

Information Classification: General

**<u>Breast Cancer:</u>**				
MCF7	14.84	18.07	76.33	66.01
MDA-MB-231/ATCC	47.03	51.28	58.42	57.81
HS 578T	23.80	40.36	88.02	90.23
BT-549	19.80	31.66	77.49	91.71
T-47D	40.82	37.96	84.73	82.47
MDA-MB-468	12.65	31.10	94.39	66.73
**<u>Mean</u>**	**33.42**	**41.08**	**88.12**	**81.25**
**<u>Delta</u>**	**55.02**	**49.63**	**36.84**	**132.56**
**<u>Range</u>**	**143.02**	**126.45**	**94.46**	**207.58**

NT: non tested

Information Classification: General

Table 4S. Anticancer testing results for compounds **10a-d-11a,b** (growth percentage against 60 cell lines).

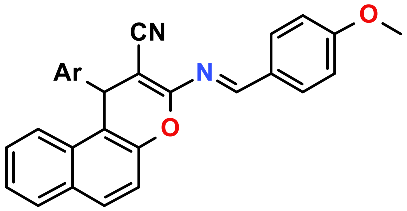

10a-d

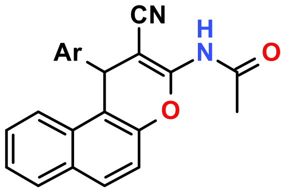

11a-b

Ar: a= C_6H_5 b=2-Cl-C_6H_4 c=4-N(CH_3)2-C_6H_4 d=4-NO_4-C_6H_4

**Panel/Cell Line**	**Growth Percentage**					
	**10a**	**10b**	**10c**	**10d**	**11a**	**11b**
**Leukemia:**						
CCRF-CEM	15.17	16.92	96.77	79.58	92.17	60.81
HL-60(TB)	**-18.13**	**-18.60**	87.19	83.77	97.38	29.68
K-562	8.18	11.13	90.98	83.41	81.57	19.43
MOLT-4	27.35	21.76	94.31	70.75	82.53	50.17
RPMI-8226	**-2.95**	17.38	78.96	90.88	90.92	55.51
SR	17.49	17.67	86.95	82.19	93.43	37.10
**Non-Small Cell Lung Cancer:**						
A549/ATCC	41.87	44.92	104.63	91.74	111.99	67.08
EKVX	32.71	33.90	73.17	72.19	69.58	63.51
HOP-62	35.48	49.52	71.65	80.22	81.70	59.20
HOP-92	53.93	44.78	65.43	85.12	89.14	72.50
NCI-H226	33.81	35.97	82.38	85.69	75.64	83.53

Information Classification: General

NCI-H23	36.93	53.46	90.57	81.70	83.29	76.25
NCI-H322M	40.91	62.68	91.76	91.45	92.27	86.83
NCI-H460	15.48	16.80	95.91	96.08	94.35	39.13
NCI-H522	2.30	3.61	87.31	76.76	72.43	39.22
**Colon Cancer:**						
COLO 205	0.29	31.32	115.69	107.01	110.48	79.54
HCC-2998	48.76	65.41	105.06	105.56	99.25	85.44
HCT-116	24.75	21.82	92.69	93.46	91.11	47.50
HCT-15	22.49	16.68	87.34	91.57	87.74	32.00
HT29	5.31	10.28	113.08	103.16	105.76	51.62
KM12	29.05	24.95	98.19	95.70	88.53	51.21
SW-620	29.40	23.68	91.89	97.17	90.77	42.96
**CNS Cancer:**						
SF-268	50.66	45.93	89.88	81.60	88.60	68.53
SF-295	10.89	21.81	93.59	84.91	92.36	25.70
SF-539	6.89	7.29	89.43	86.49	93.76	58.45
SNB-19	22.89	26.80	98.42	94.55	88.23	58.06
SNB-75	**-39.37**	**-30.11**	41.13	51.75	88.22	30.11
U251	24.08	25.59	98.10	95.57	98.82	69.34
**Melanoma:**						
LOX IMVI	28.46	26.70	64.02	80.55	NT	NT
MALME-3M	47.50	44.49	101.99	82.17	91.35	48.41
M14	11.81	15.56	102.91	86.84	86.63	31.10
MDA-MB-435	**-59.17**	**-36.01**	97.87	91.41	72.40	**-11.17**

Information Classification: General

SK-MEL-2	49.72	51.18	117.01	101.56	92.14	59.20
SK-MEL-28	64.68	70.11	112.07	101.37	94.27	66.44
SK-MEL-5	20.79	31.28	92.04	90.89	87.29	56.72
UACC-257	61.76	62.56	106.03	100.70	94.18	76.65
UACC-62	37.29	36.28	88.37	76.91	67.21	36.57
**Ovarian Cancer:**						
IGROV1	23.50	32.94	56.45	49.87	54.28	46.05
OVCAR-3	**-11.04**	12.46	79.71	81.76	86.27	41.22
OVCAR-4	52.29	52.76	92.84	77.63	94.25	88.79
OVCAR-5	48.78	62.27	106.66	101.22	99.19	86.25
OVCAR-8	37.62	47.05	98.20	88.36	92.06	81.99
NCI/ADR-RES	10.28	22.06	93.90	97.69	87.43	33.80
SK-OV-3	45.33	52.42	89.30	81.46	96.29	85.00
**Renal Cancer:**						
786-0	29.24	19.80	102.88	93.97	93.74	82.67
A498	**-6.08**	**-4.91**	87.75	92.12	105.37	56.16
ACHN	35.26	31.01	89.18	79.39	89.93	65.01
CAKI-1	13.87	20.29	54.16	46.32	70.47	37.16
RXF 393	**-8.72**	26.52	82.03	91.30	87.41	77.23
SN12C	47.97	39.64	81.32	87.53	90.45	70.75
TK-10	64.42	81.98	141.32	144.18	177.35	140.94
UO-31	37.47	42.98	52.78	50.13	53.25	49.75
**Prostate Cancer:**						
PC-3	23.30	23.76	75.51	73.49	80.58	60.57

Information Classification: General

DU-145	24.57	55.65	107.76	101.81	108.43	89.11
**Breast Cancer:**						
MCF7	15.39	11.10	72.51	66.98	97.41	42.13
MDA-MB-231/ATCC	33.42	38.04	63.62	66.71	77.55	66.64
HS 578T	17.46	15.89	87.32	84.38	93.50	74.88
BT-549	35.66	29.33	77.26	104.42	83.35	50.02
T-47D	36.27	36.67	83.07	77.10	102.34	62.53
MDA-MB-468	**-3.92**	3.32	96.06	84.91	87.49	13.95
**Mean**	**24.03**	**29.31**	**89.44**	**86.25**	**90.27**	**57.75**
**Delta**	**83.20**	**65.32**	**48.31**	**39.93**	**37.02**	**68.92**
**Range**	**123.85**	**117.99**	**100.19**	**97.86**	**124.10**	**152.11**

NT: non tested

Information Classification: General

Table 5S. Anticancer testing results for compounds **5a 5 doses assay** (growth percentage against 60 cell lines).

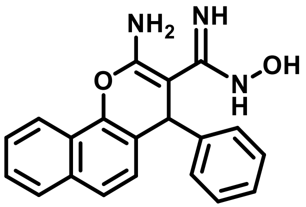

5a

**Panel**	**Cell line**	**GI_{50}**			**TGI**	**LC_{50}**
		Conc. Per cell line Subpanel MID[b] Selectivity ratio				
**Leukemia**	CCRF-CEM	0.38	0.265	7.73	39.5	>100
	HL-60(TB)	0.24			0.67	>100
	K-562	0.06			>100	>100
	MOLT-4	0.43			>100	>100
	RPMI-8226	0.38			45	>100
	SR	0.1			>100	>100
**Non-small cell lung cancer**	A549/ATCC	0.42	1.328	1.54	>100	>100
	EKVX	0.82			>100	>100
	HOP-62	0.38			>100	>100
	HOP-92	0.82			>100	>100
	NCI-H226	8.01			77.1	>100
	NCI-H23	0.47			>100	>100
	NCI-H322M	0.66			>100	>100
	NCI-H460	0.31			21.1	>100
	NCI-H522	0.06			17.8	>100

Information Classification: General

**Colon cancer**	COLO 205	0.46	0.32	6.4	7.26	>100
	HCC-2998	0.37			21.3	>100
	HCT-116	0.41			65.8	>100
	HCT-15	0.13			19.8	>100
	HT29	0.48			>100	>100
	KM12	0.22			94.6	>100
	SW-620	0.17			>100	>100
**CNS cancer**	SF-268	1.32	0.463	4.43	>100	>100
	SF-295	0.3			16.1	>100
	SF-539	0.21			0.58	61.5
	SNB-19	0.49			>100	>100
	SNB-75	0.08			>100	>100
	U251	0.38			18.7	>100
**Melanoma**	LOX IMVI	0.54	8.363	0.25	15.8	91
	MALME-3M	15.6			>100	>100
	M14	0.13			29.8	>100
	MDA-MB-435	0.03				>100
	SK-MEL-2	28.7			>100	>100
	SK-MEL-28	11.8			42.3	>100
	SK-MEL-5	0.32			8.00	57.5
	UACC-257	18.1			>100	>100
	UACC-62	0.05			19.7	96.6
**Ovarian cancer**	IGROV1	0.33	0.423	4.84	>100	>100
	OVCAR-3	0.18			0.57	43.6

Information Classification: General

	OVCAR-4	0.76			>100	>100
	OVCAR-5	0.61			>100	>100
	OVCAR-8	0.40			86.3	>100
	NCI/ADR-RES	0.06			1.65	>100
	SK-OV-3	0.62			>100	>100
**Renal cancer**	786-0	0.56	2.389	0.86	>100	>100
	A498	0.11			0.60	>100
	ACHN	0.37			36.1	>100
	CAKI-1	0.1			40.3	>100
	RXF 393	0.2				>100
	SN12C	0.53			31.1	>100
	TK-10	16.8			>100	>100
	UO-31	0.44			>100	>100
**Prostate cancer**	PC-3	0.23	0.345	5.93	>100	>100
	DU-145	0.46			>100	>100
**Breast cancer**	MCF7	0.21	0.862	2.38	>100	>100
	MDA-MB-231/ATCC	0.63			17.9	>100
	HS 578T	0.23			>100	>100
	BT-549	1.25			92.6	>100
	T-47D				>100	>100
	MDA-MB-468	1.99			>100	>100
**Mid[a]**	2.049					

Molar concentrations producing 50% growth inhibition (GI_{50}), molar concentrations producing total growth inhibition (TGI), molar concentrations producing 50% cellular death (LC_{50}).

Information Classification: General

Table 6S. Anticancer testing results for compounds **6a 5 doses assay** (growth percentage against 60 cell lines).

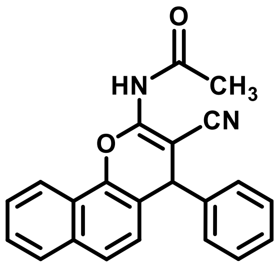

6a

**Panel**	**Cell line**	**GI_{50}** Conc. Per cell line	Subpanel MID[b]	Selectivity ratio	**TGI**	**LC_{50}**
**Leukemia**	CCRF-CEM	0.39	0.395	3.93	>100	>100
	HL-60(TB)	0.29			1.11	>100
	K-562	0.32			>100	>100
	MOLT-4	0.49			>100	>100
	RPMI-8226	0.41			>100	>100
	SR	0.47			>100	>100
**Non-small cell lung cancer**	A549/ATCC	0.98	6.241	0.25	>100	>100
	EKVX	49.9			>100	>100
	HOP-62	0.69			>100	>100
	HOP-92	0.61			>100	>100
	NCI-H226	1.85			>100	>100
	NCI-H23	0.73			>100	>100
	NCI-H322M	0.82			>100	>100
	NCI-H460	0.37			15.5	>100

Information Classification: General

	NCI-H522	0.22			0.77	>100
**Colon cancer**	COLO 205	0.61	0.6	2.59	2.49	20.6
	HCC-2998	1.43			>100	>100
	HCT-116	0.43			>100	>100
	HCT-15	0.51			>100	>100
	HT29	0.38				>100
	KM12	0.43			>100	>100
	SW-620	0.41			>100	>100
**CNS cancer**	SF-268	0.93	0.52	2.98	>100	>100
	SF-295	0.33			>100	>100
	SF-539	0.35			1.44	>100
	SNB-19	0.78			>100	>100
	SNB-75	0.24				>100
	U251	0.49			>100	>100
**Melanoma**	LOX IMVI	0.60	0.58	2.68	>100	>100
	MALME-3M	0.67			>100	>100
	M14	0.56			>100	>100
	MDA-MB-435	0.23			0.57	>100
	SK-MEL-2	0.65			>100	>100
	SK-MEL-28	0.86			>100	>100
	SK-MEL-5	0.36			1.84	>100
	UACC-257				>100	>100
	UACC-62	0.71			>100	>100
**Ovarian cancer**	IGROV1	0.53	0.732	2.12	>100	>100

Information Classification: General

	OVCAR-3	0.34			2.30	>100
	OVCAR-5	2.10			>100	>100
	OVCAR-8	0.63			>100	>100
	NCI/ADR-RES	0.30			1.26	>100
	SK-OV-3	0.49			>100	>100
**Renal cancer**	786-0	0.49	1.139	1.36	>100	>100
	A498	0.20			0.73	>100
	ACHN	0.55			>100	>100
	CAKI-1	0.34			>100	>100
	RXF 393	0.27				>100
	SN12C	0.70			>100	>100
**Prostate cancer**	PC-3	0.46	0.595	2.61	>100	>100
	DU-145	0.73			41.3	>100
**Breast cancer**	MCF7	0.47	0.662	2.35	>100	>100
	MDA-MB-231/ATCC	1.25			12.3	>100
	HS 578T	0.36			>100	>100
	BT-549	0.99			6.02	>100
	T-47D				>100	>100
	MDA-MB-468	0.24			0.71	>100
**Mid**[a]	1.553					

Molar concentrations producing 50% growth inhibition (GI_{50}), molar concentrations producing total growth inhibition (TGI), molar concentrations producing 50% cellular death (LC_{50}).

Information Classification: General

Spectral Data

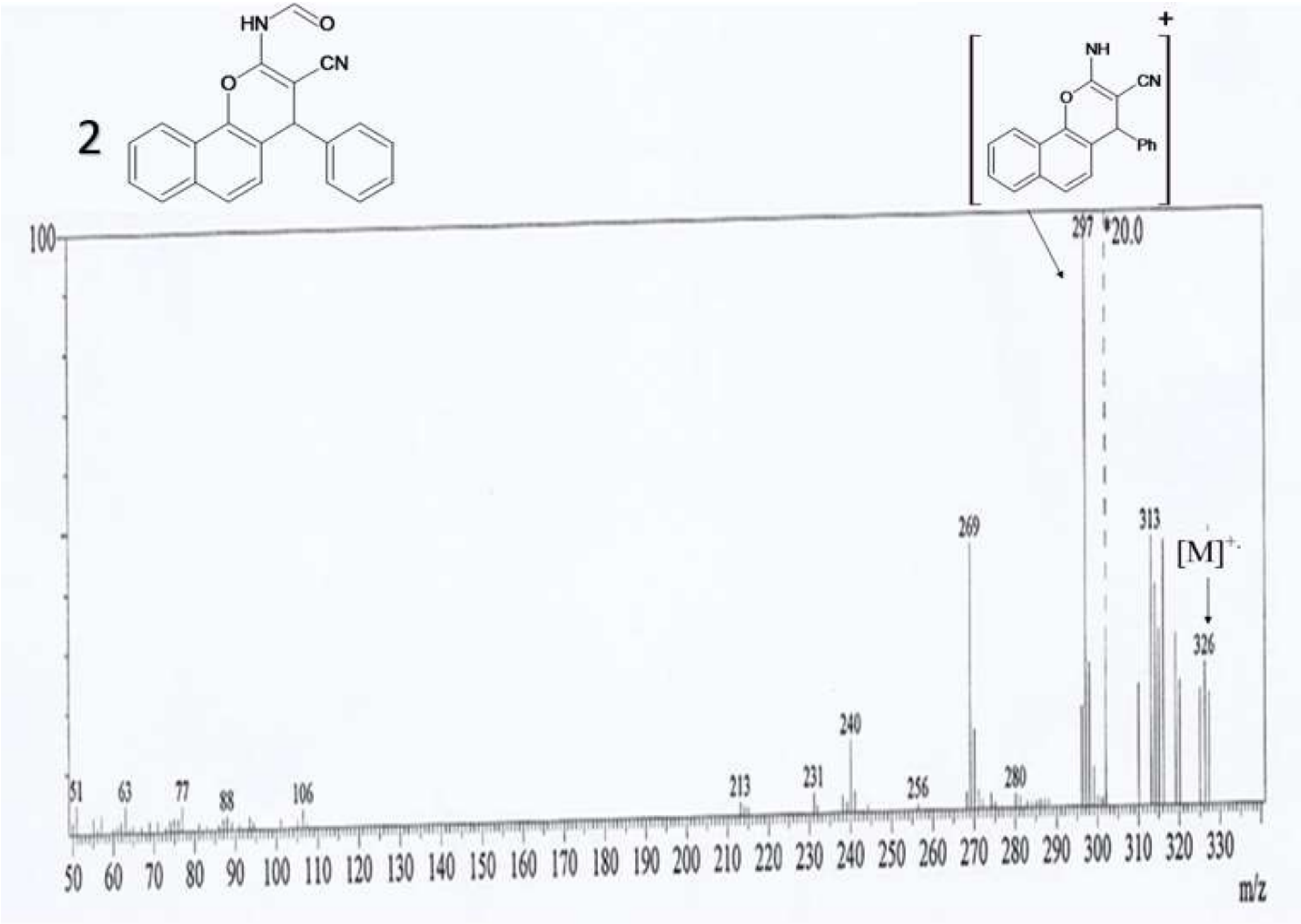

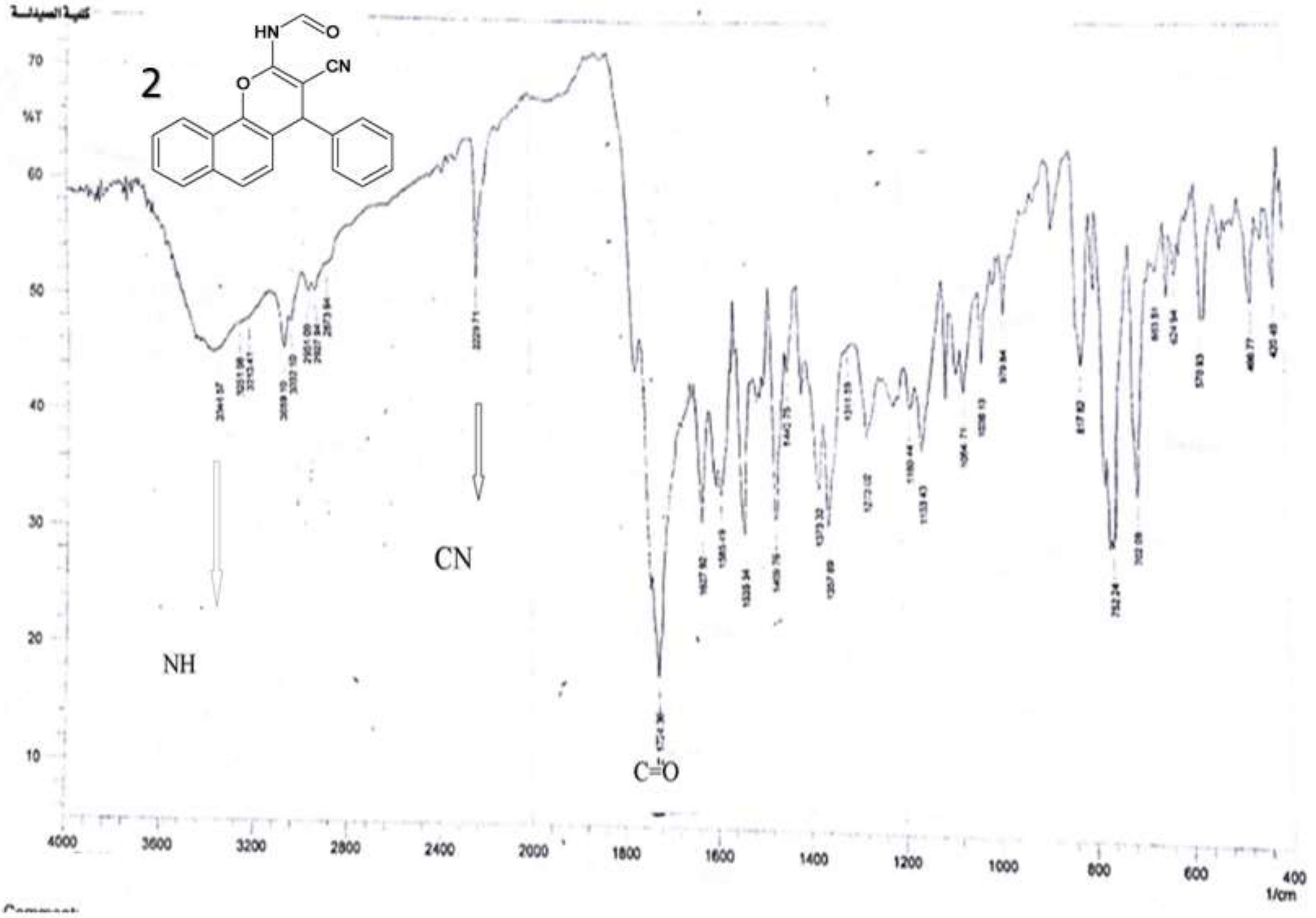

Information Classification: General

3

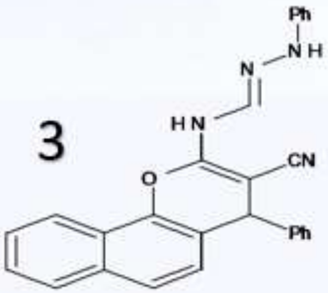
Ph
NH
N
HN
CN
O
Ph

IIIa

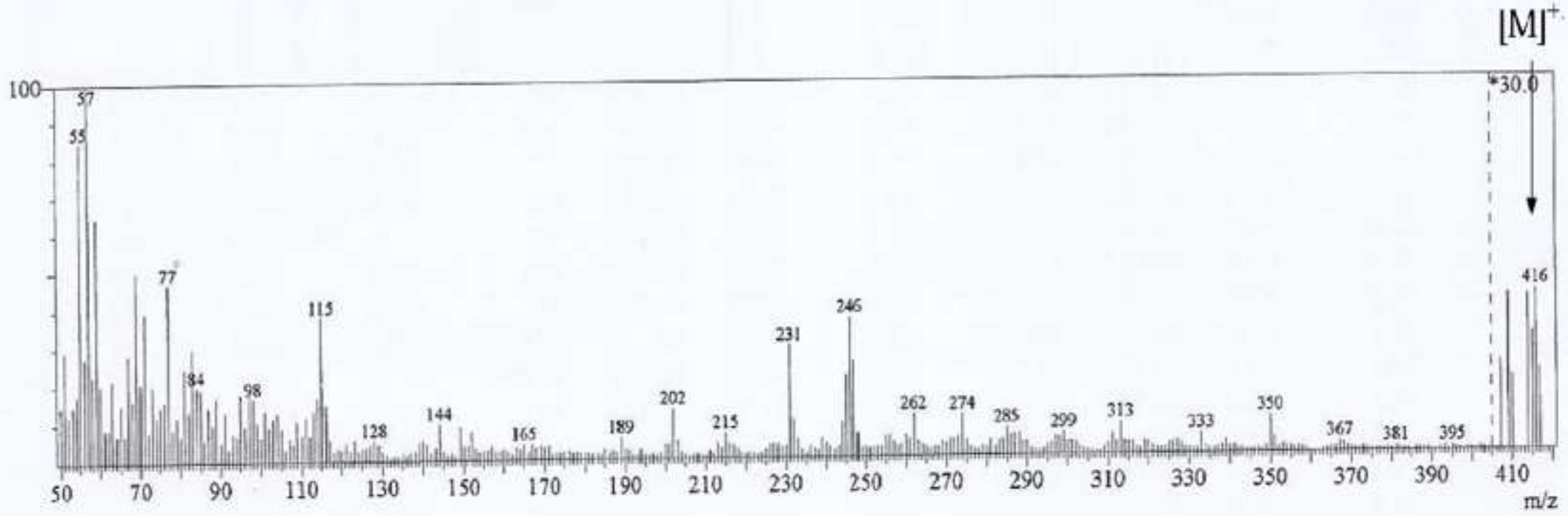
[M]+·
100
*30.0
57
55
77
84
98
115
128
144
165
189
202
215
231
246
262
274
285
299
313
333
350
367
381
395
416
50
70
90
110
130
150
170
190
210
230
250
270
290
310
330
350
370
390
410
m/z

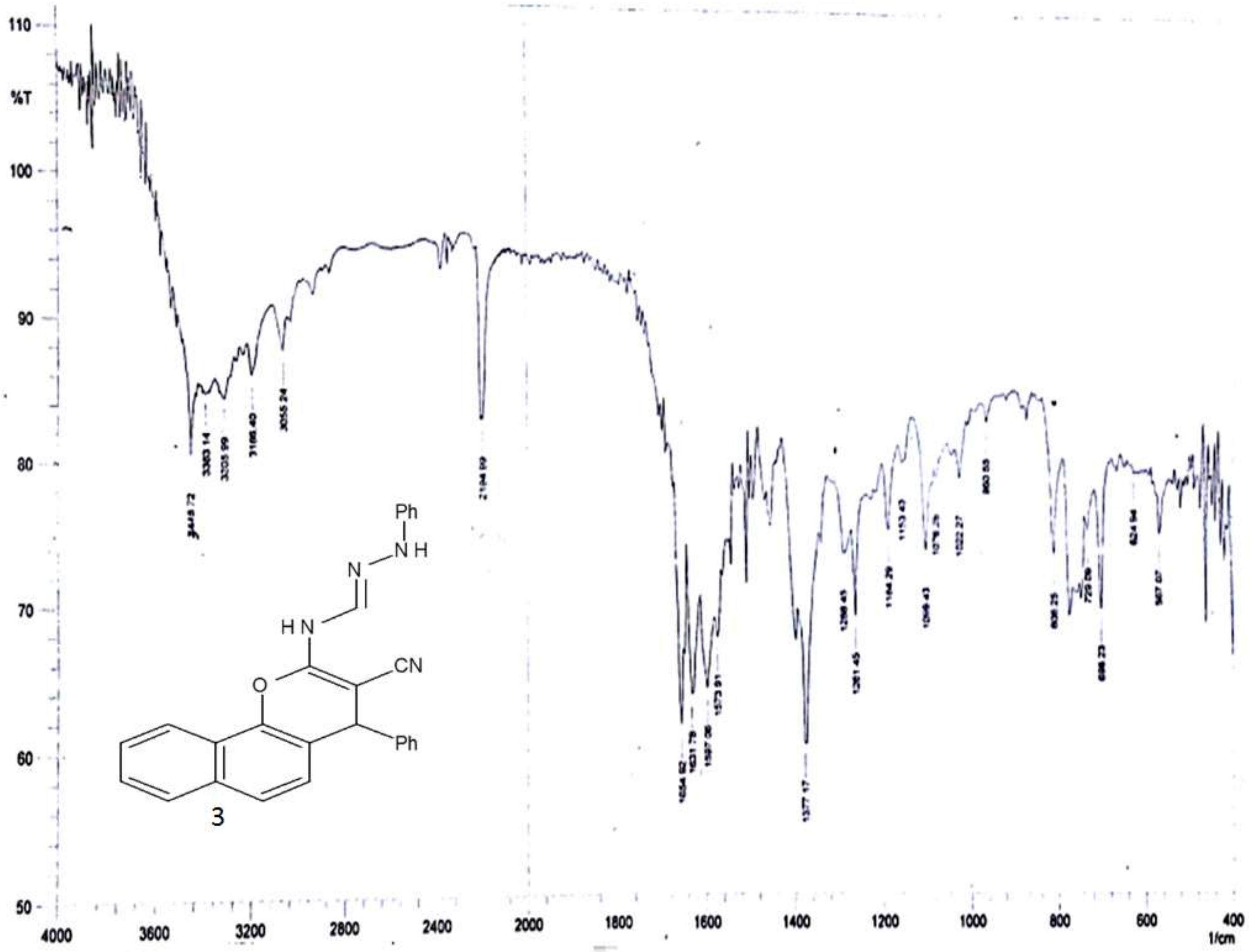

Information Classification: General

4

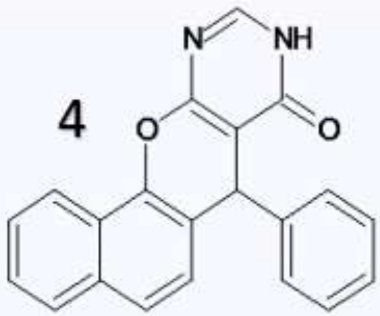

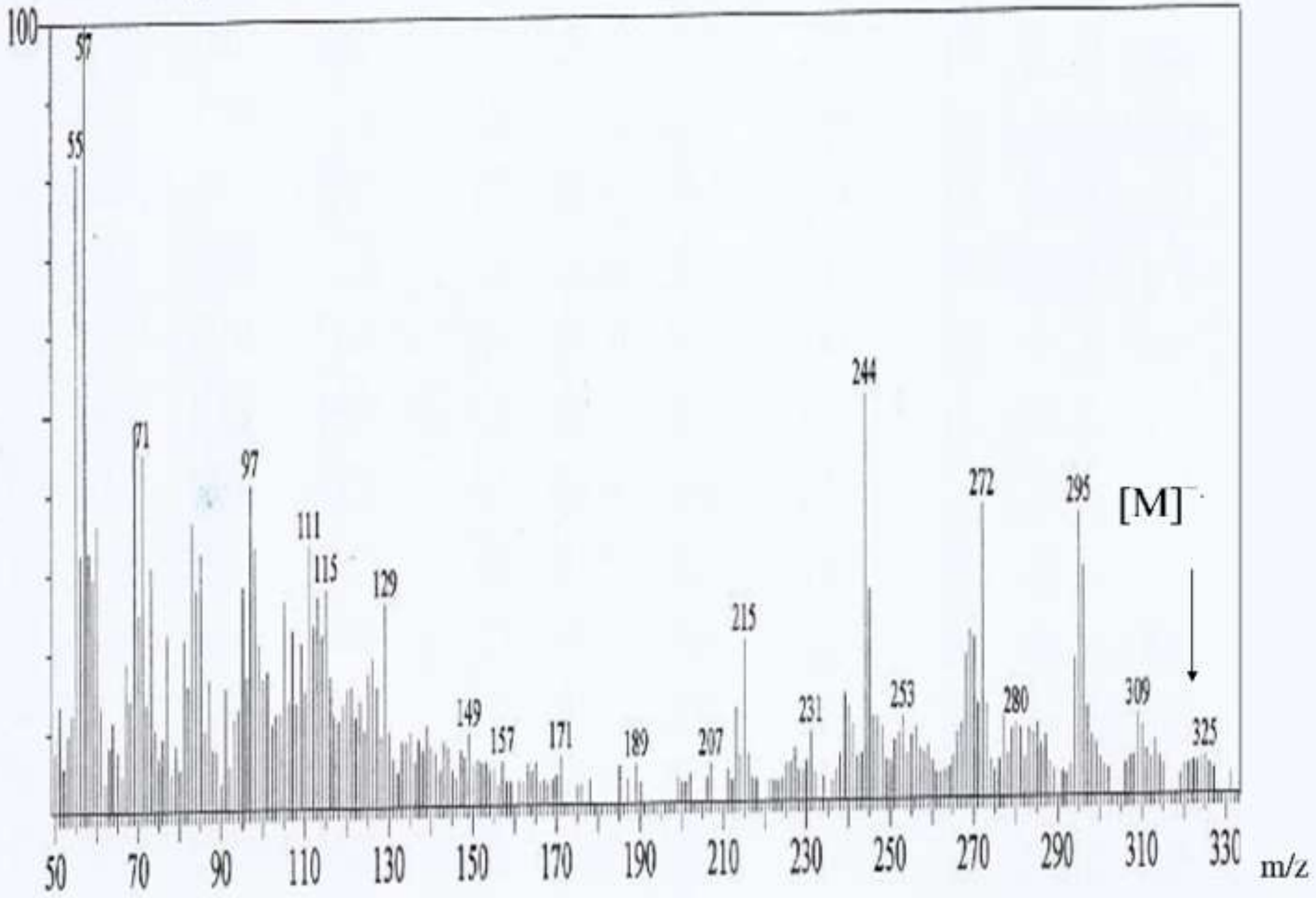

Information Classification: General

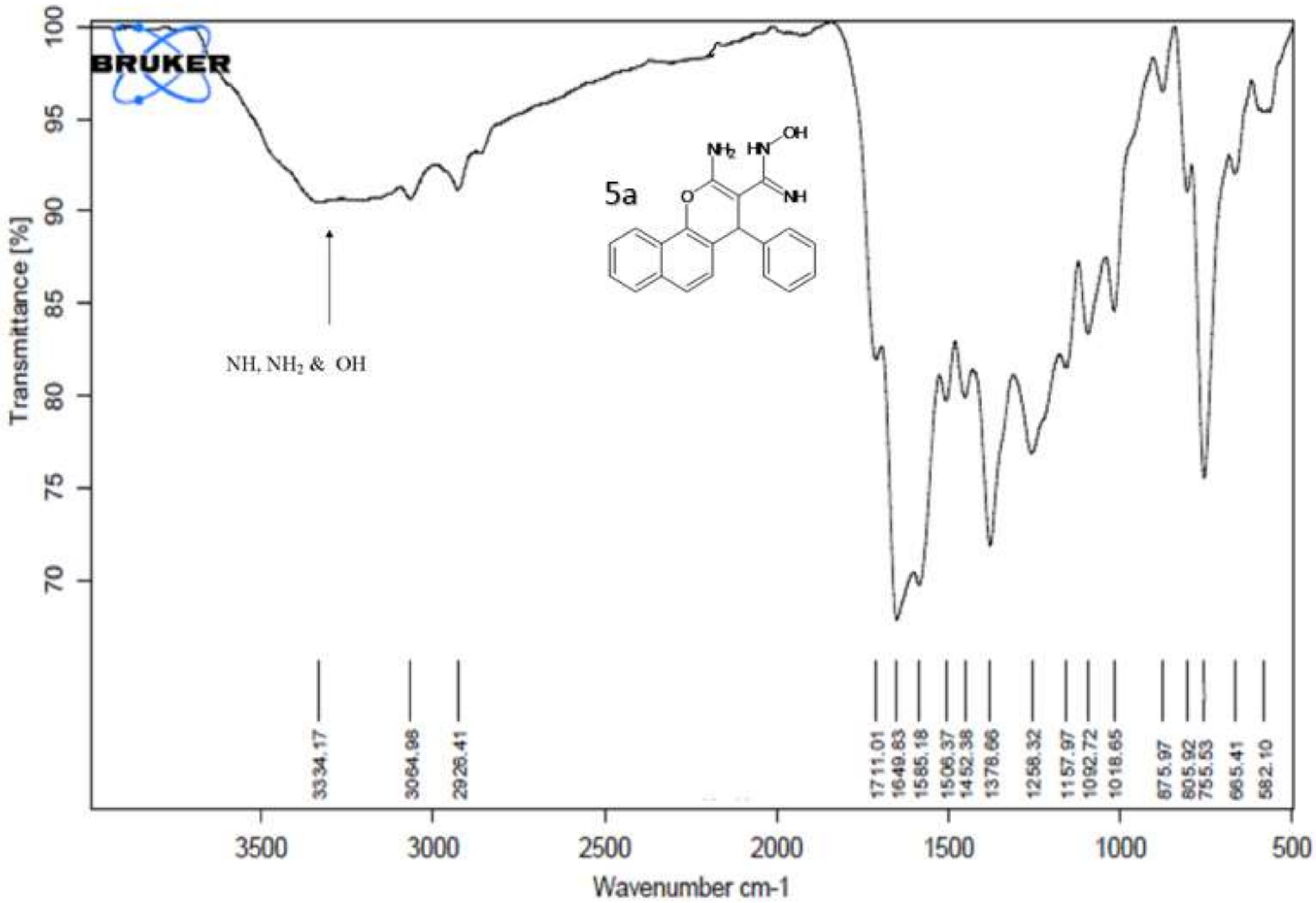

BRUKER
Transmittance [%]
100
95
90
85
80
75
70
5a
NH₂
HN
OH
NH
O
NH, NH₂ & OH
3334.17
3064.98
2926.41
1711.01
1649.63
1585.18
1506.37
1452.38
1378.66
1258.32
1157.97
1092.72
1018.65
875.97
805.92
755.53
665.41
582.10
3500
3000
2500
2000
1500
1000
500
Wavenumber cm-1

5a

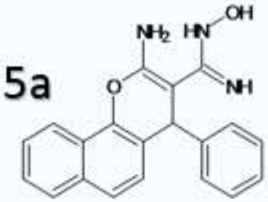

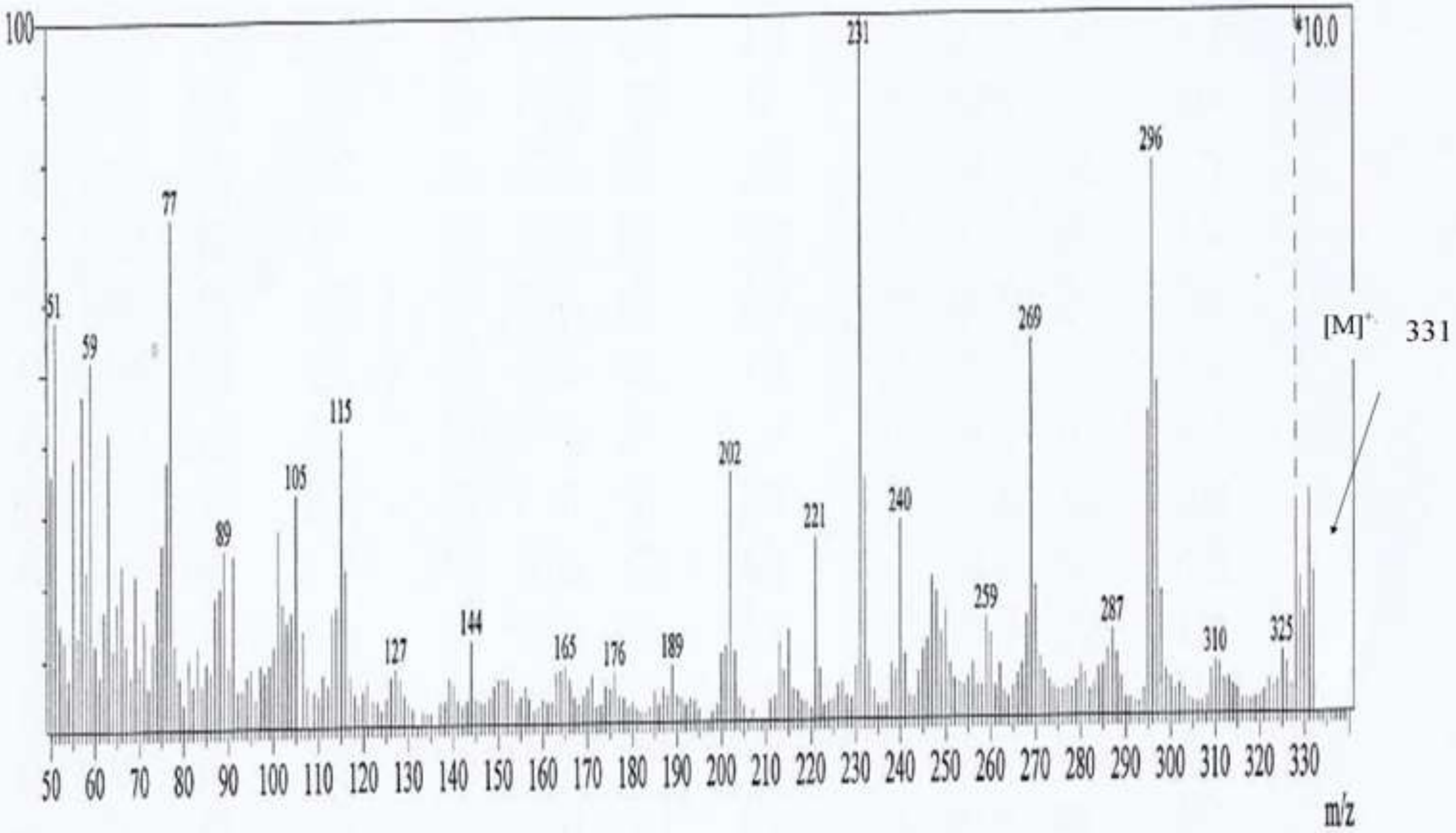

Information Classification: General

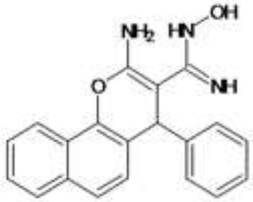

NH2
HN
OH
NH
O

5a Proton

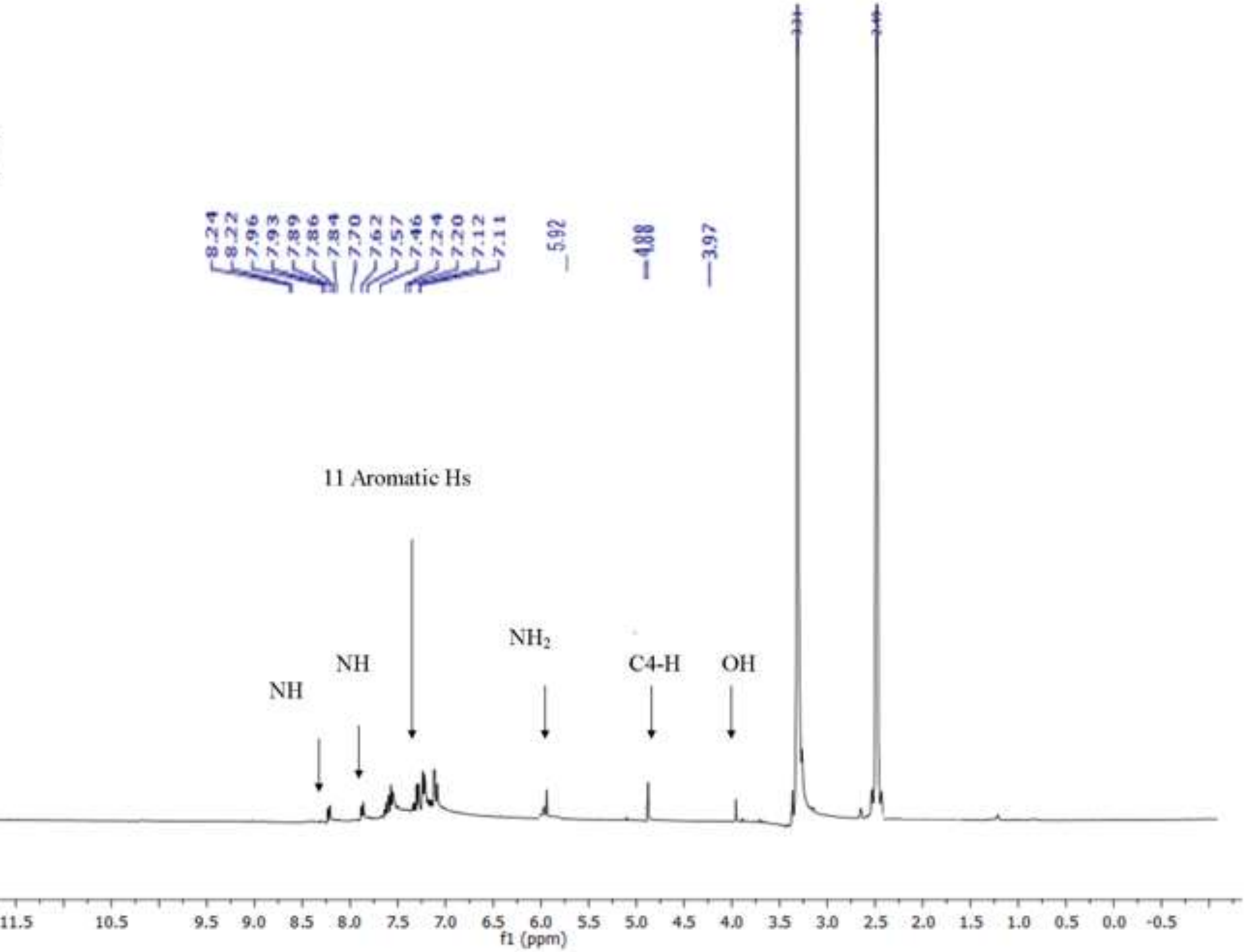

8.24
8.22
7.96
7.93
7.89
7.86
7.84
7.70
7.62
7.57
7.46
7.24
7.20
7.12
7.11
5.92
4.88
3.97
11 Aromatic Hs
NH
NH
NH2
C4-H
OH
12.5 11.5 10.5 9.5 9.0 8.5 8.0 7.5 7.0 6.5 6.0 5.5 5.0 4.5 4.0 3.5 3.0 2.5 2.0 1.5 1.0 0.5 0.0 -0.5
f1 (ppm)

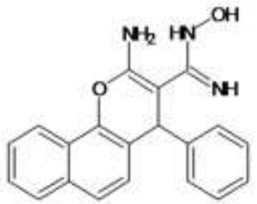

5a

Proton+ D_2O

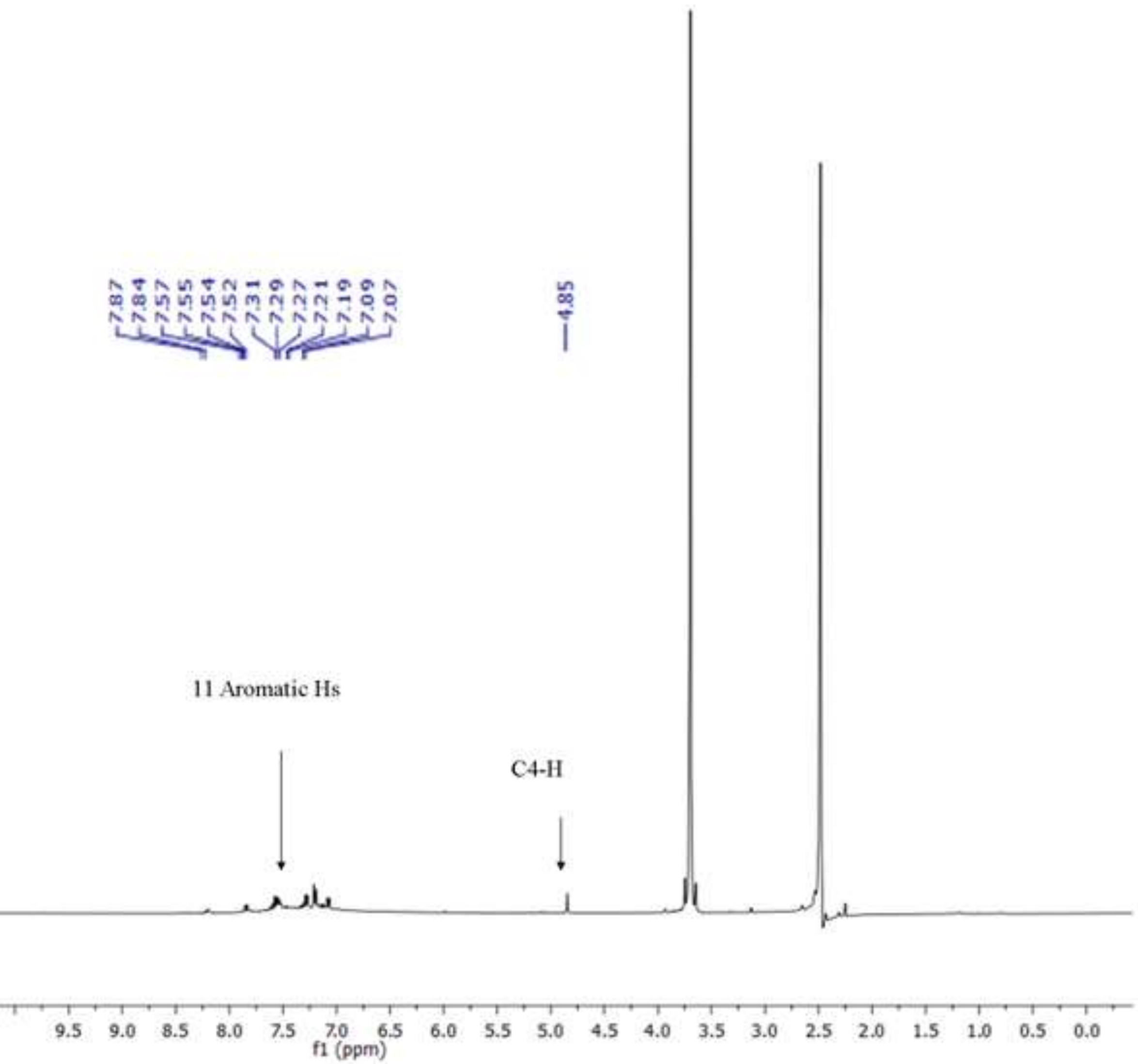

Information Classification: General

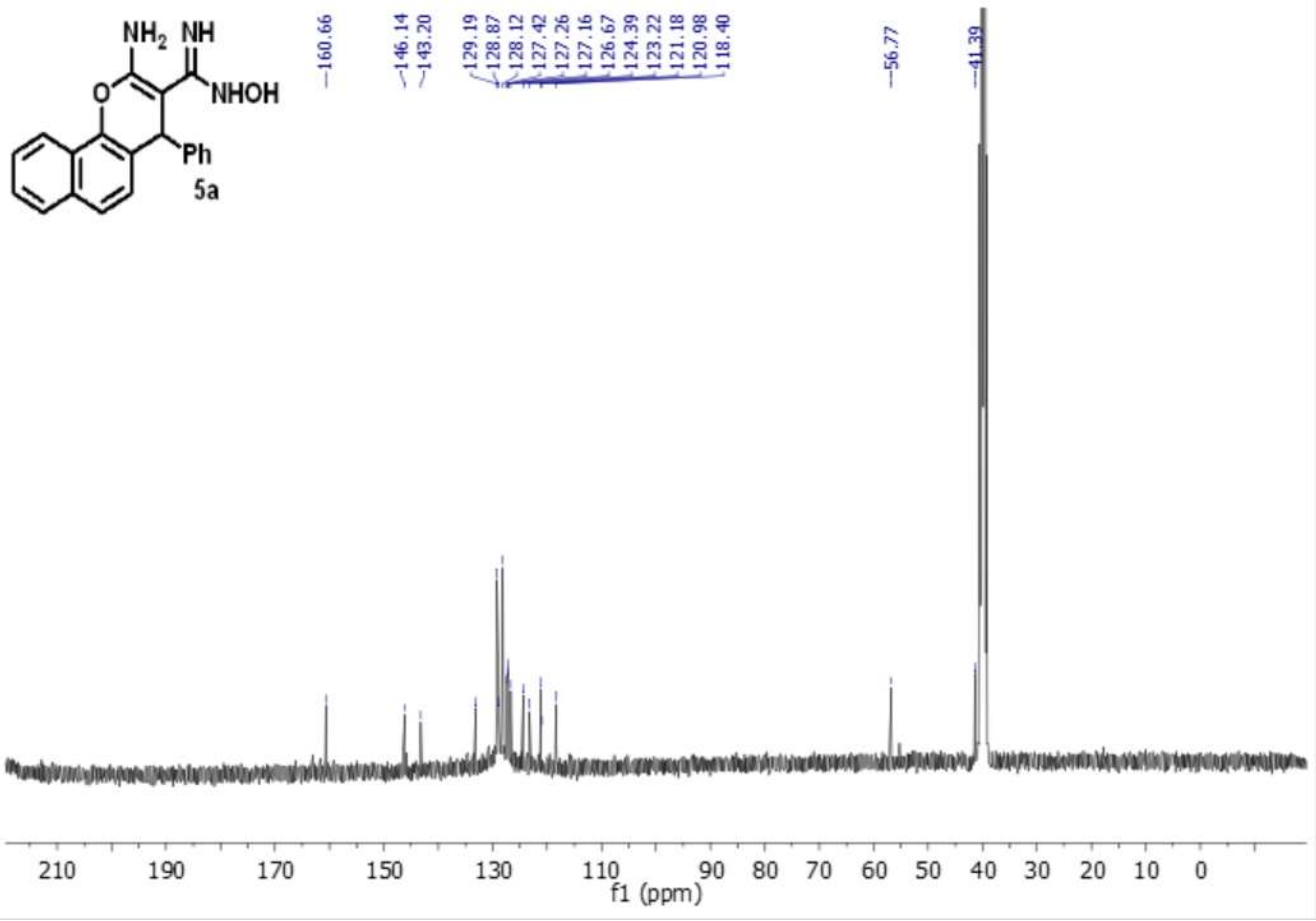
NH2
NH
O
NHOH
Ph
5a
160.66
146.14
143.20
129.19
128.87
128.12
127.42
127.26
127.16
126.67
124.39
123.22
121.18
120.98
118.40
56.77
41.39
210
190
170
150
130
110
90
80
70
60
50
40
30
20
10
0
f1 (ppm)

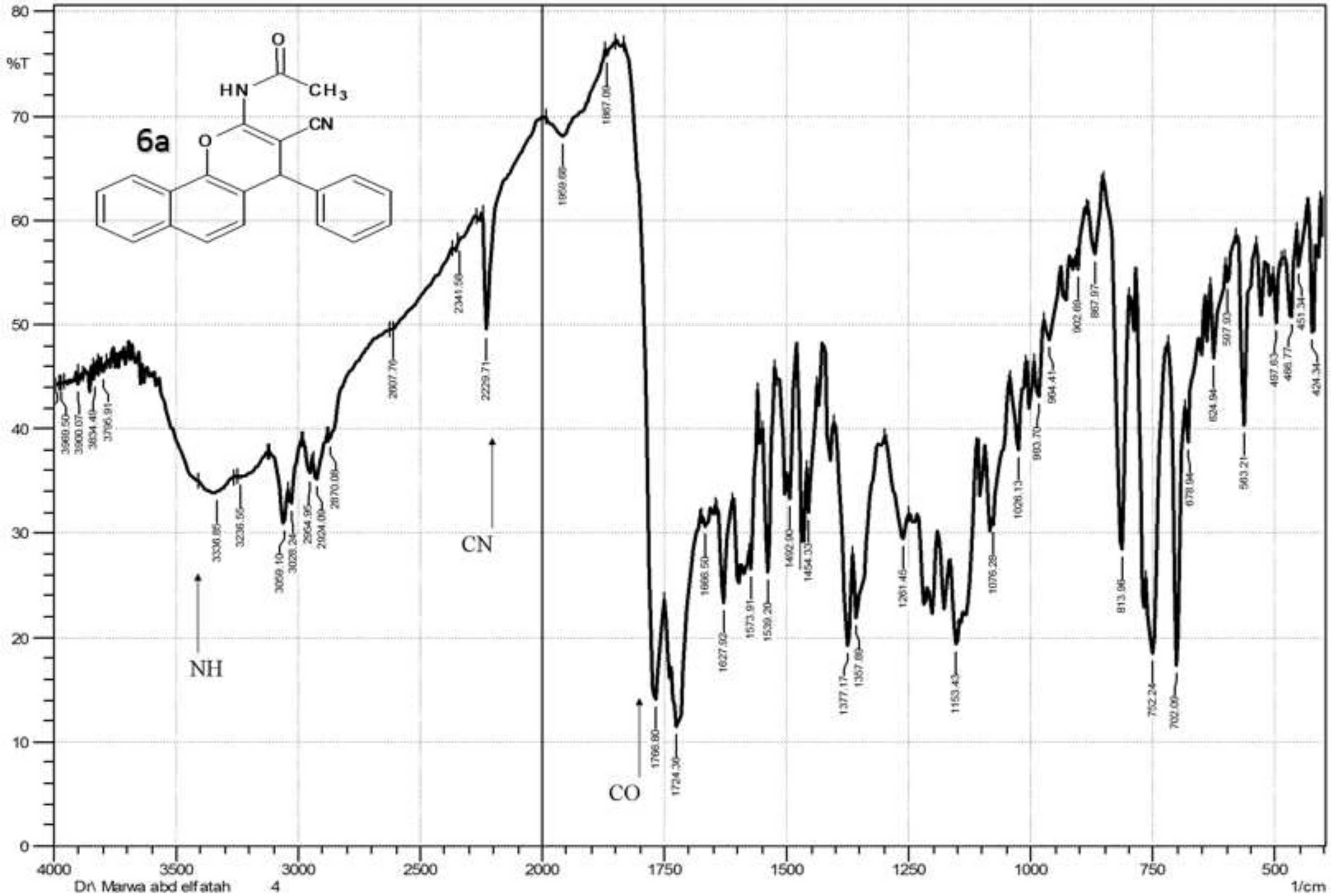

Information Classification: General

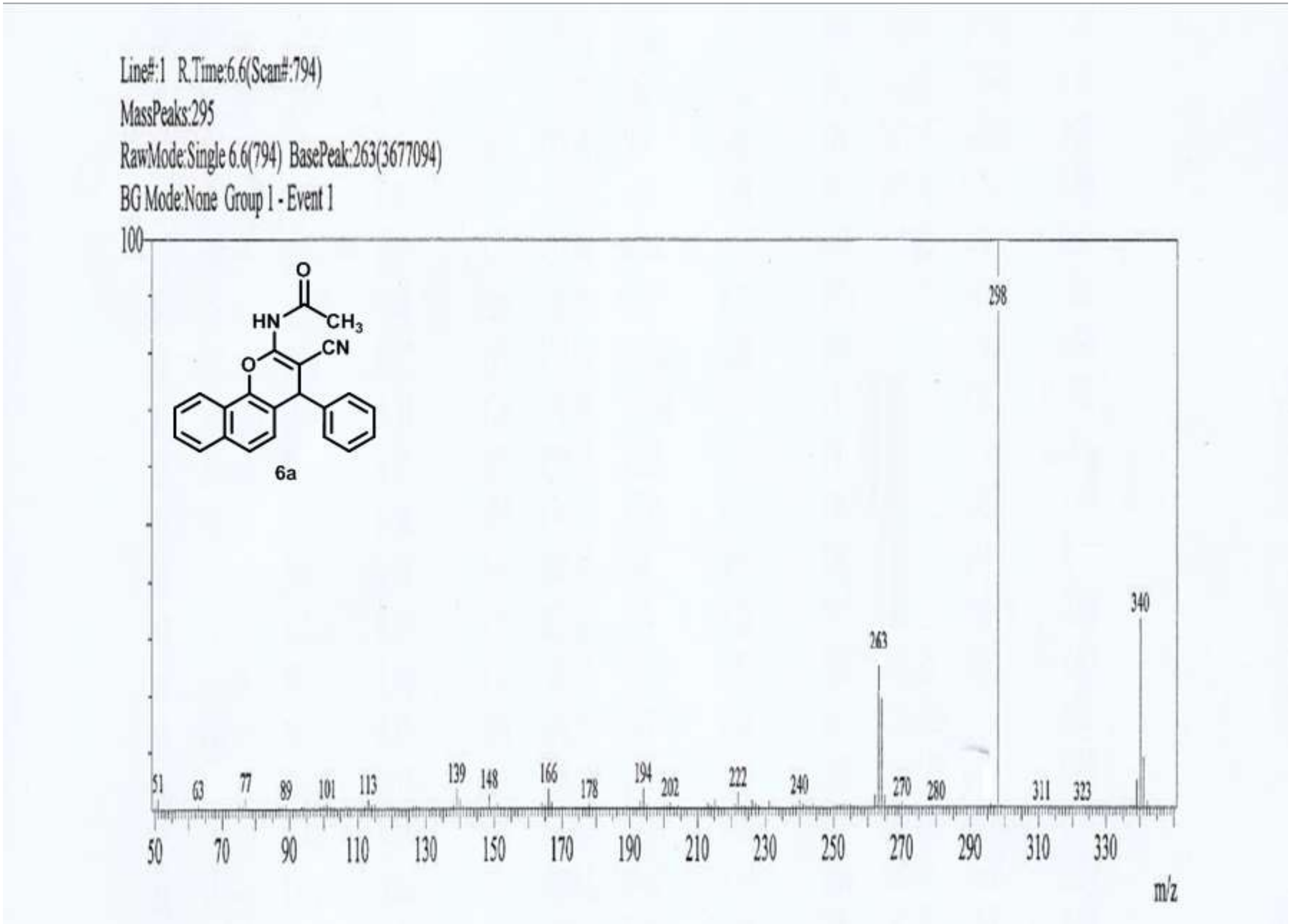
Line#:1 R.Time:6.6(Scan#:794)
MassPeaks:295
RawMode:Single 6.6(794) BasePeak:263(3677094)
BG Mode:None Group 1 - Event 1
100
HN
O
CH3
CN
O
6a
298
340
263
51
63
77
89
101
113
139
148
166
178
194
202
222
240
270
280
311
323
50
70
90
110
130
150
170
190
210
230
250
270
290
310
330
m/z

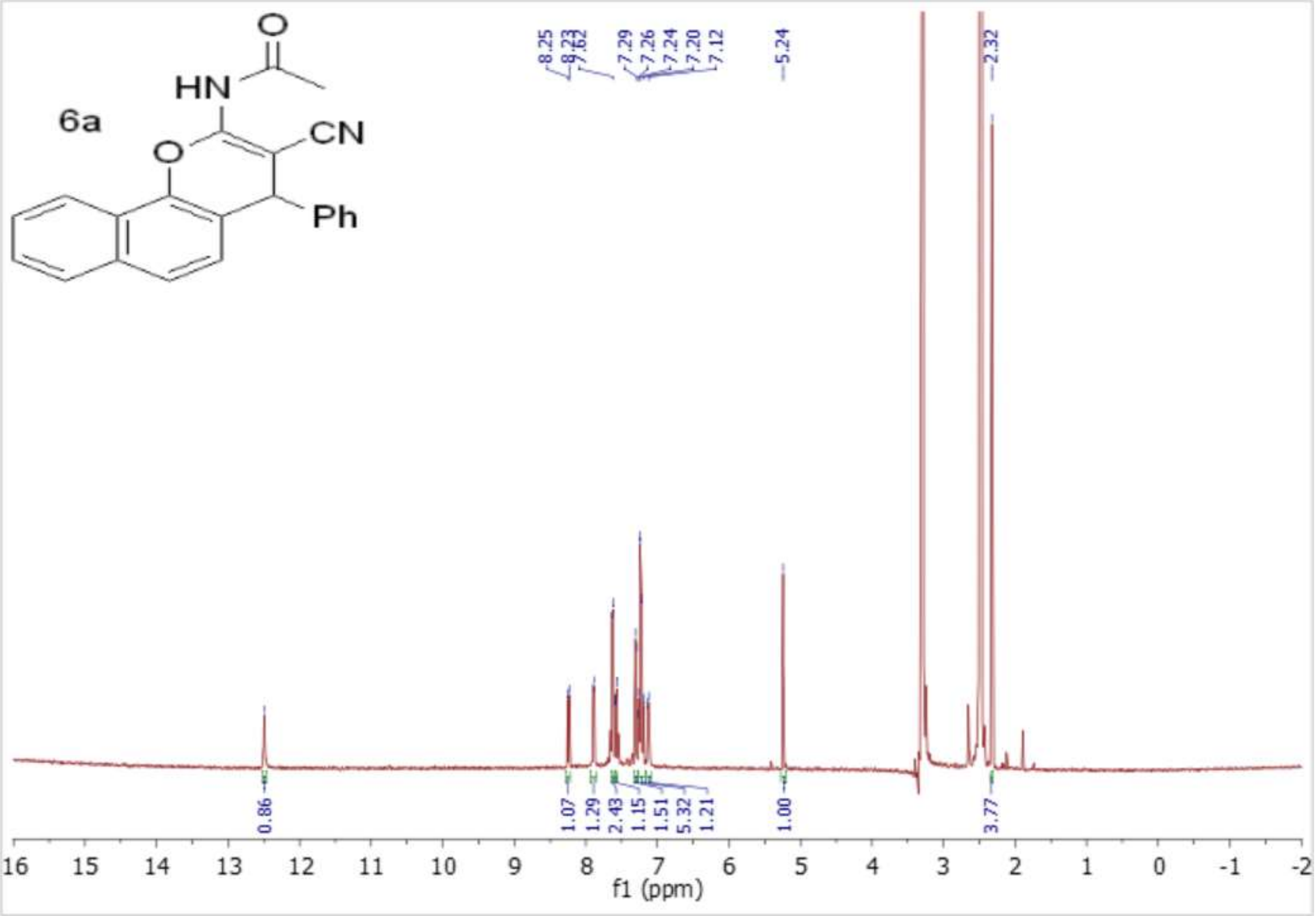
6a
HN
O
CN
Ph
8.25
8.23
7.62
7.29
7.26
7.24
7.20
7.12
5.24
2.32
0.86
1.07
1.29
2.43
1.15
1.51
5.32
1.21
1.00
3.77
f1 (ppm)

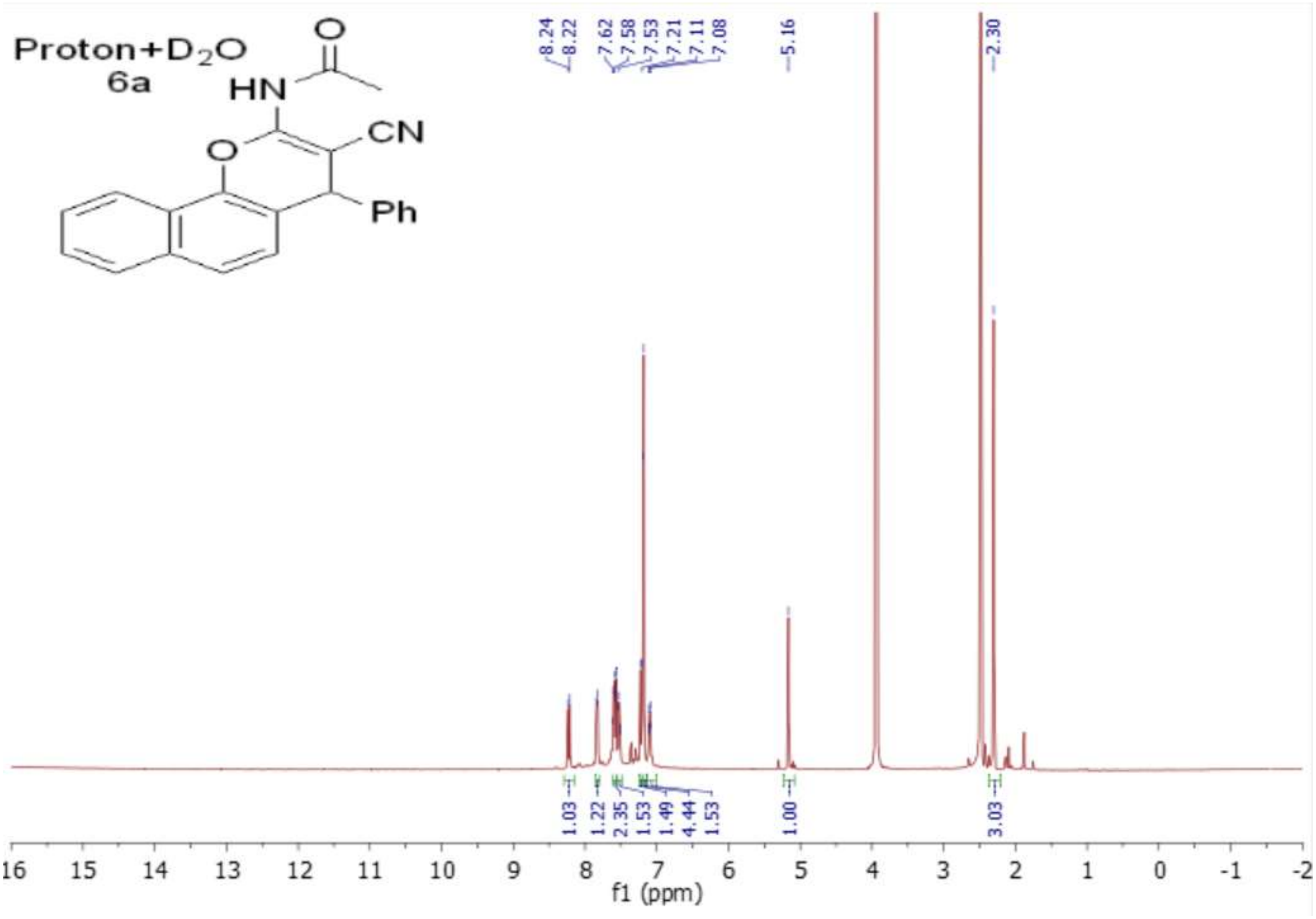

Information Classification: General

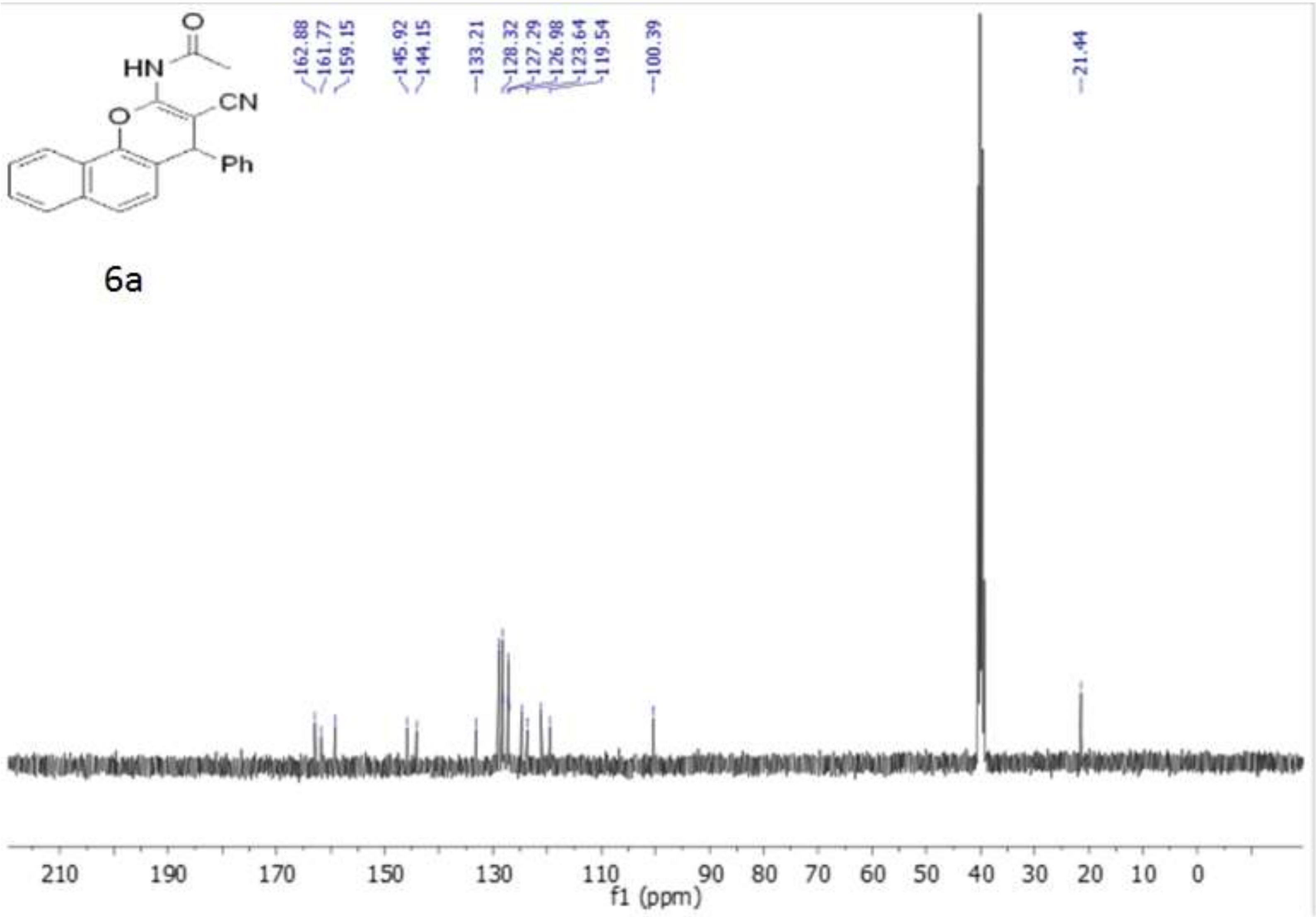

Information Classification: General

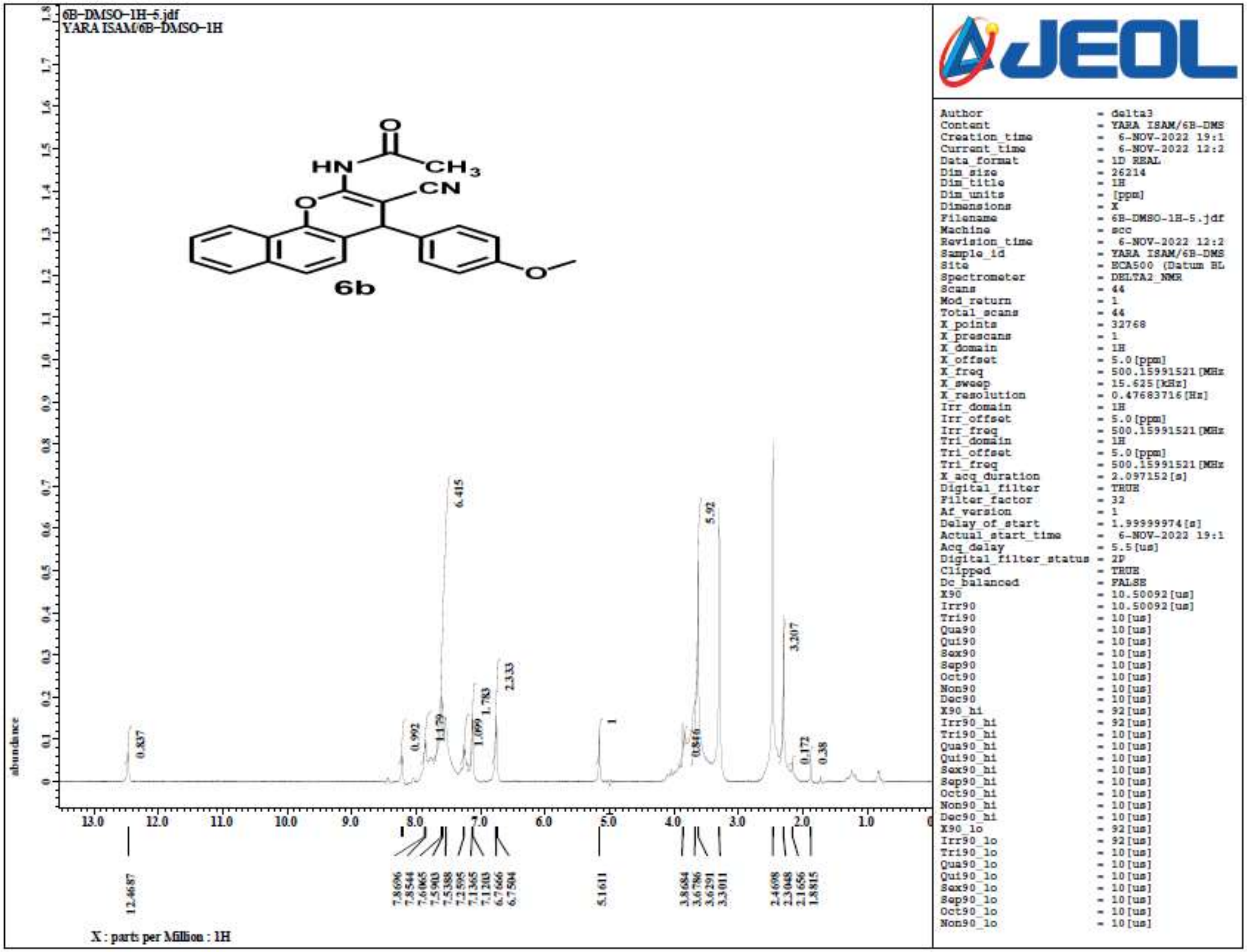

6B-DMSO-1H-5.jdf
YARA ISAM/6B-DMSO-1H
HN
O
CH3
CN
O
6b
JEOL
abundance
X : parts per Million : 1H
0.837
0.992
1.179
6.415
1.099
1.783
2.333
1
0.846
5.92
3.207
0.172
0.38
12.4687
7.8696
7.8544
7.6065
7.5903
7.5388
7.2595
7.1365
7.1203
6.7666
6.7504
5.1611
3.8684
3.6786
3.6291
3.3011
2.4698
2.3048
2.1656
1.8815
Author = delta3
Content = YARA ISAM/6B-DMS
Creation_time = 6-NOV-2022 19:1
Current_time = 6-NOV-2022 12:2
Data_format = 1D REAL
Dim_size = 26214
Dim_title = 1H
Dim_units = [ppm]
Dimensions = X
Filename = 6B-DMSO-1H-5.jdf
Machine = scc
Revision_time = 6-NOV-2022 12:2
Sample_id = YARA ISAM/6B-DMS
Site = ECA500 (Datum BL
Spectrometer = DELTA2_NMR
Scans = 44
Mod_return = 1
Total_scans = 44
X_points = 32768
X_prescans = 1
X_domain = 1H
X_offset = 5.0[ppm]
X_freq = 500.15991521[MHz
X_sweep = 15.625[kHz]
X_resolution = 0.47683716[Hz]
Irr_domain = 1H
Irr_offset = 5.0[ppm]
Irr_freq = 500.15991521[MHz
Tri_domain = 1H
Tri_offset = 5.0[ppm]
Tri_freq = 500.15991521[MHz
X_acq_duration = 2.097152[s]
Digital_filter = TRUE
Filter_factor = 32
Af_version = 1
Delay_of_start = 1.99999974[s]
Actual_start_time = 6-NOV-2022 19:1
Acq_delay = 5.5[us]
Digital_filter_status = 2P
Clipped = TRUE
Dc_balanced = FALSE
X90 = 10.50092[us]
Irr90 = 10.50092[us]
Tri90 = 10[us]
Qua90 = 10[us]
Qui90 = 10[us]
Sex90 = 10[us]
Sep90 = 10[us]
Oct90 = 10[us]
Non90 = 10[us]
Dec90 = 10[us]
X90_hi = 92[us]
Irr90_hi = 92[us]
Tri90_hi = 10[us]
Qua90_hi = 10[us]
Qui90_hi = 10[us]
Sex90_hi = 10[us]
Sep90_hi = 10[us]
Oct90_hi = 10[us]
Non90_hi = 10[us]
Dec90_hi = 10[us]
X90_lo = 92[us]
Irr90_lo = 92[us]
Tri90_lo = 10[us]
Qua90_lo = 10[us]
Qui90_lo = 10[us]
Sex90_lo = 10[us]
Sep90_lo = 10[us]
Oct90_lo = 10[us]
Non90_lo = 10[us]

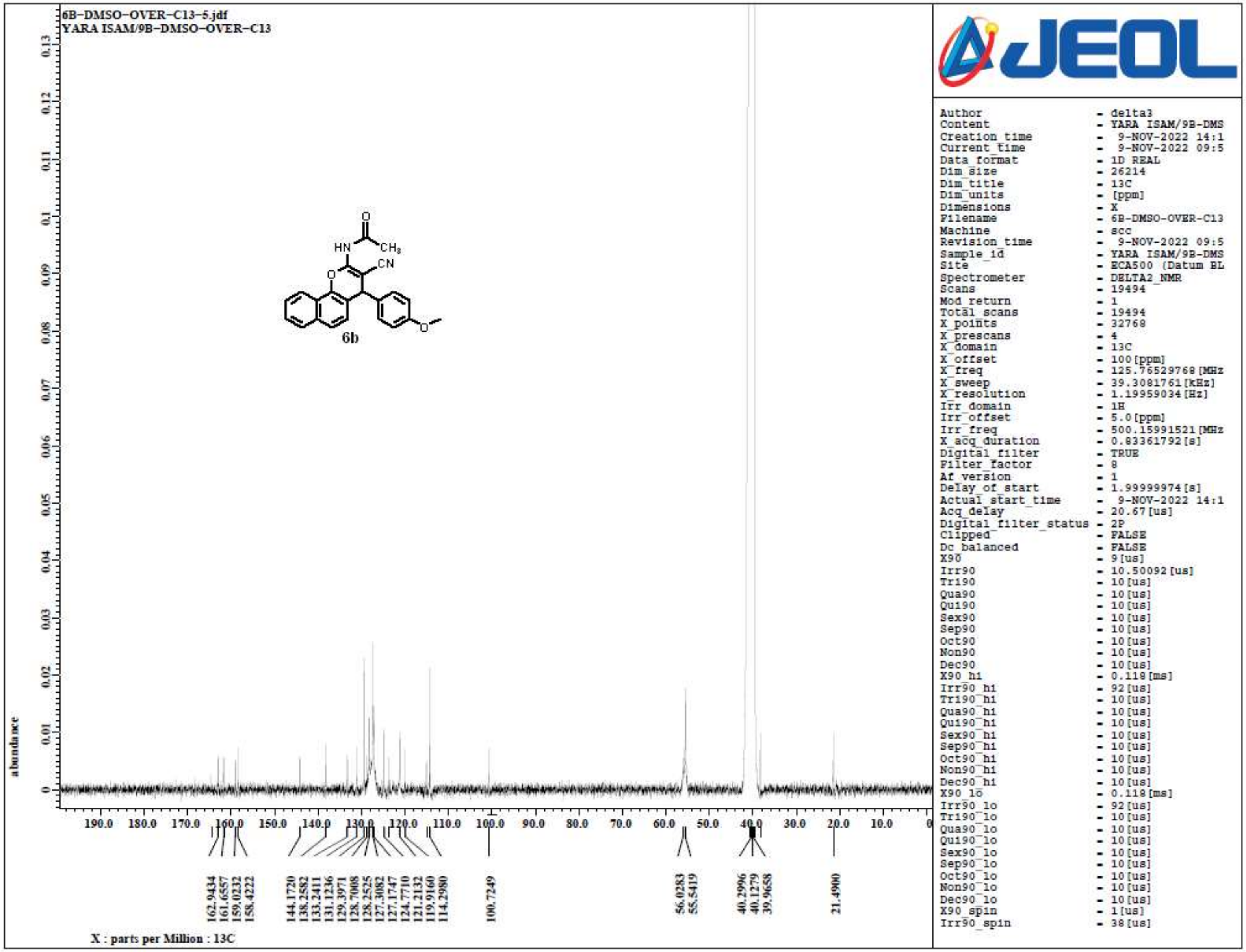
6B-DMSO-OVER-C13-5.jdf
YARA ISAM/9B-DMSO-OVER-C13
HN
O
CH3
CN
O
O
6b
abundance
X : parts per Million : 13C
162.9434
161.6557
159.0232
158.4222
144.1720
138.2582
133.2411
131.1236
129.3971
128.7008
128.2525
127.3082
127.1747
124.7710
121.2132
119.9160
114.2980
100.7249
56.0283
55.5419
40.2996
40.1279
39.9658
21.4900
JEOL
Author - delta3
Content - YARA ISAM/9B-DMS
Creation_time - 9-NOV-2022 14:1
Current_time - 9-NOV-2022 09:5
Data_format - 1D REAL
Dim_size - 26214
Dim_title - 13C
Dim_units - [ppm]
Dimensions - X
Filename - 6B-DMSO-OVER-C13
Machine - scc
Revision_time - 9-NOV-2022 09:5
Sample_id - YARA ISAM/9B-DMS
Site - ECA500 (Datum BL
Spectrometer - DELTA2_NMR
Scans - 19494
Mod_return - 1
Total_scans - 19494
X_points - 32768
X_prescans - 4
X_domain - 13C
X_offset - 100[ppm]
X_freq - 125.76529768[MHz
X_sweep - 39.3081761[kHz]
X_resolution - 1.19959034[Hz]
Irr_domain - 1H
Irr_offset - 5.0[ppm]
Irr_freq - 500.15991521[MHz
X_acq_duration - 0.83361792[s]
Digital_filter - TRUE
Filter_factor - 8
Af_version - 1
Delay_of_start - 1.99999974[s]
Actual_start_time - 9-NOV-2022 14:1
Acq_delay - 20.67[us]
Digital_filter_status - 2P
Clipped - FALSE
Dc_balanced - FALSE
X90 - 9[us]
Irr90 - 10.50092[us]
Tri90 - 10[us]
Qua90 - 10[us]
Qui90 - 10[us]
Sex90 - 10[us]
Sep90 - 10[us]
Oct90 - 10[us]
Non90 - 10[us]
Dec90 - 10[us]
X90_hi - 0.118[ms]
Irr90_hi - 92[us]
Tri90_hi - 10[us]
Qua90_hi - 10[us]
Qui90_hi - 10[us]
Sex90_hi - 10[us]
Sep90_hi - 10[us]
Oct90_hi - 10[us]
Non90_hi - 10[us]
Dec90_hi - 10[us]
X90_lo - 0.118[ms]
Irr90_lo - 92[us]
Tri90_lo - 10[us]
Qua90_lo - 10[us]
Qui90_lo - 10[us]
Sex90_lo - 10[us]
Sep90_lo - 10[us]
Oct90_lo - 10[us]
Non90_lo - 10[us]
Dec90_lo - 10[us]
X90_spin - 1[us]
Irr90_spin - 38[us]

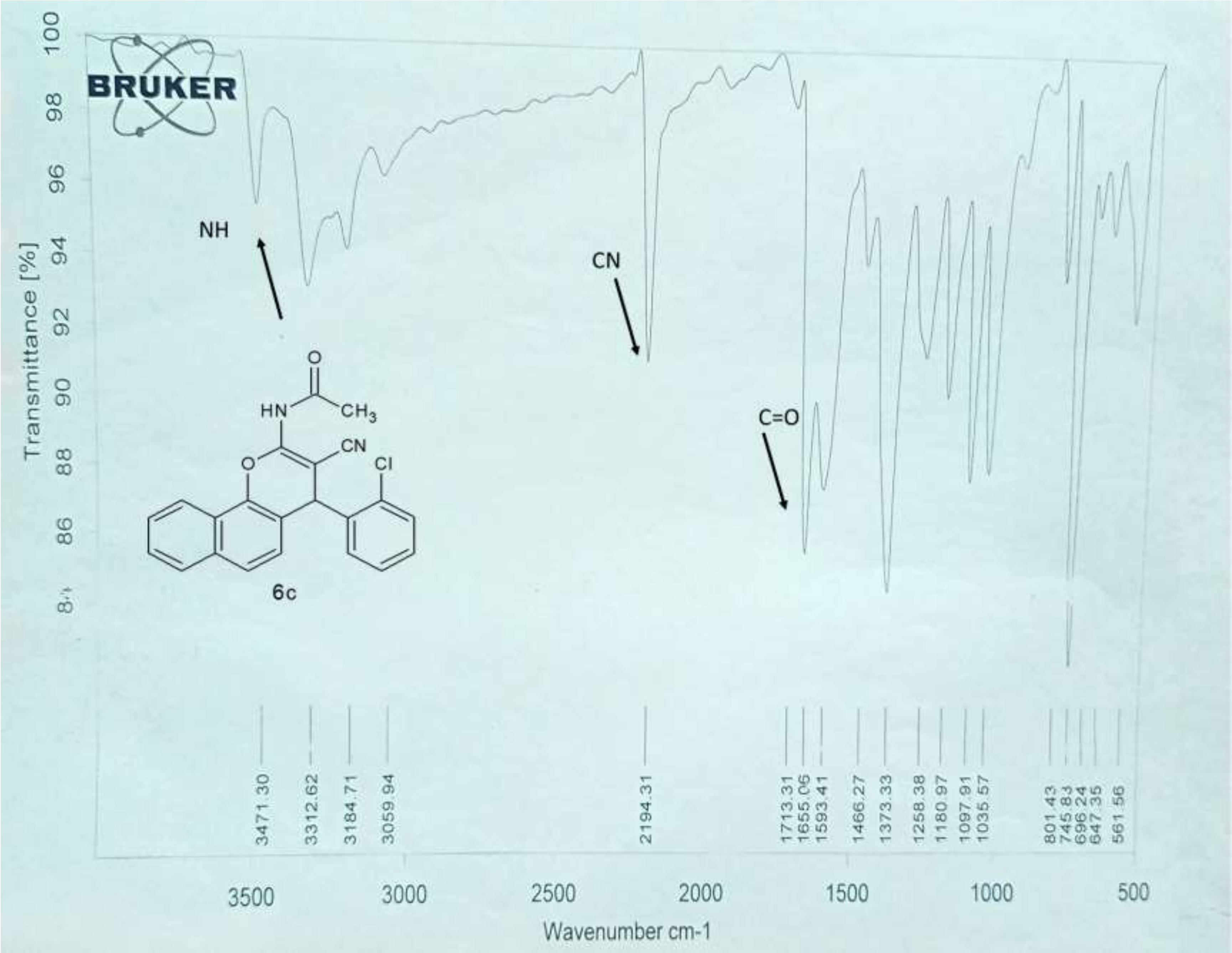
BRUKER
Transmittance [%]
100
98
96
94
92
90
88
86
84
NH
CN
C=O
HN
O
CH3
CN
Cl
O
6c
3471.30
3312.62
3184.71
3059.94
2194.31
1713.31
1655.06
1593.41
1466.27
1373.33
1258.38
1180.97
1097.91
1035.57
801.43
745.83
696.24
647.35
561.56
3500
3000
2500
2000
1500
1000
500
Wavenumber cm-1

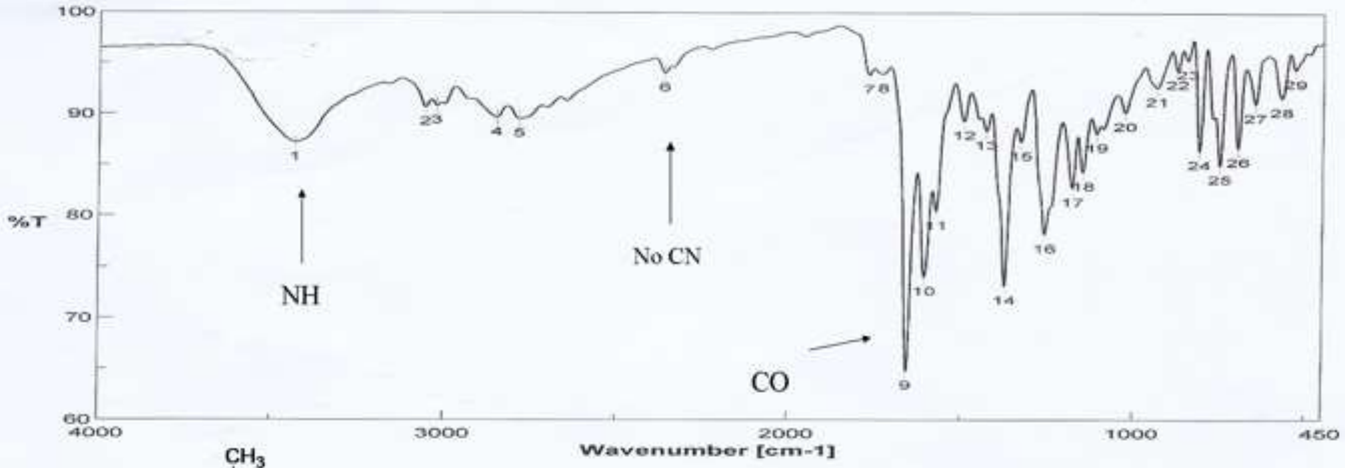

7a

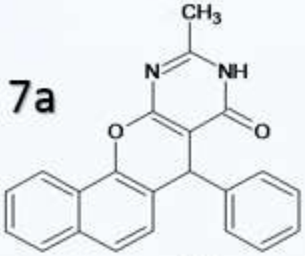

No.	cm-1	%T	No.	cm-1	%T	No.	cm-1	%T
1	3432.67	87.2096	2	3057.58	90.7144	3	3024.8	90.8619
4	2850.27	89.7	5	2784.71	89.5753	6	2362.37	94.0795
7	1769.37	93.9275	8	1727.91	94.0204	9	1656.55	64.8303
10	1605.45	74.1241	11	1572.66	80.5227	12	1494.56	89.4049
13	1429.96	88.4472	14	1374.03	73.2284	15	1329.68	87.3598
16	1258.32	78.2788	17	1180.22	82.8093	18	1149.37	84.3935
19	1110.8	88.1912	20	1027.87	90.261	21	935.306	92.6944
22	875.524	94.369	23	845.633	95.3729	24	809.956	86.5002
25	750.174	85.0681	26	697.141	86.7868	27	647.965	91.1278
28	570.826	91.6866	29	532.257	94.4609			

7a proton

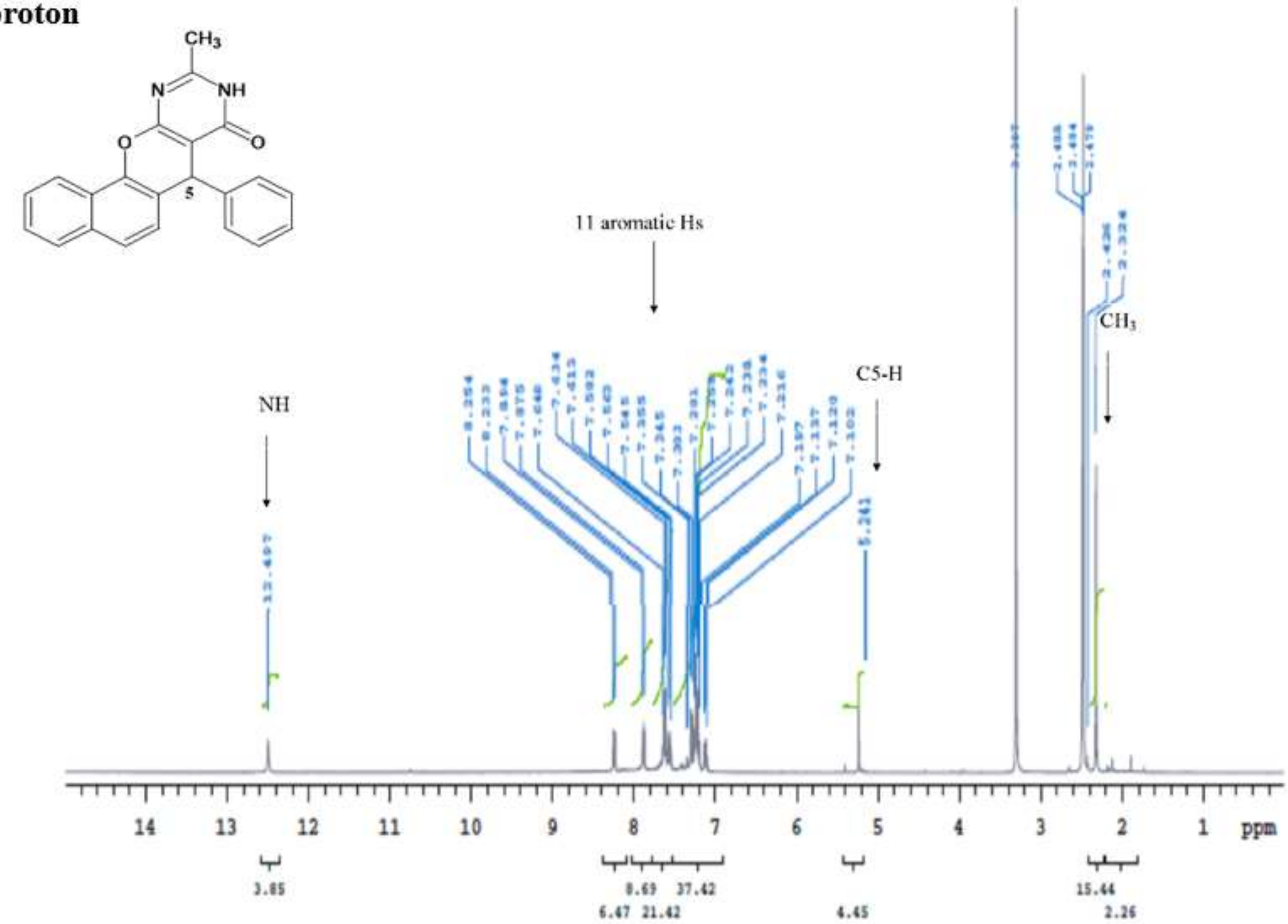

Information Classification: General

7a proton +D_2O

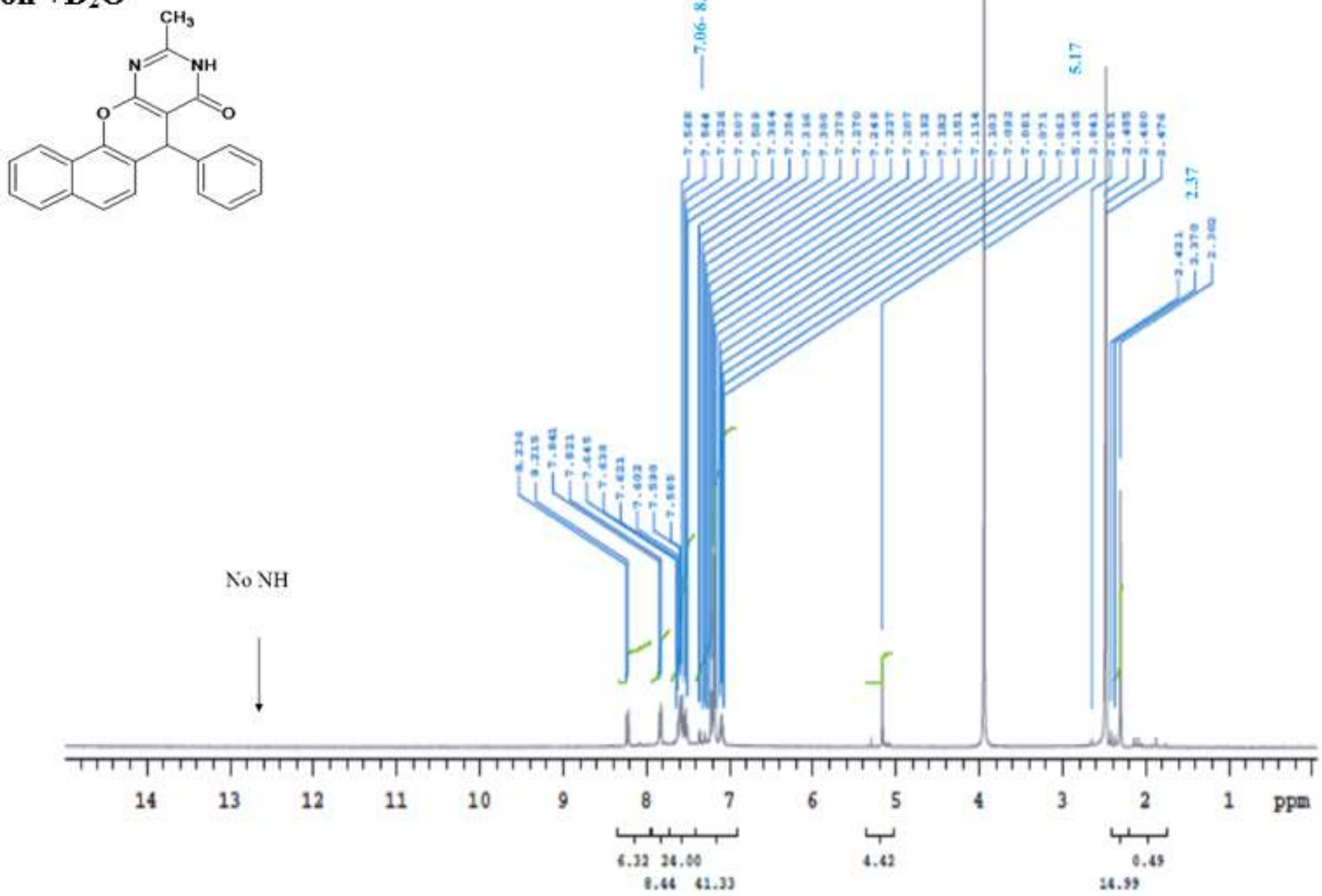

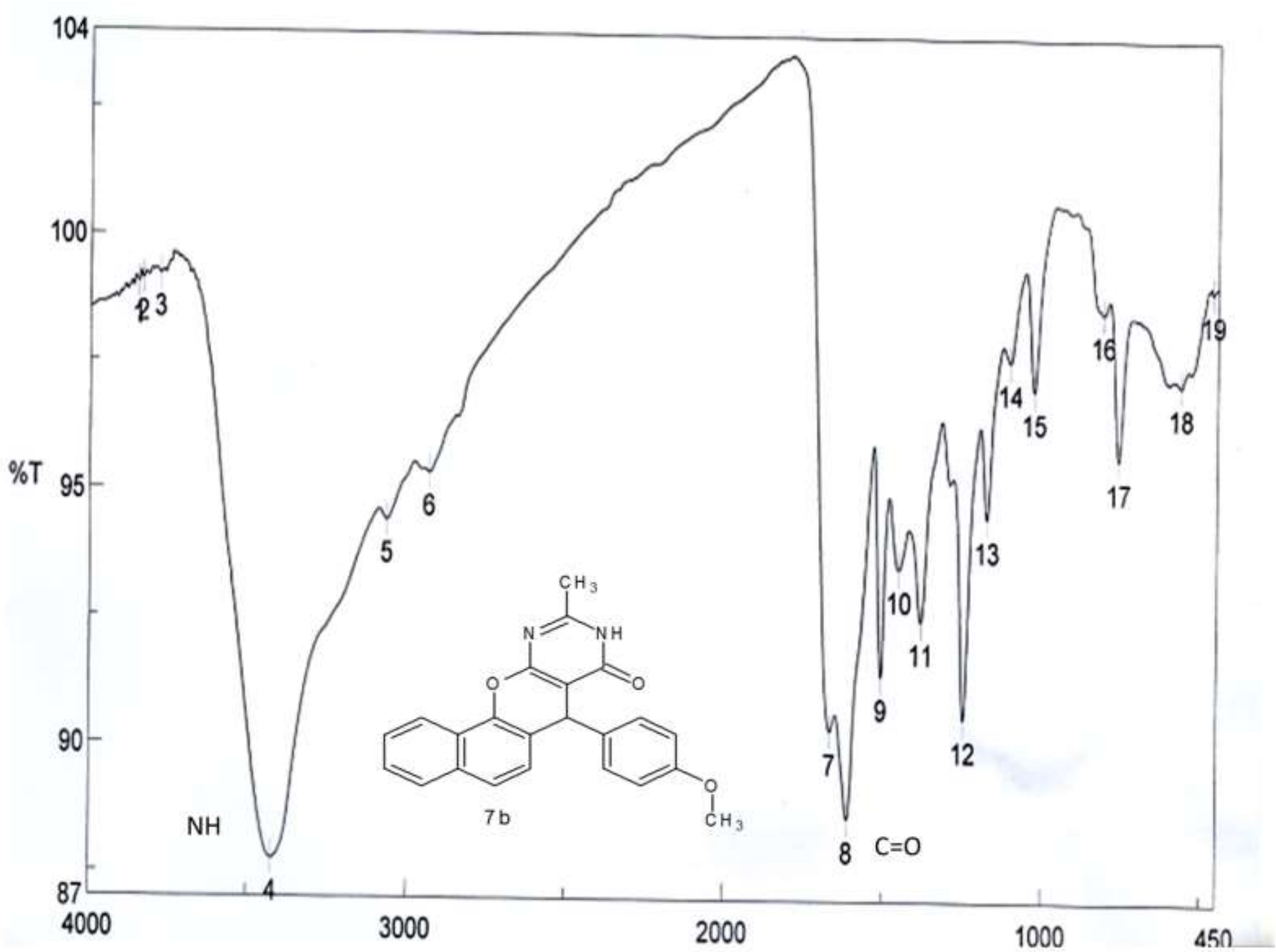
104
100
95
90
87
%T
4000
3000
2000
1000
450
NH
C=O
7b
CH3
N
NH
O
O
O
CH3
1 2 3 4 5 6 7 8 9 10 11 12 13 14 15 16 17 18 19

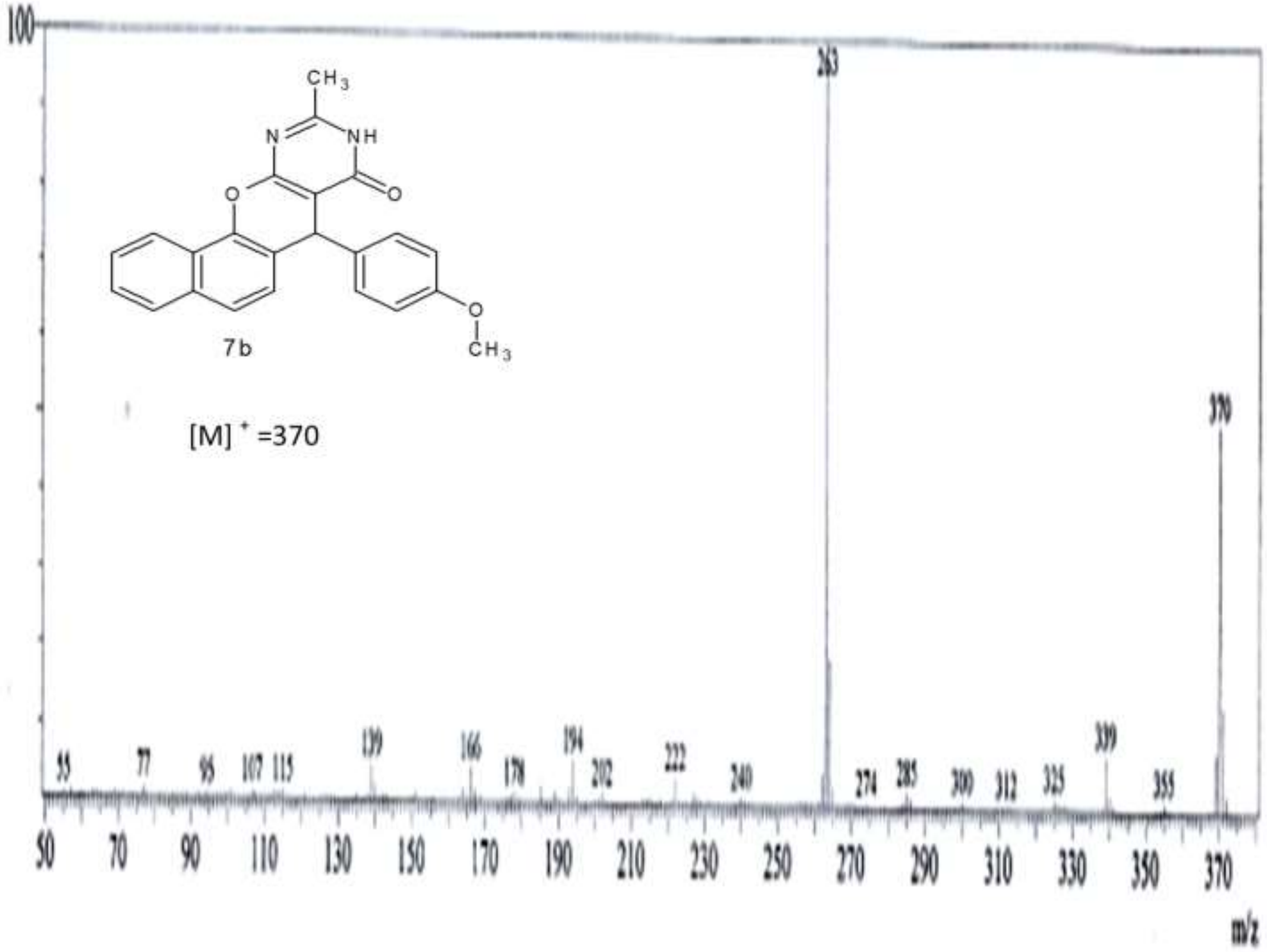

Information Classification: General

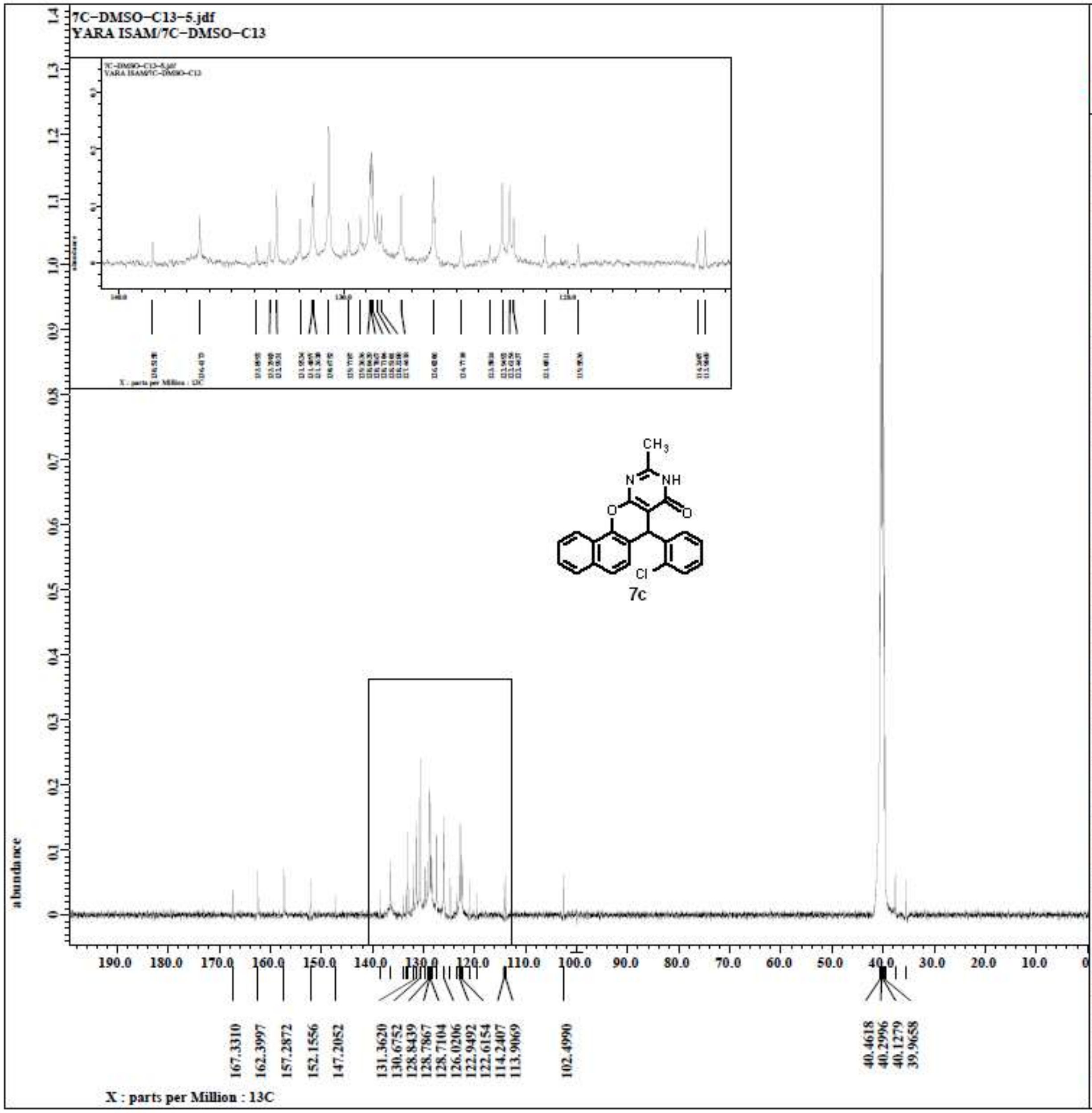

JEOL

Author	= delta3
Content	= YARA ISAM/7C-DMS
Creation_time	= 8-NOV-2022 20:3
Current_time	= 9-NOV-2022 09:5
Data_format	= 1D REAL
Dim_size	= 26214
Dim_title	= 13C
Dim_units	= [ppm]
Dimensions	= X
Filename	= 7C-DMSO-C13-5.jd
Machine	= scc
Revision_time	= 9-NOV-2022 09:5
Sample_id	= YARA ISAM/7C-DMS
Site	= ECA500 (Datum BL
Spectrometer	= DELTA2_NMR
Scans	= 690
Mod_return	= 1
Total_scans	= 690
X_points	= 32768
X_prescans	= 4
X_domain	= 13C
X_offset	= 100[ppm]
X_freq	= 125.76529768[MHz
X_sweep	= 39.3081761[kHz]
X_resolution	= 1.19959034[Hz]
Irr_domain	= 1H
Irr_offset	= 5.0[ppm]
Irr_freq	= 500.15991521[MHz
X_acq_duration	= 0.83361792[s]
Digital_filter	= TRUE
Filter_factor	= 8
Af_version	= 1
Delay_of_start	= 1.99999974[s]
Actual_start_time	= 8-NOV-2022 20:3
Acq_delay	= 20.67[us]
Digital_filter_status	= 2P
Clipped	= FALSE
Dc_balanced	= FALSE
X90	= 9[us]
Irr90	= 10.50092[us]
Tri90	= 10[us]
Qua90	= 10[us]
Qui90	= 10[us]
Sex90	= 10[us]
Sep90	= 10[us]
Oct90	= 10[us]
Non90	= 10[us]
Dec90	= 10[us]
X90_hi	= 0.118[ms]
Irr90_hi	= 92[us]
Tri90_hi	= 10[us]
Qua90_hi	= 10[us]
Qui90_hi	= 10[us]
Sex90_hi	= 10[us]
Sep90_hi	= 10[us]
Oct90_hi	= 10[us]
Non90_hi	= 10[us]
Dec90_hi	= 10[us]
X90_lo	= 0.118[ms]
Irr90_lo	= 92[us]
Tri90_lo	= 10[us]
Qua90_lo	= 10[us]
Qui90_lo	= 10[us]
Sex90_lo	= 10[us]
Sep90_lo	= 10[us]
Oct90_lo	= 10[us]
Non90_lo	= 10[us]
Dec90_lo	= 10[us]
X90_spin	= 1[us]
Irr90_spin	= 38[us]

Information Classification: General

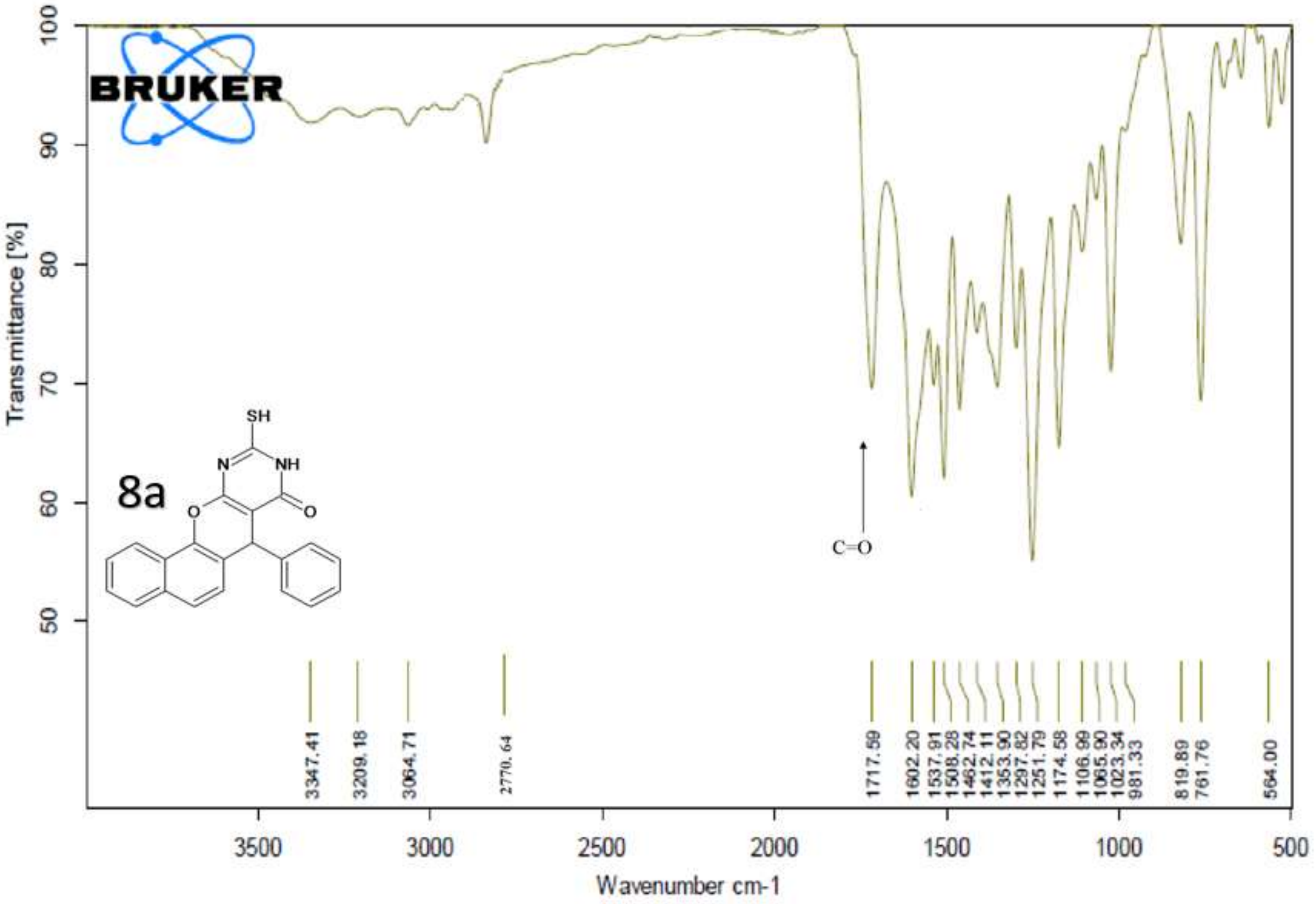

Information Classification: General

8a

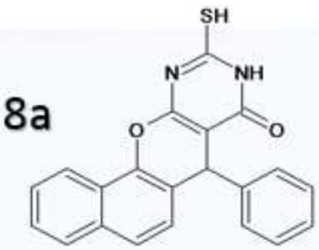

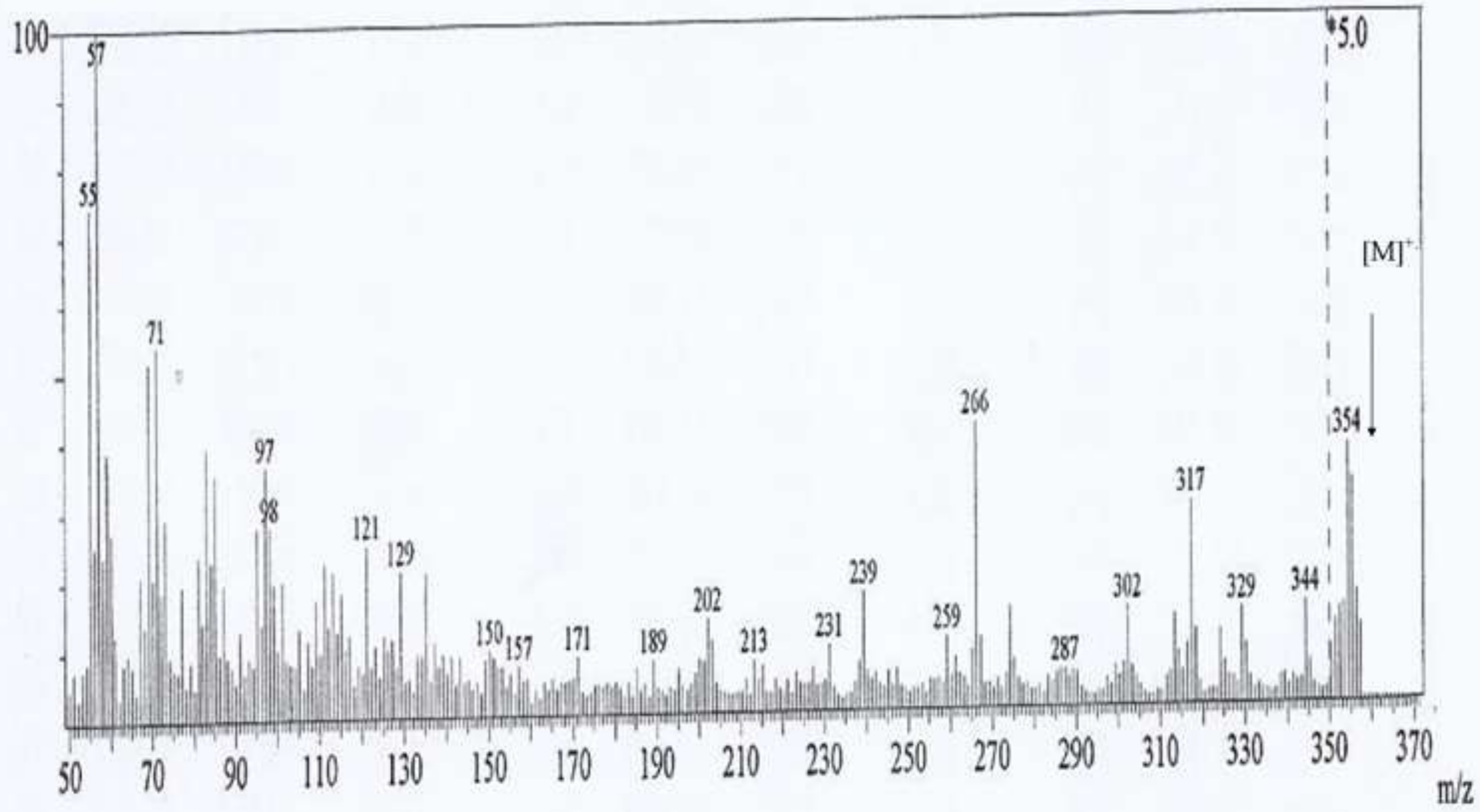

Information Classification: General

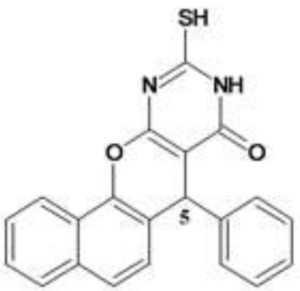

8a Proton

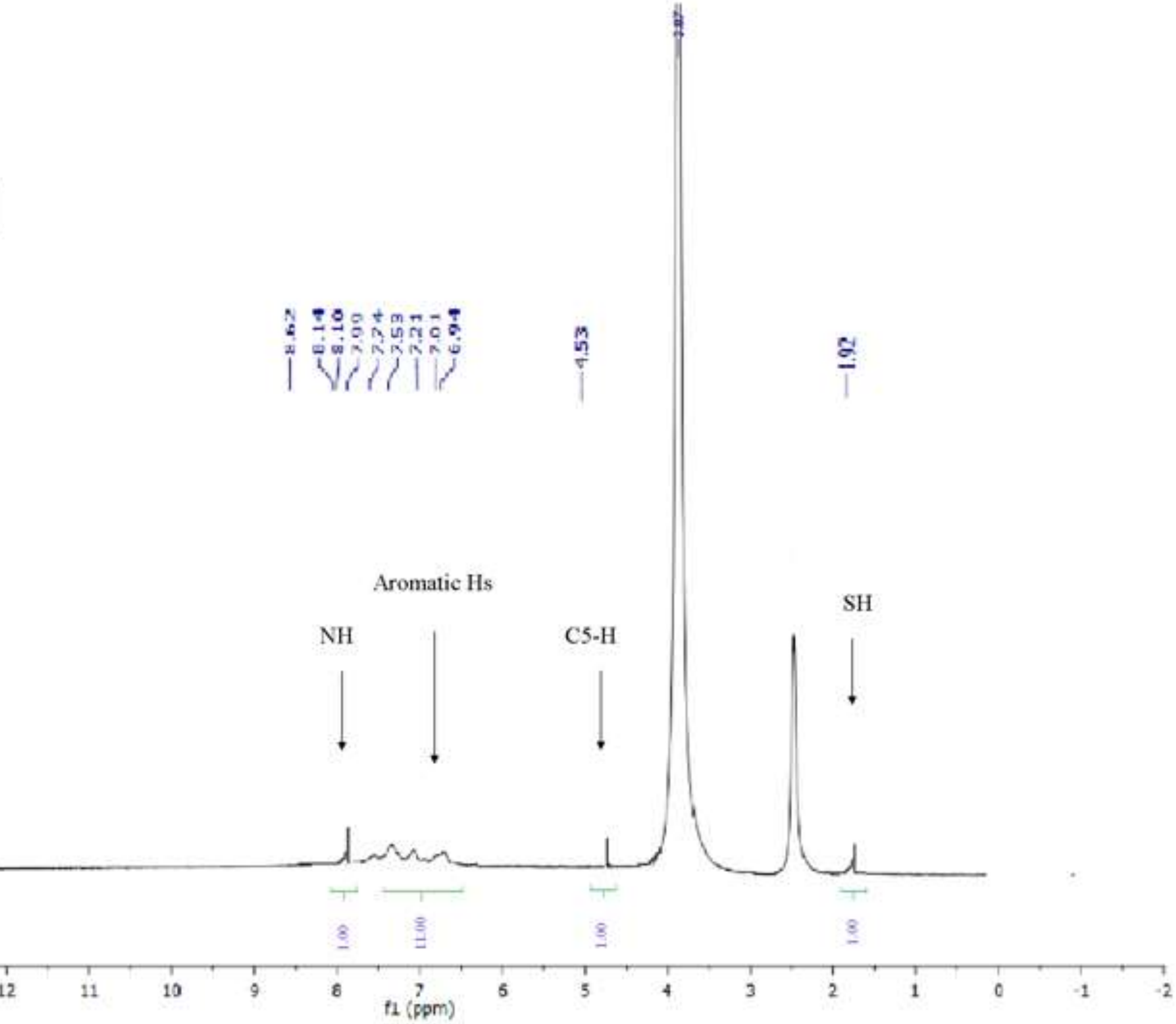

Information Classification: General

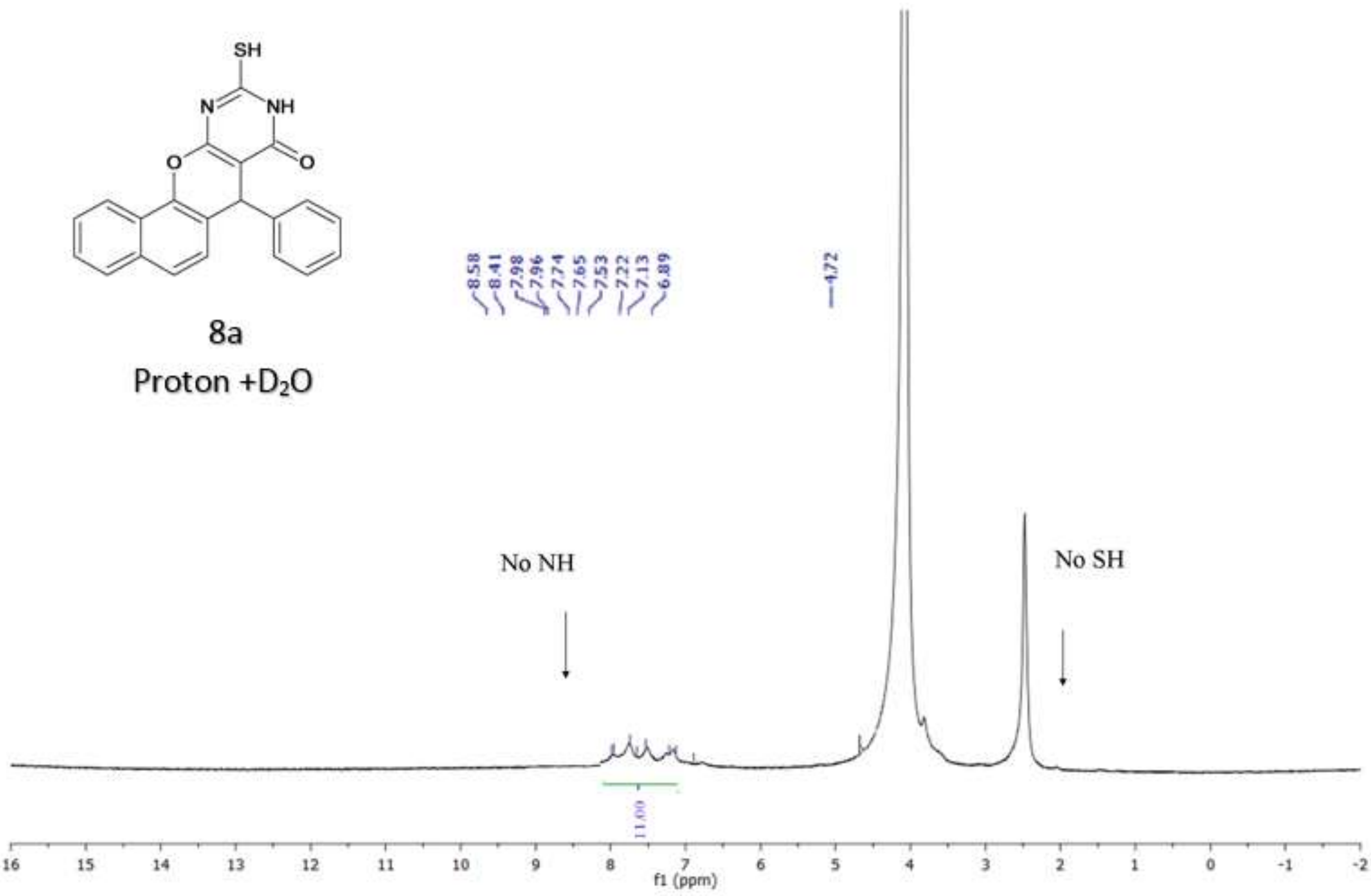

Information Classification: General

8b

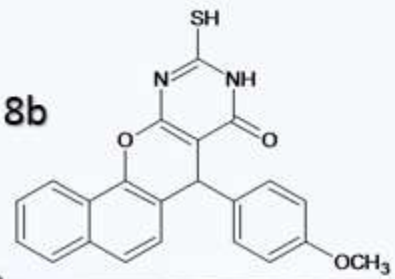

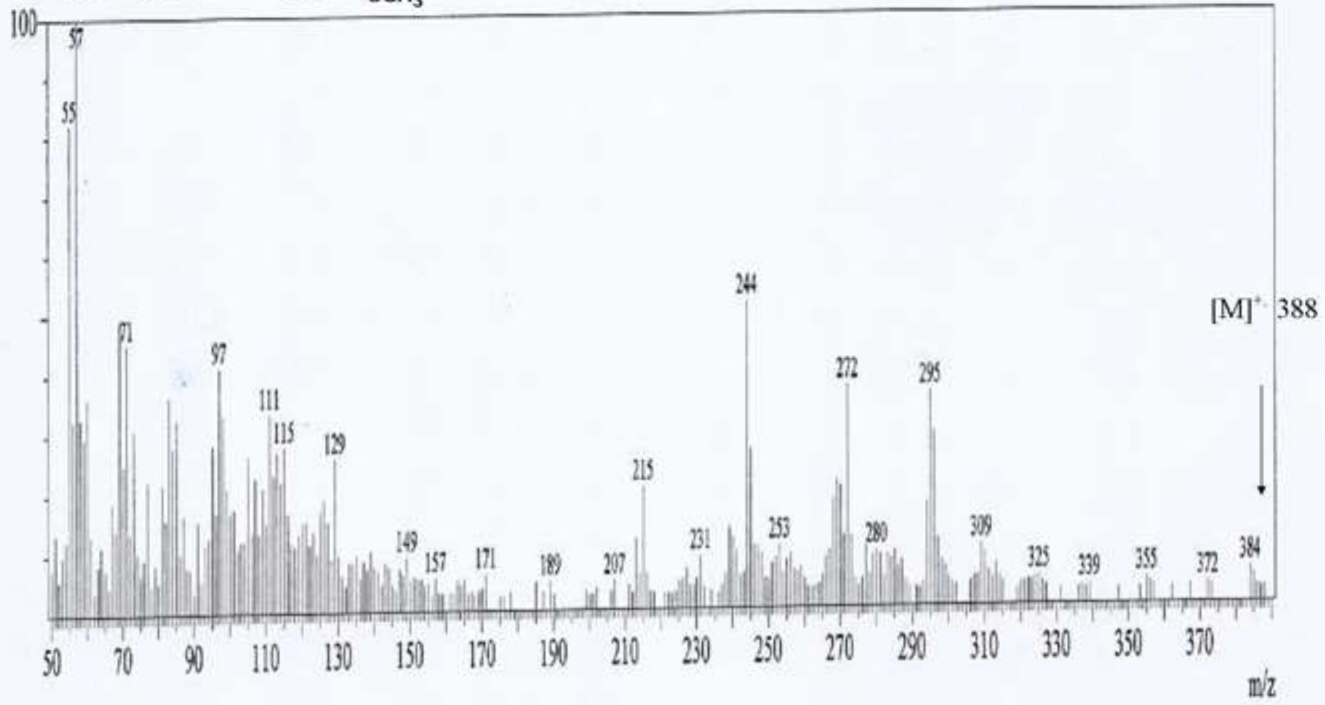

Information Classification: General

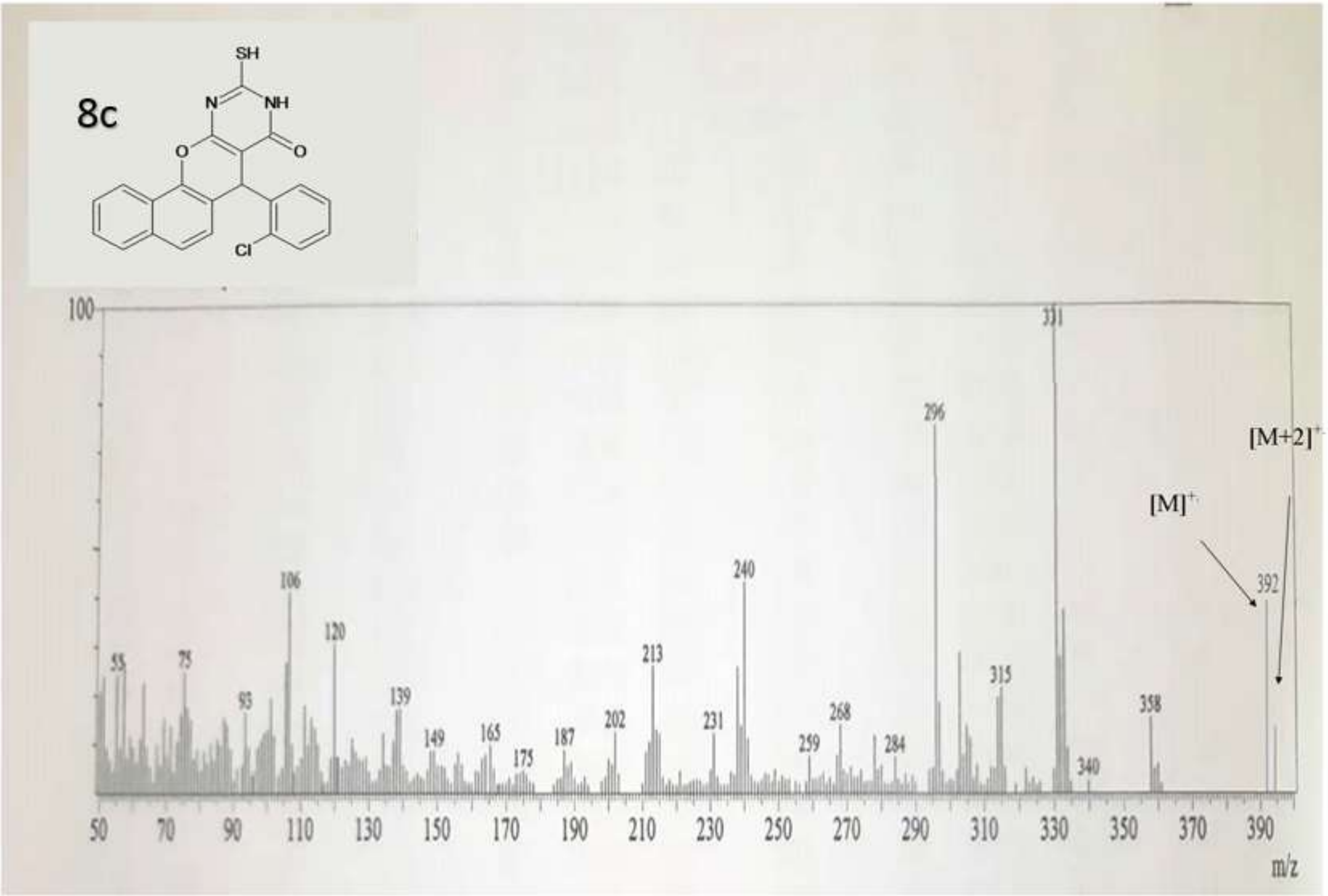
8c
SH
N
NH
O
O
Cl
100
331
296
[M+2]+.
[M]+.
392
240
106
120
213
315
358
55
75
93
139
149
165
175
187
202
231
259
268
284
340
50
70
90
110
130
150
170
190
210
230
250
270
290
310
330
350
370
390
m/z

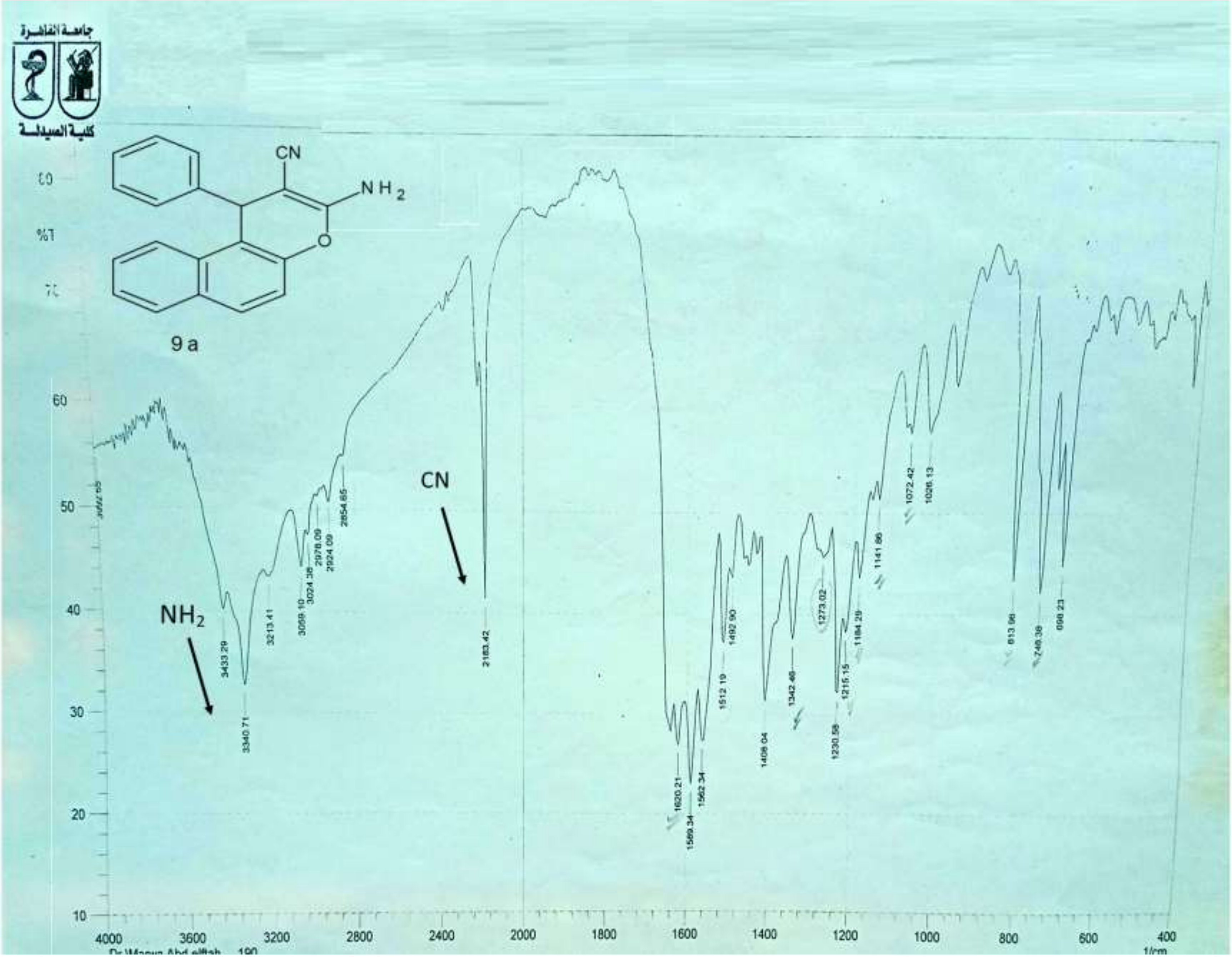

Information Classification: General

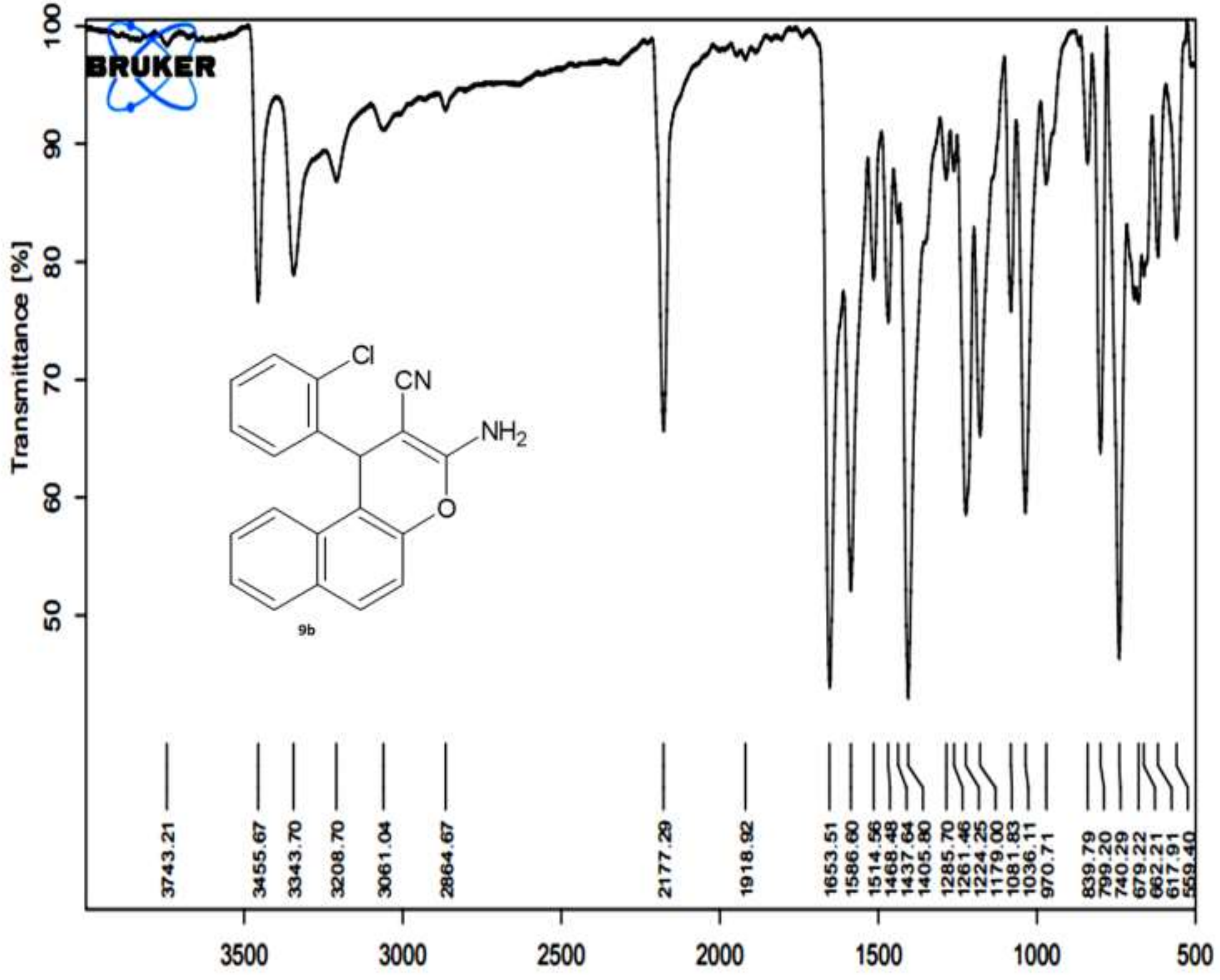

Information Classification: General

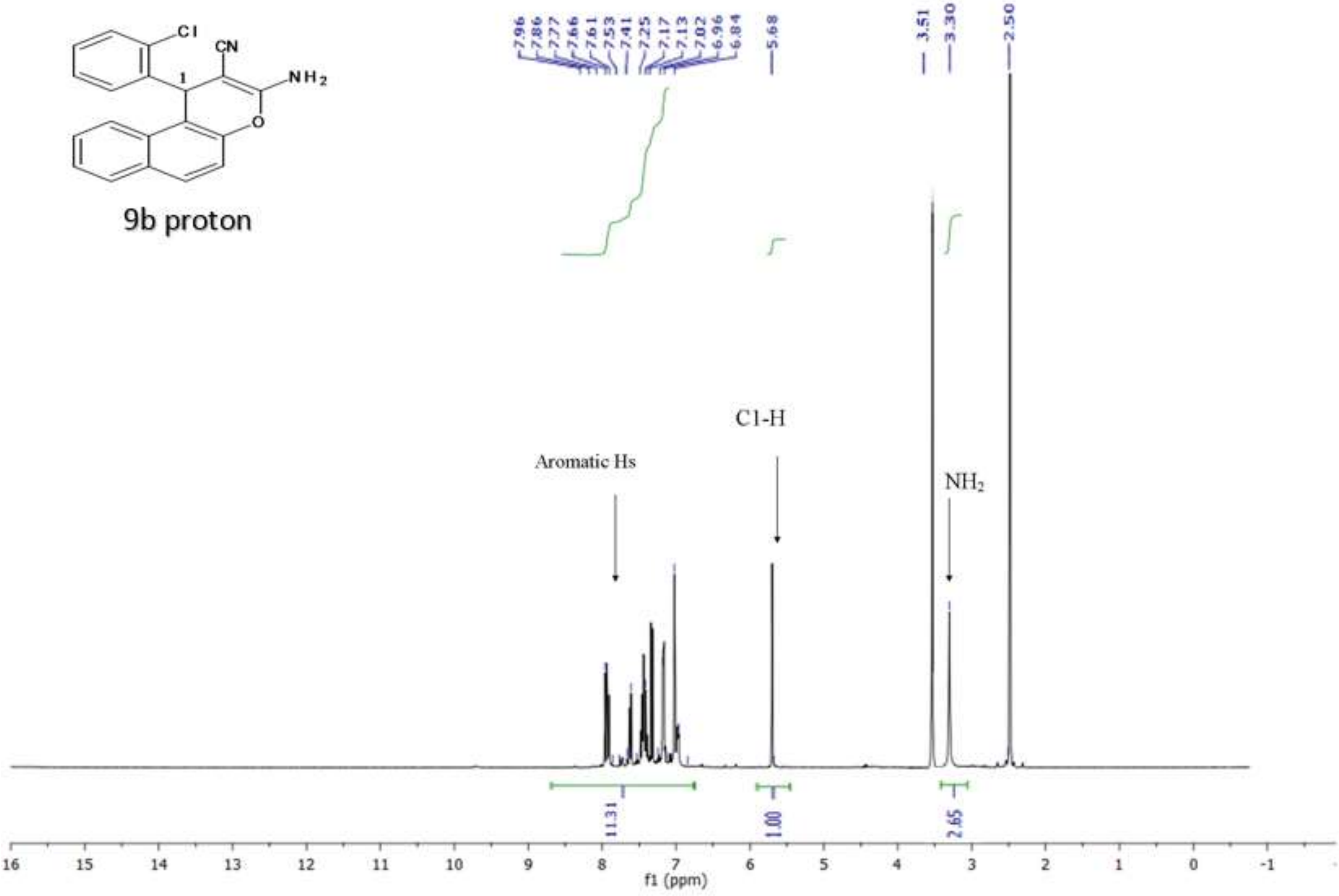

9b proton
Aromatic Hs
C1-H
NH_2

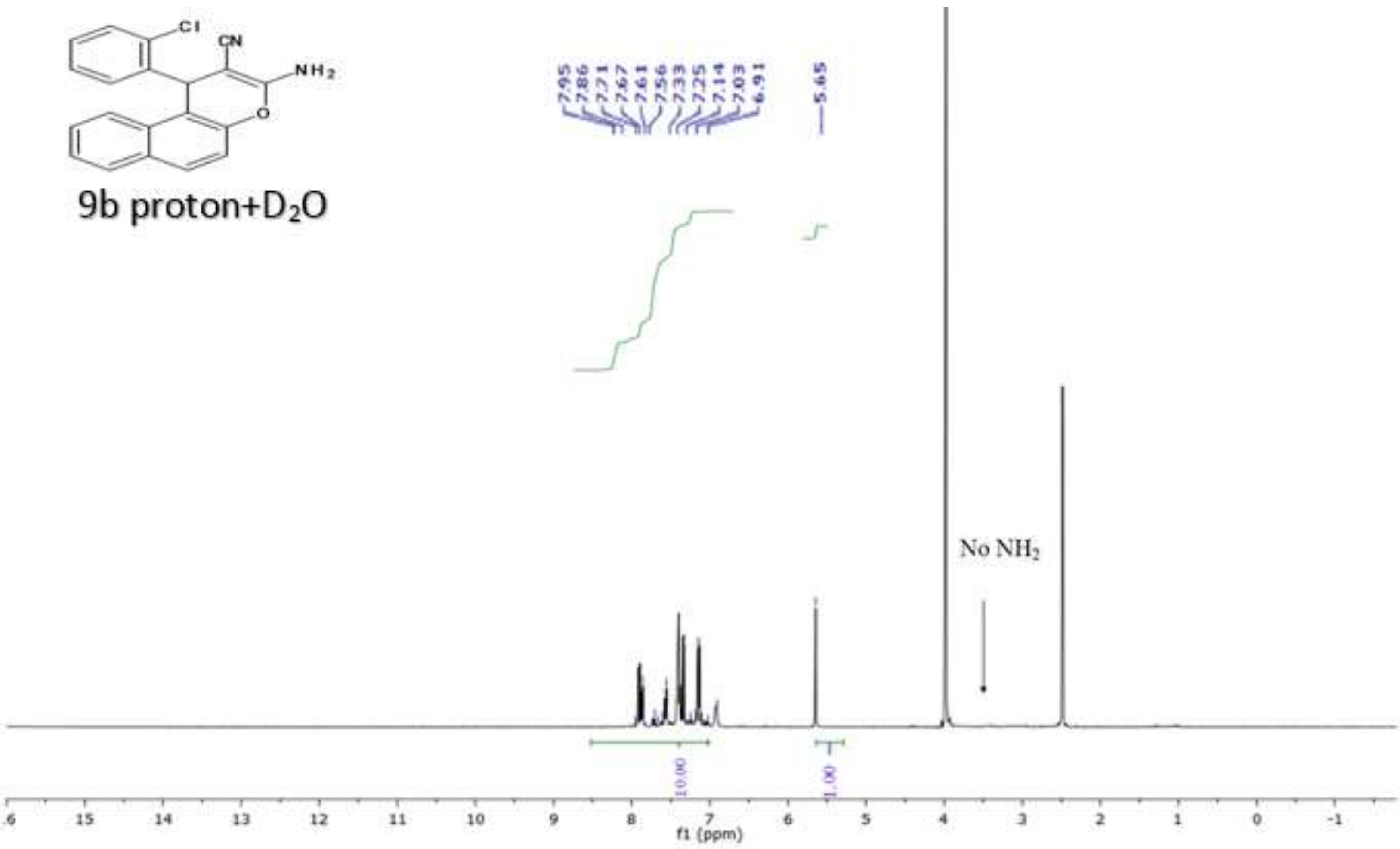

Information Classification: General

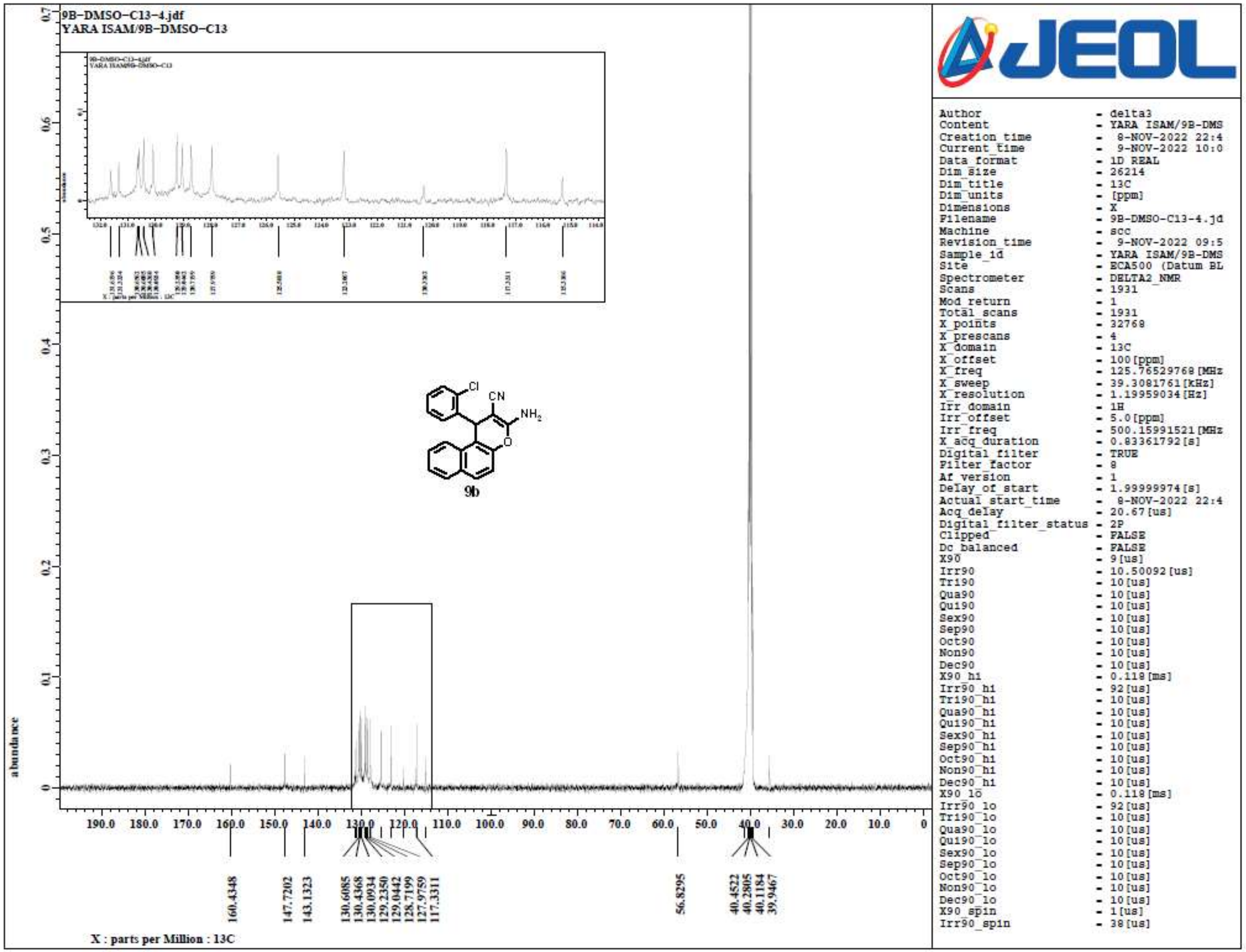

Author	-	delta3
Content	-	YARA ISAM/9B-DMS
Creation_time	-	8-NOV-2022 22:4
Current_time	-	9-NOV-2022 10:0
Data_format	-	1D REAL
Dim_size	-	26214
Dim_title	-	13C
Dim_units	-	[ppm]
Dimensions	-	X
Filename	-	9B-DMSO-C13-4.jd
Machine	-	scc
Revision_time	-	9-NOV-2022 09:5
Sample_id	-	YARA ISAM/9B-DMS
Site	-	ECA500 (Datum BL
Spectrometer	-	DELTA2_NMR
Scans	-	1931
Mod_return	-	1
Total_scans	-	1931
X_points	-	32768
X_prescans	-	4
X_domain	-	13C
X_offset	-	100[ppm]
X_freq	-	125.76529768[MHz
X_sweep	-	39.3081761[kHz]
X_resolution	-	1.19959034[Hz]
Irr_domain	-	1H
Irr_offset	-	5.0[ppm]
Irr_freq	-	500.15991521[MHz
X_acq_duration	-	0.83361792[s]
Digital_filter	-	TRUE
Filter_factor	-	8
Af_version	-	1
Delay_of_start	-	1.99999974[s]
Actual_start_time	-	8-NOV-2022 22:4
Acq_delay	-	20.67[us]
Digital_filter_status	-	2P
Clipped	-	FALSE
Dc_balanced	-	FALSE
X90	-	9[us]
Irr90	-	10.50092[us]
Tri90	-	10[us]
Qua90	-	10[us]
Qui90	-	10[us]
Sex90	-	10[us]
Sep90	-	10[us]
Oct90	-	10[us]
Non90	-	10[us]
Dec90	-	10[us]
X90_hi	-	0.118[ms]
Irr90_hi	-	92[us]
Tri90_hi	-	10[us]
Qua90_hi	-	10[us]
Qui90_hi	-	10[us]
Sex90_hi	-	10[us]
Sep90_hi	-	10[us]
Oct90_hi	-	10[us]
Non90_hi	-	10[us]
Dec90_hi	-	10[us]
X90_lo	-	0.118[ms]
Irr90_lo	-	92[us]
Tri90_lo	-	10[us]
Qua90_lo	-	10[us]
Qui90_lo	-	10[us]
Sex90_lo	-	10[us]
Sep90_lo	-	10[us]
Oct90_lo	-	10[us]
Non90_lo	-	10[us]
Dec90_lo	-	10[us]
X90_spin	-	1[us]
Irr90_spin	-	38[us]

Information Classification: General

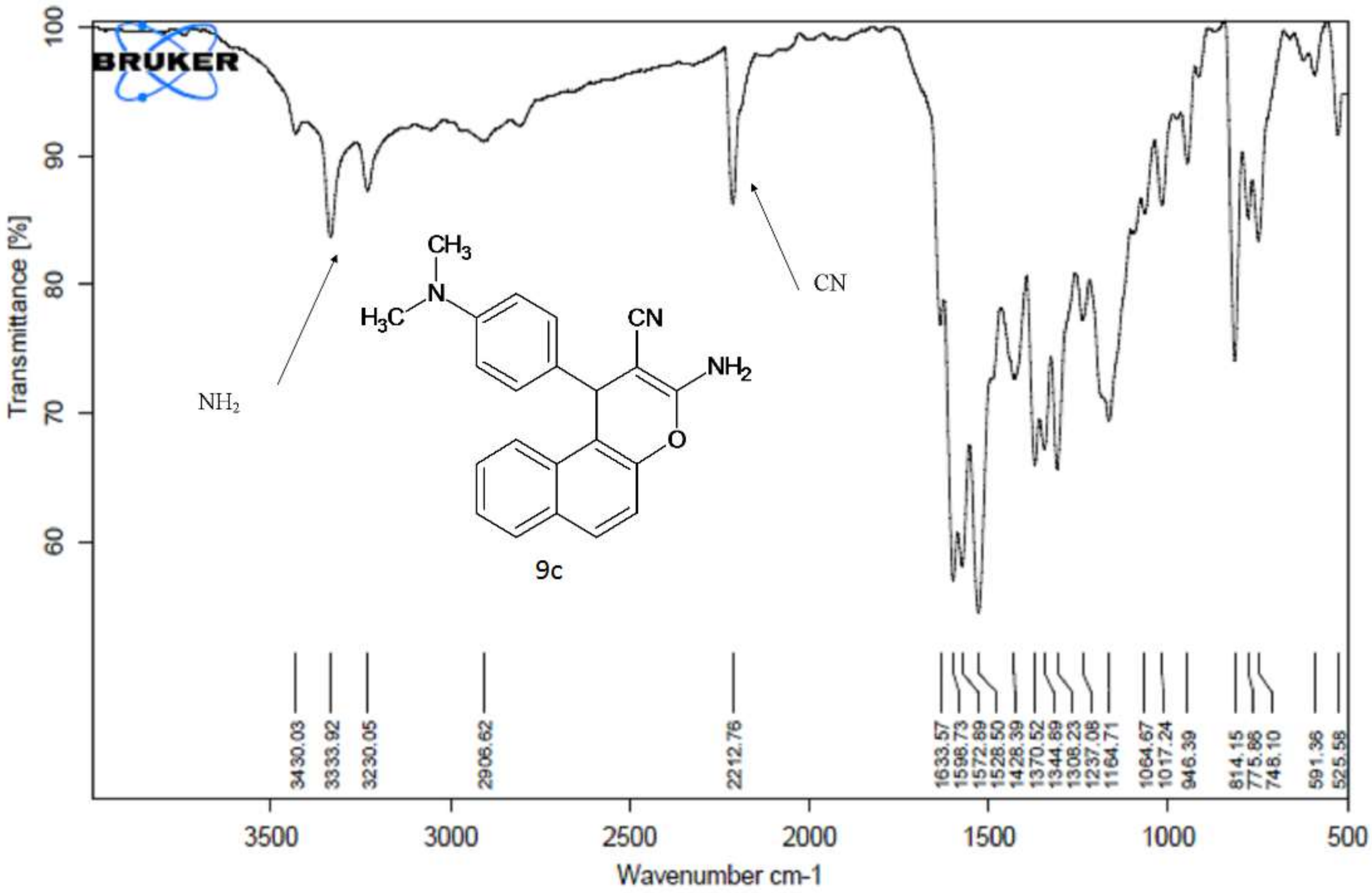

Information Classification: General

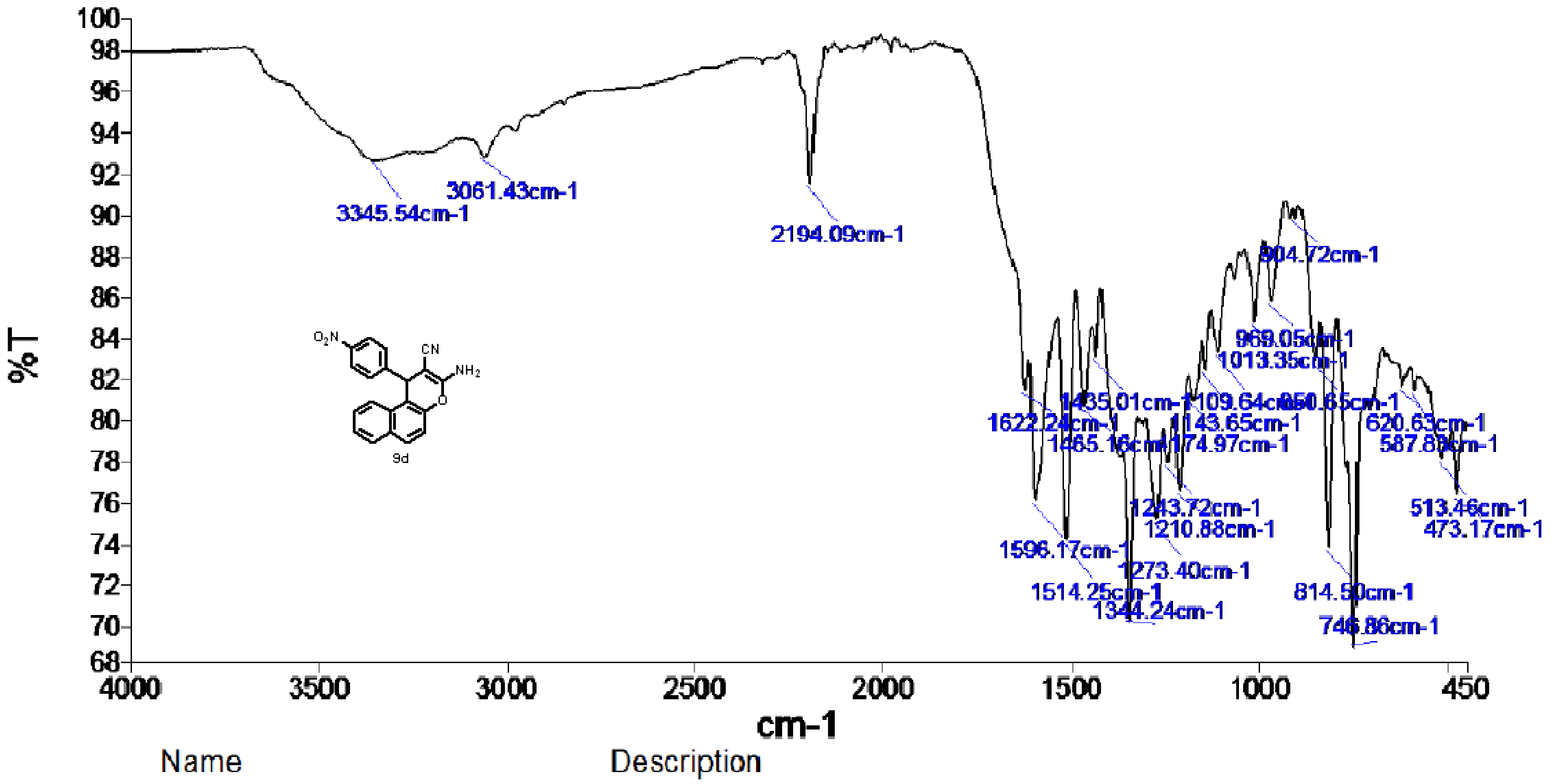
100
98
96
94
92
90
88
86
84
82
80
78
76
74
72
70
68
%T
4000
3500
3000
2500
2000
1500
1000
450
cm-1
3345.54cm-1
3061.43cm-1
2194.09cm-1
904.72cm-1
969.05cm-1
1013.35cm-1
1435.01cm-1
1109.64cm-1
850.65cm-1
1622.24cm-1
1143.65cm-1
620.63cm-1
1465.16cm-1
1174.97cm-1
587.83cm-1
1243.72cm-1
513.46cm-1
1210.88cm-1
473.17cm-1
1596.17cm-1
1273.40cm-1
1514.25cm-1
814.50cm-1
1344.24cm-1
746.86cm-1
O_2N
CN
NH_2
O
9d
Name
Description

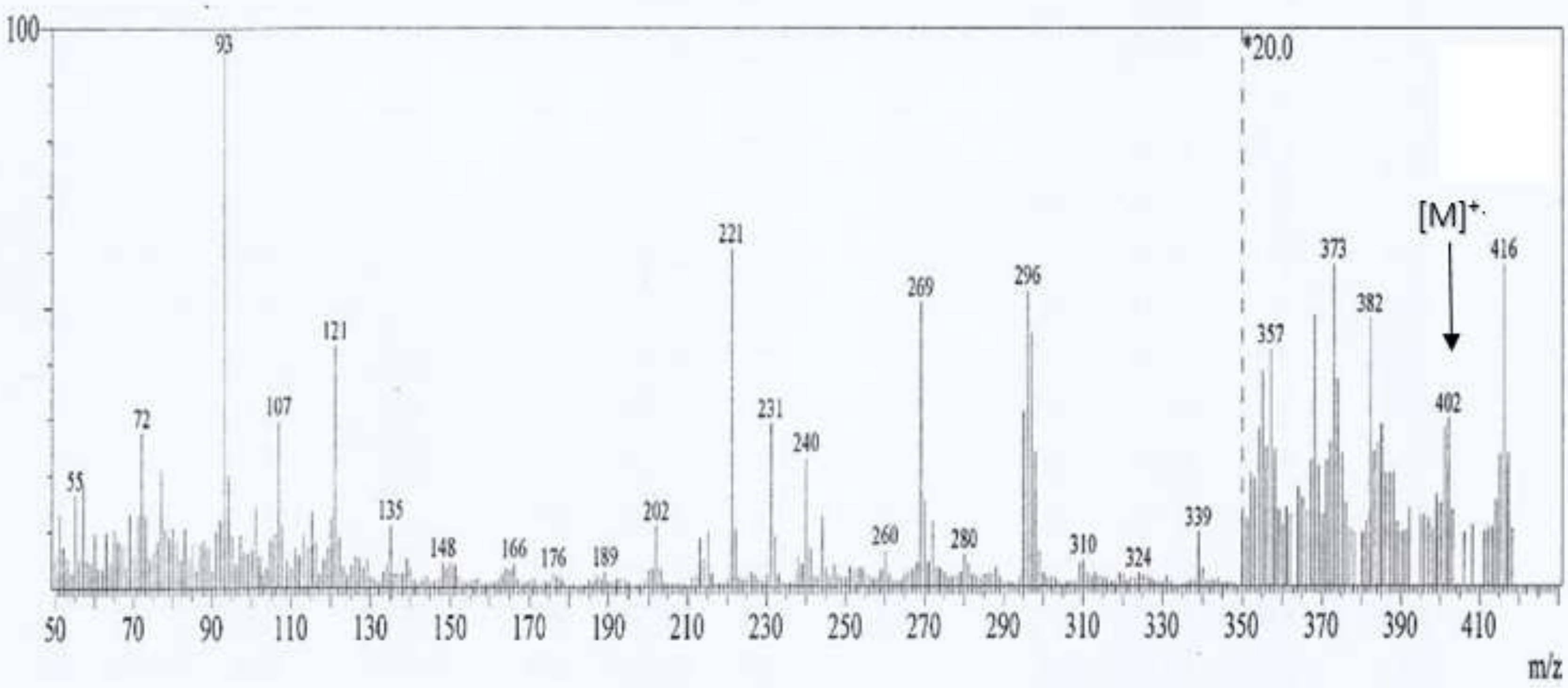

10a

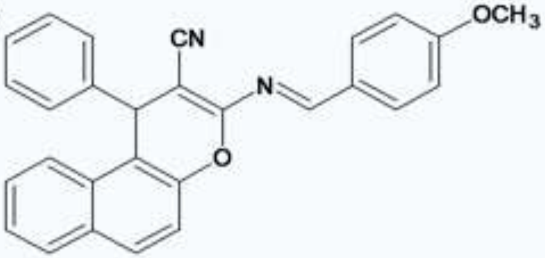

Information Classification: General

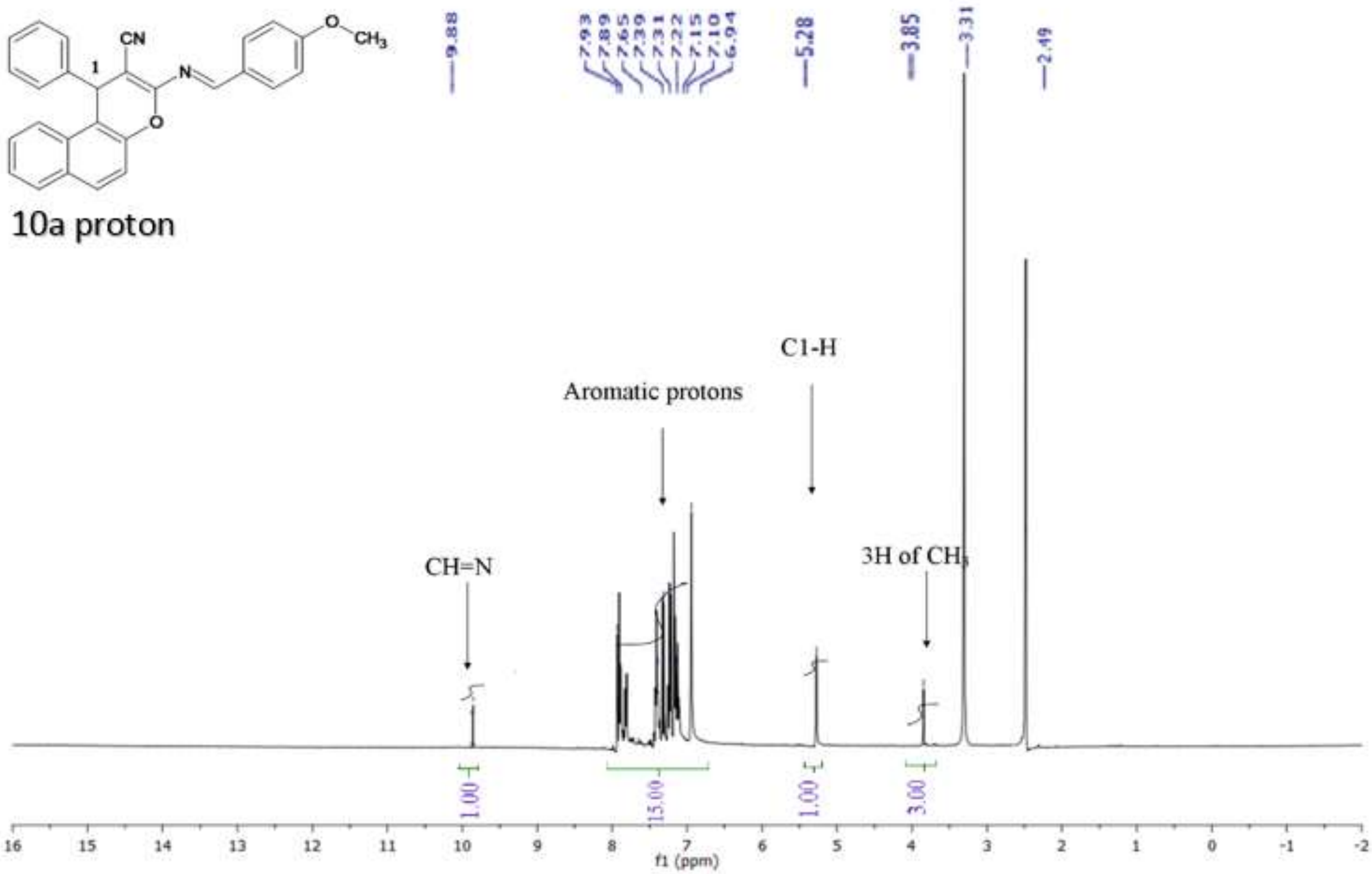

Information Classification: General

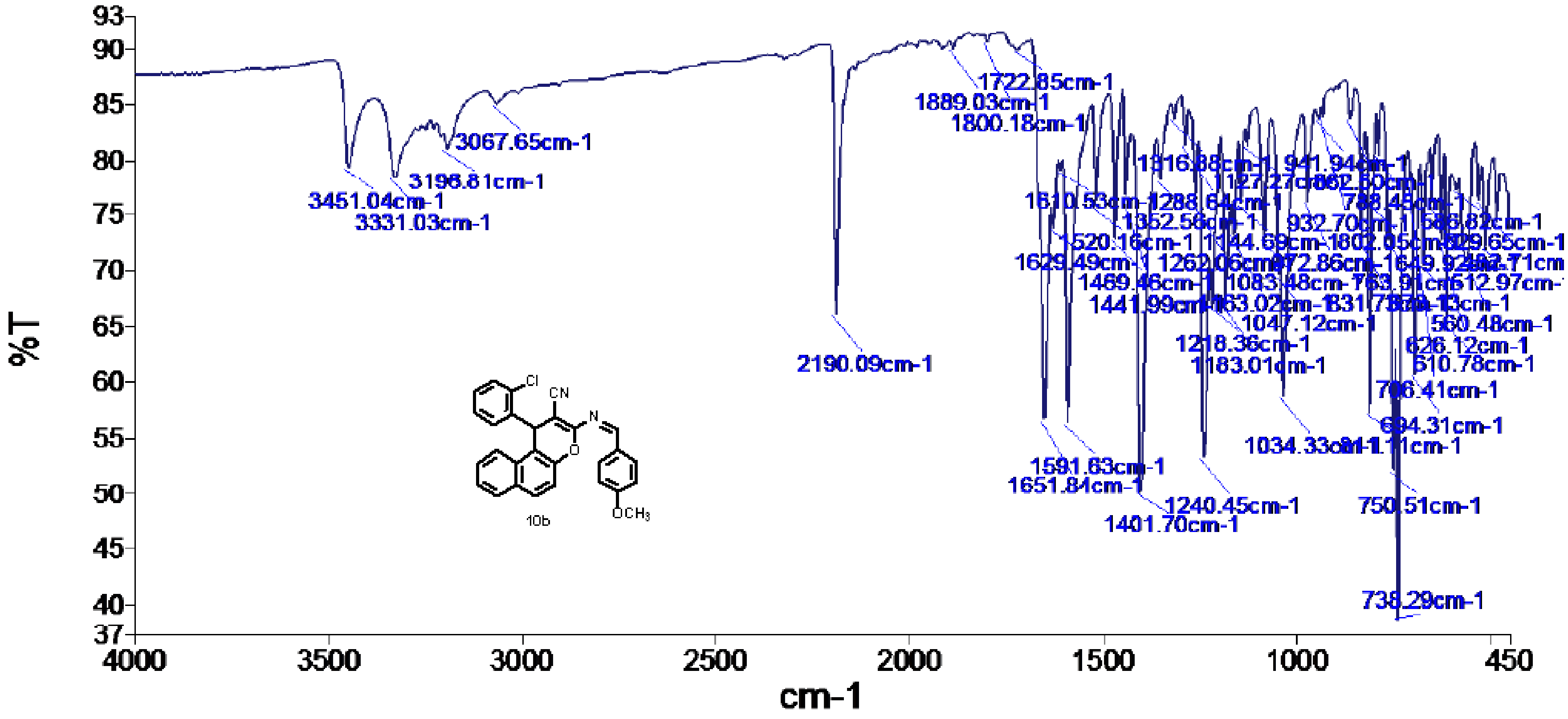

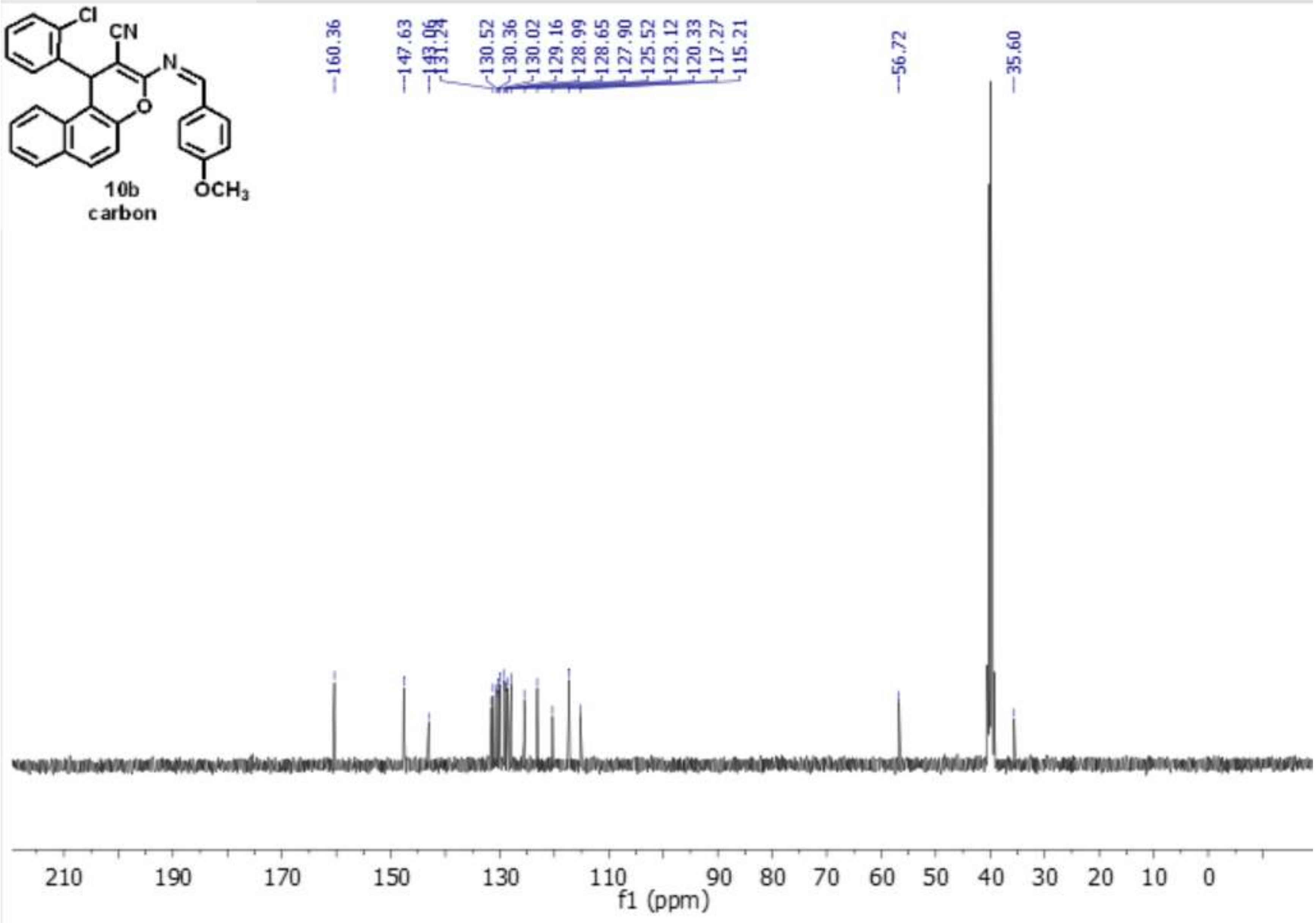
Cl
CN
N
O
10b
carbon
OCH3
160.36
147.63
143.06
131.24
130.52
130.36
130.02
129.16
128.99
128.65
127.90
125.52
123.12
120.33
117.27
115.21
56.72
35.60
210
190
170
150
130
110
90
80
70
60
50
40
30
20
10
0
f1 (ppm)

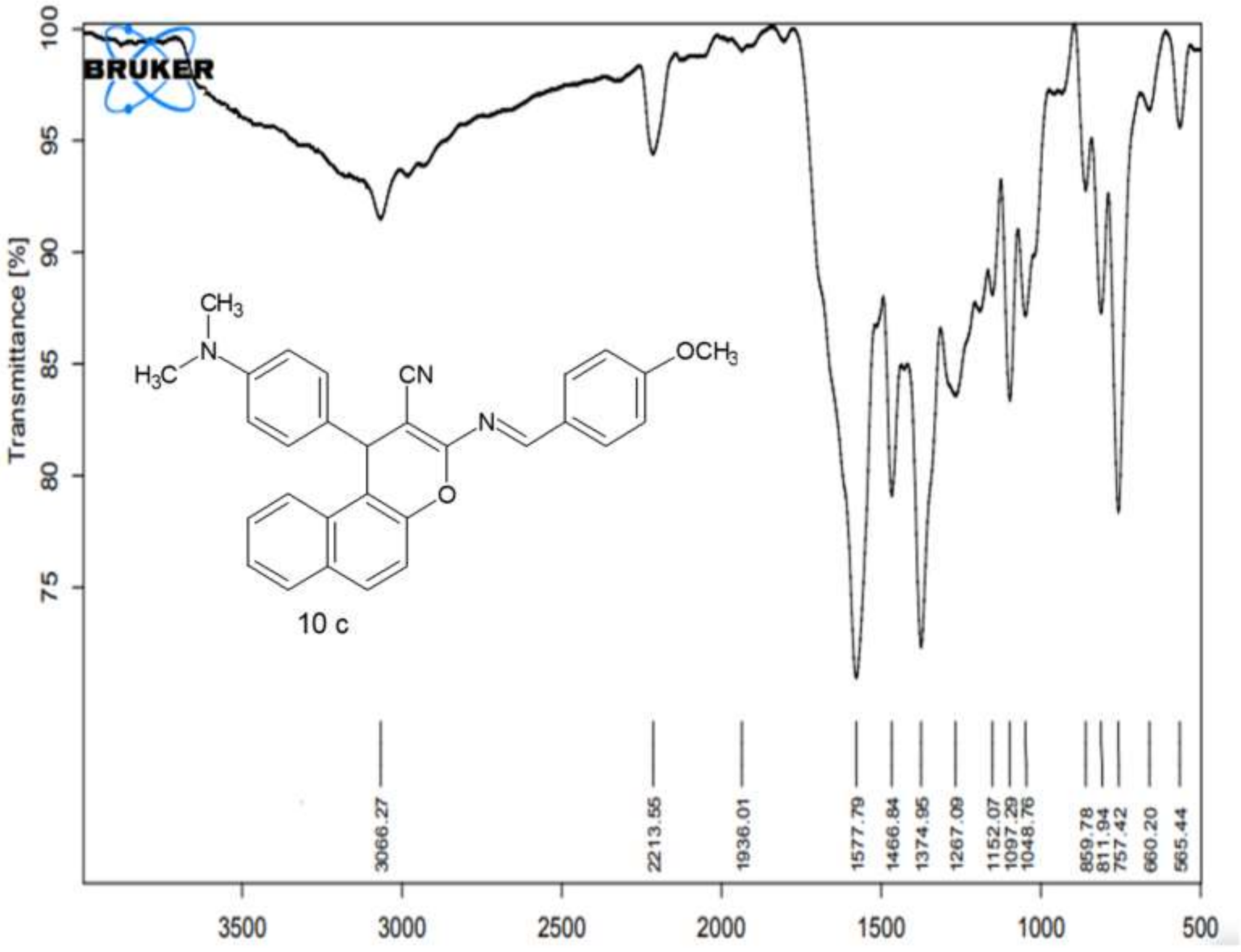

BRUKER
Transmittance [%]
100
95
90
85
80
75
CH3
H3C
N
CN
N
OCH3
O
10 c
3066.27
2213.55
1936.01
1577.79
1466.84
1374.95
1267.09
1152.07
1097.29
1048.76
859.78
811.94
757.42
660.20
565.44
3500
3000
2500
2000
1500
1000
500

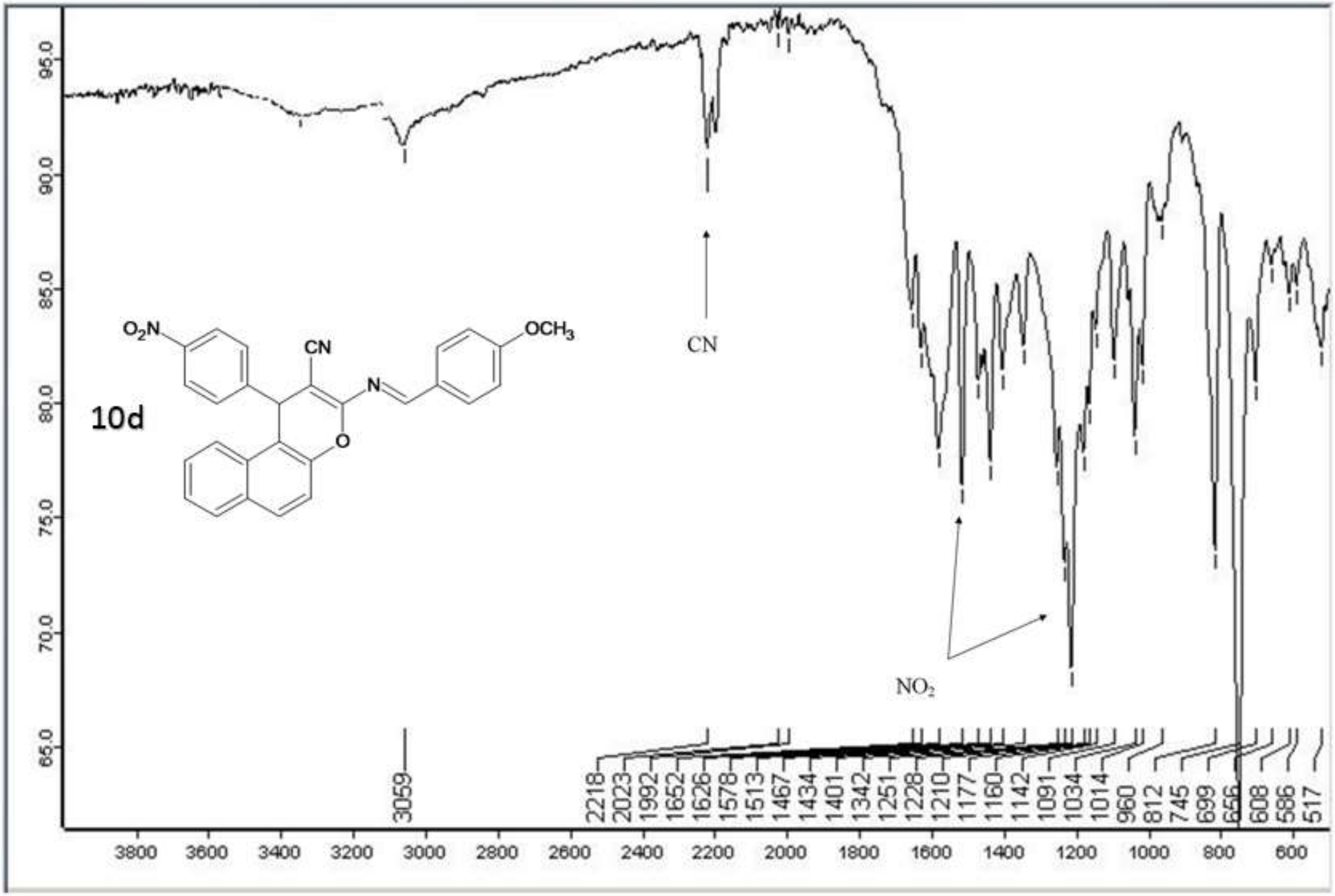
10d
CN
NO2
3059
2218
2023
1992
1652
1626
1578
1513
1467
1434
1401
1342
1251
1228
1210
1177
1160
1142
1091
1034
1014
960
812
745
699
656
608
586
517

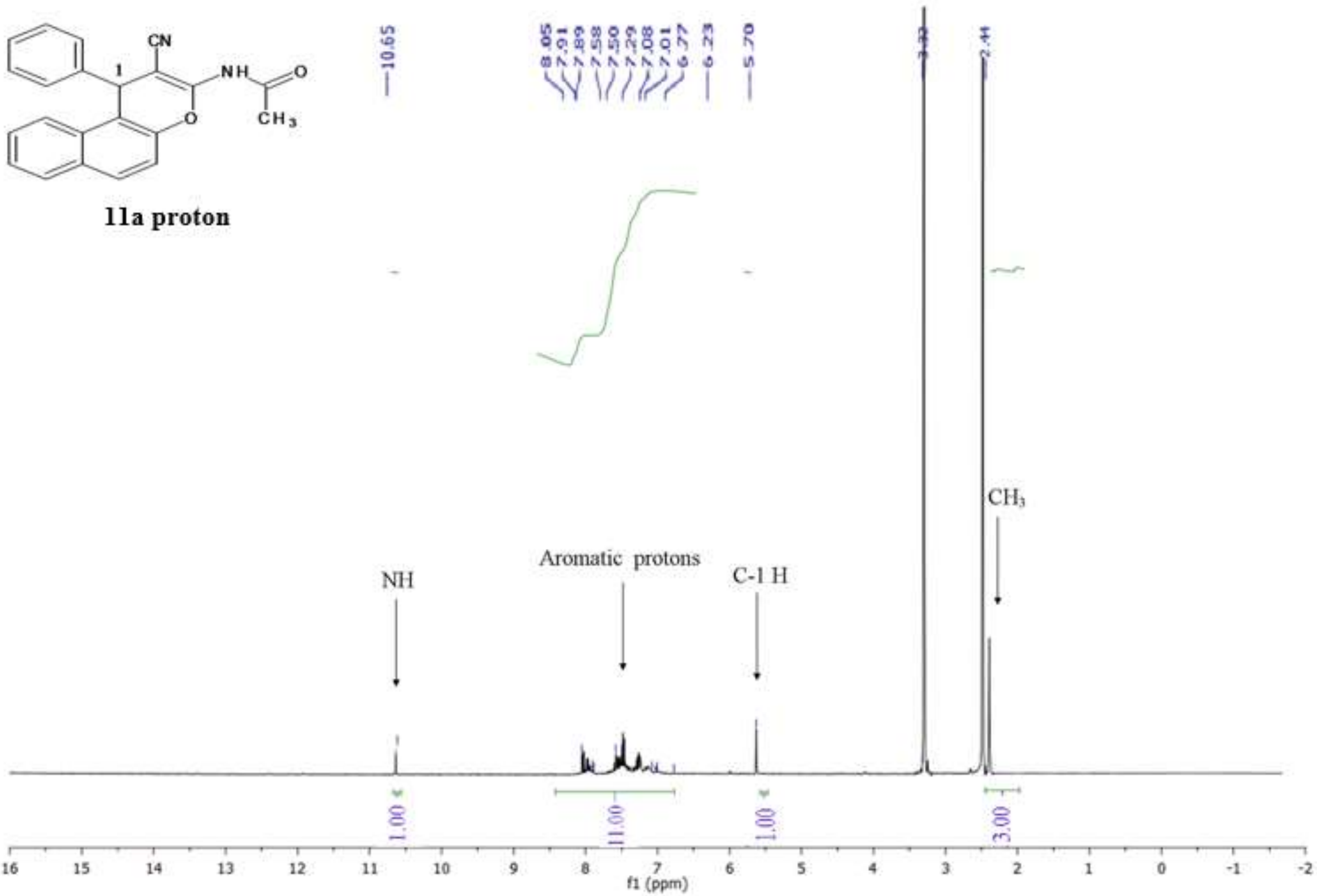

Information Classification: General

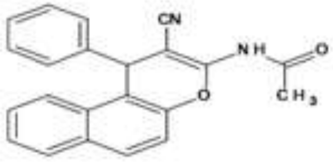

11a proton +D_2O

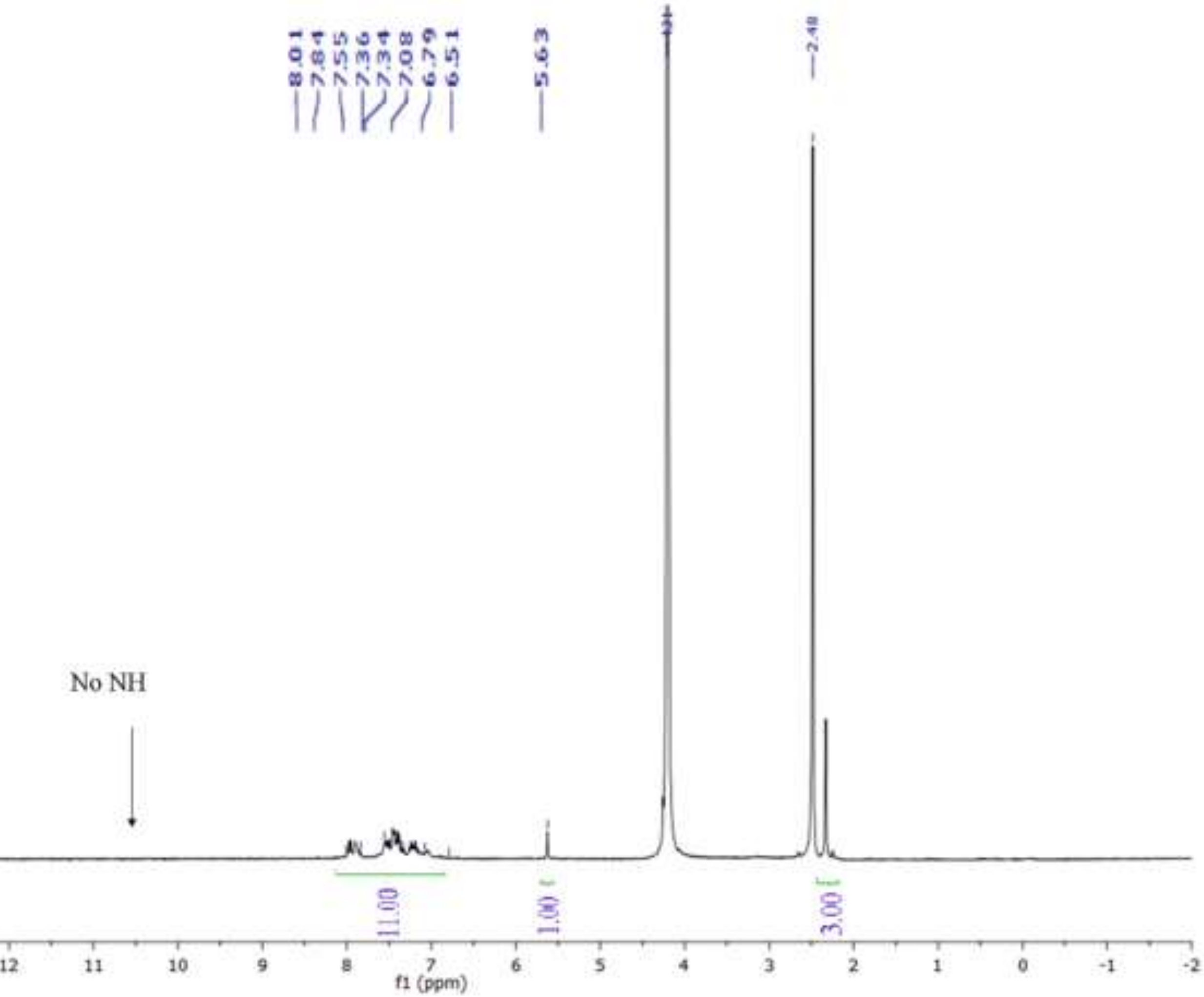

Information Classification: General

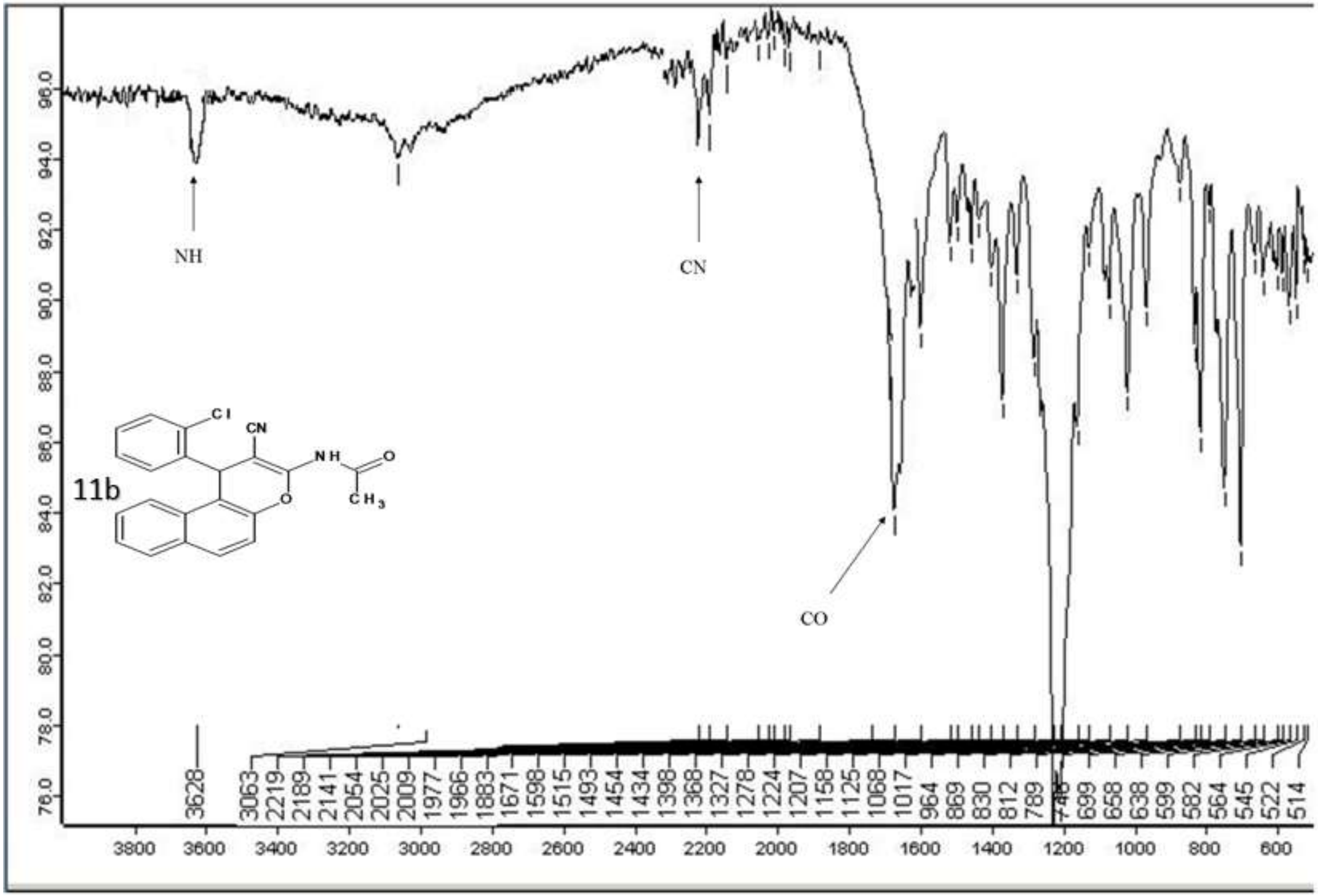

Information Classification: General

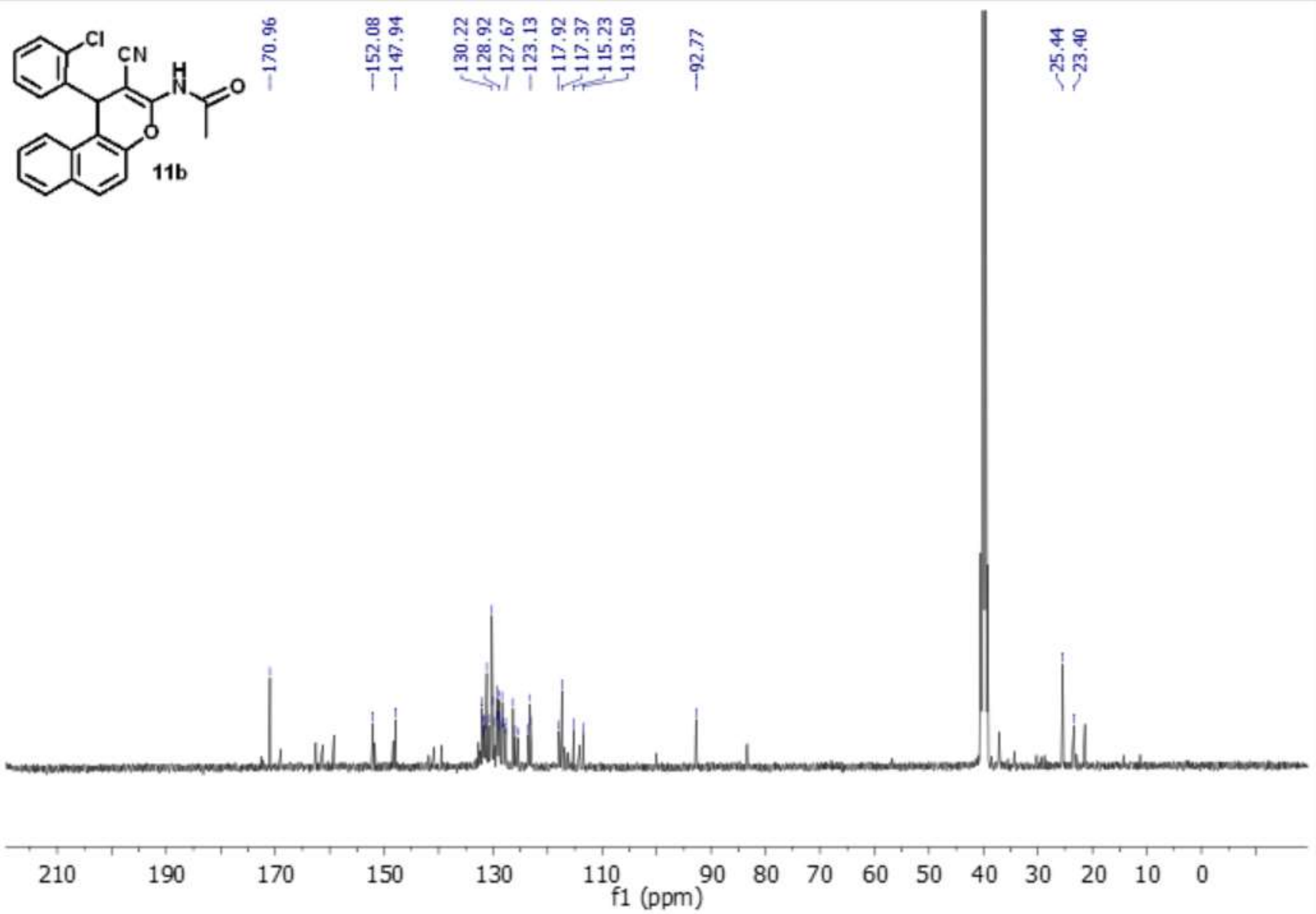

Cl
CN
H
N
O
O
11b
170.96
152.08
147.94
130.22
128.92
127.67
123.13
117.92
117.37
115.23
113.50
92.77
25.44
23.40
210
190
170
150
130
110
90
80
70
60
50
40
30
20
10
0
f1 (ppm)
